# A Dichotomy in Cross-Coupling Site Selectivity in a Dihalogenated Heteroarene: Influence of Mononuclear Pd, Pd Clusters, and Pd Nanoparticles—the Case for Exploiting Pd Catalyst Speciation

**DOI:** 10.1021/jacs.1c05294

**Published:** 2021-06-21

**Authors:** Neil W. J. Scott, Mark J. Ford, Neda Jeddi, Anthony Eyles, Lauriane Simon, Adrian C. Whitwood, Theo Tanner, Charlotte E. Willans, Ian J. S. Fairlamb

**Affiliations:** † Department of Chemistry, University of York, Heslington, York, North Yorkshire YO10 5DD, United Kingdom; ‡ Bayer AG, Alfred-Nobel-Strasse 50, 40789 Monheim, Germany; § School of Chemistry, University of Leeds, Woodhouse Lane, Leeds LS2 9JT, United Kingdom

## Abstract

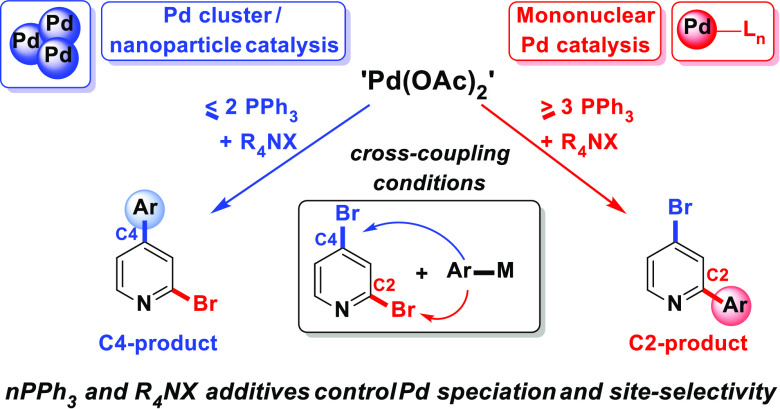

Site-selective dihalogenated heteroarene cross-coupling with organometallic reagents usually occurs at the halogen proximal to the heteroatom, enabled by intrinsic relative electrophilicity, particularly in strongly polarized systems. An archetypical example is the Suzuki–Miyaura cross-coupling (SMCC) of 2,4-dibromopyridine with organoboron species, which typically exhibit C2-arylation site-selectivity using mononuclear Pd (pre)catalysts. Given that Pd speciation, particularly aggregation, is known to lead to the formation of catalytically competent multinuclear Pd_*n*_ species, the influence of these species on cross-coupling site-selectivity remains largely unknown. Herein, we disclose that multinuclear Pd species, in the form of Pd_3_-type clusters and nanoparticles, switch arylation site-selectivity from C2 to C4, in 2,4-dibromopyridine cross-couplings with both organoboronic acids (SMCC reactions) and Grignard reagents (Kumada-type reactions). The Pd/ligand ratio and the presence of suitable stabilizing salts were found to be critically important in switching the site-selectivity. More generally, this study provides experimental evidence that aggregated Pd catalyst species not only are catalytically competent but also alter reaction outcomes through changes in product selectivity.

## Introduction

**Scheme 1:**
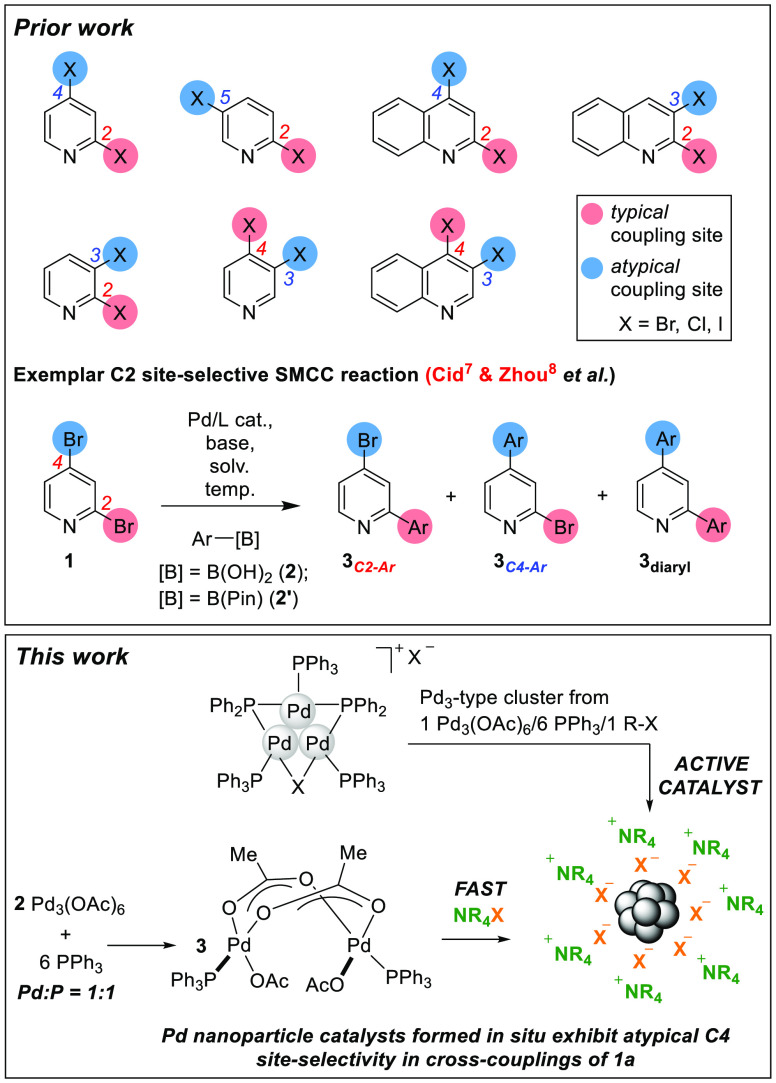
Site-Selectivity in Suzuki–Miyaura Cross-Couplings of Heteroarenes, Exemplified by Dihalogenated Pyridines and Related Derivatives A guiding example, for which many catalyst systems/reaction conditions have been investigated, is given, showing high C2 site-selectivity.

Dihalogentated organic compounds, particularly heteroarenes, serve as synthetically useful structural templates for increasing molecular complexity. They enable multiple modes of connectivity, providing access to a vast array of compounds with interesting properties, from agrochemicals and pharmaceuticals to advanced materials.^1,2^ Classical cross-coupling reaction methodologies are powerful tools for enabling site-selective processes to be realized, as outlined in two critical reviews by Fairlamb in 2007^3^ and Spivey et al. in 2017.^4^ Leading examples are given in Scheme 1, showing the preferred cross-coupling site for a series of dihalogenated heteroarenes. Normally, site-selectivity is seen at halogens activated by the ring heteroatom, either through proximity or favorable bond polarization in the extended π-ring system. Houk et al. explained the origin of normal site-selectivity in the context of the distortion of the C–X bond from a given substrate and interaction energies on approach to the active Pd^0^L_*n*_ catalyst.^5^ Consideration can further be made for the bond dissociation energies (BDE) at different C–X bonds. Handy et al. demonstrated that cross-coupling site-selectivity could be predicted, with caveats, by comparing the ^1^H NMR chemical shifts of the parent heteroarene—the most deshielded proton being the typical site for coupling in the corresponding C–X derivative.^6^ Switching site-selectivity in the cross-coupling reactions of dihalogenated heteroarenes, which effectively possess biased intrinsic reactivity (through relative electrophilicity), is a difficult task. For 2,4-dibromopyridine **1**, it is very challenging, as C2 site-selectivity dominates as described in the extensive screening work carried out by Cid^7^ and Zhou et al.^8,9^ There are only a few examples where atypical C4 site-selectivity in cross-coupling is known.^10^ A C4 site-selective Suzuki–Miyaura cross-coupling example on **1** was reported by Hardie and Willans et al.,^11^ which employs Pd-NHC precatalysts, possessing distinctive ligand architectures. For the best precatalyst, C4:C2 site-selectivity was ∼10:1. However, as is common to an eclectic array of dihalogenated heteroarene substrates, diarylation was found to be a competing process and overall product yields were moderate as a consequence (∼35% for monoarylation product). Dai et al. switched the site-selectivity in Suzuki–Miyaura cross-coupling reactions (SMCCs) involving 2,4-dichloropyridine using a Q-Phos/Pd(OAc)_2_ precatalyst system, resulting in a marginal bias toward the atypical C4-arylated product, but accompanied by low yields.^12^ Higher C4-selectivities at 2,4-dichloropyridine were obtained by changes to exogenous ligands at Pd, as reported in 2020 by Yang et al.^13^

The background literature therefore highlights that switches from typical to atypical site-selectivity are feasible, but that fundamental reasoning is frustratingly lacking—the focus has often been placed on ligand changes, assuming a mononuclear Pd catalyst.^14−17^ While logical, in our opinion Pd catalyst speciation is a bigger issue, where changes in mechanism might better account for typical to atypical site-selectivity changes.

Our research group has been engaged in understanding the role played by catalytically competent aggregated Pd clusters and nanoparticles in SMCCs, and related cross-couplings, for many years.^18−23^ We presented the first compelling experimental evidence implicating heterogeneous surface catalysts in SMCCs,^24,25^ which is supported by recent evidence using time-resolved fluorescence studies^26^ and surface-enhanced Raman spectroscopic techniques.^27^

The knowledge outlined above is important in the context of understanding that mononuclear Pd species, generally thought to be the dominant catalytically active species in SMCCs, can aggregate to form higher order Pd nanoparticles that are capable of mediating further substrate turnover. A serious question facing the field of cross-coupling catalysis is the involvement of small Pd_*n*_ clusters (*n* < 13), as such species provide a potential bridge from mononuclear Pd_1_ species to Pd nanoparticles (PdNPs).^28^ Indeed, in a recent study Li et al.^29^ presented some evidence that [Pd_3_(μ-Cl)(μ-PPh_2_)_2_(PPh_3_)_3_]^+^^30,31^ not only was an active Pd catalyst for SMCCs but also appears to invert the order of the oxidative addition and transmetalation steps within the catalytic cycle, proposing the activation of the aryl halide as being less like oxidative addition and more like σ-bond metathesis. Our recent findings showed that similar [Pd_3_(μ-Cl)(μ-PPh_2_)_2_(PPh_3_)_3_]X cluster species derive from a Pd_3_(OAc)_6_/6PPh_3_ precatalyst, by reaction of an organohalide (R–X, including 2-bromopyridine) with the intermediate formed Pd^I^ dinuclear species.^32^ The outcome sparked our interest in understanding how higher order Pd species might affect site-selectivity in cross-coupling reactions of 2,4-dibromopyridine **1** with organoboronic acids **2**, as well as other nucleophiles, such as Grignard reagents. We were encouraged as [Pd_3_(μ-Cl)(μ-PPh_2_)_2_(PPh_3_)_3_]X species were found to be more active in the reported SMCC reactions than Pd^0^(PPh_3_)_3_ (in terms of substrate turnover frequency). [Pd_3_(μ-X)(μ-PR_2_)_2_(PR_3_)_3_]X species have been invoked as catalytically relevant species under a range of conditions.^29,33,34^

Schoenebeck et al. have investigated the use of multinuclear catalysts for chemoselective cross-coupling reactions at substrates containing two or more *different* (pseudo)halide identities. For example, the reactivity of [Pd(μ-I)(P*t*-Bu_3_)]_2_ enabled successive selective couplings at Br then OTf then Cl sites on aromatic substrates.^35,36^ A Pd_3_ cluster catalyst, derived from highly active [Pd(μ-Br)(P*t*-Bu_3_)]_2_,^37^ facilitated selective cross-couplings at aryl iodide over the less activated aryl bromide sites.^34^ Additionally, a nanoparticulate active catalyst, derived *in situ* from Pd_2_(dba)_3_, was found to enable chemoselective cross-couplings between aryl iodides and arylgermanes.^38^ Despite the clear synthetic utility of chemoselective reactions at multiply halogenated compounds for rapid molecular diversification, the preferential site of cross-coupling is generally quite clear-cut, defined by the BDE of the C–X bond (e.g., for halides (X), I < Br < Cl ≪ F).^3,4^ We note that regioselective control of cross-coupling at substrates featuring multiple halogens of *the same* type (i.e., with similar BDEs) constitutes a greater challenge than a chemoselective approach involving different halogens.

In this paper we examine the behavior of Pd_3_-type cluster and Pd nanoparticle catalysts that derive from Pd(OAc)_2_/nPPh_3_ precatalyst systems under working reaction conditions. Varying the number of PPh_3_ ligands (relative to Pd) enables us to switch between higher order Pd_*n*_ catalysis and mononuclear Pd_1_ catalysis. This has an impact on switching regioselectivity—*the reaction outcome*—from typical C2 to atypical C4, in 2,4-dibromopyridine **1** cross-couplings with either organoboronic acids **2** (SMCC reactions) or Grignard reagents **5** (Kumada–Corriu type reactions). The activity of PdNPs is modulated by additive stabilizing salts, which proved to be critical in switching catalyst site-selectivity. While PdNPs are established cross-coupling catalysts, this is the first time that site-selectivity in a dihalogenated heteroarene has been reversed through exploitation of conditions that facilitate the *in operando* (under working reaction conditions) generation of Pd nanoparticles.

## Results and Discussion

**Scheme 2:**
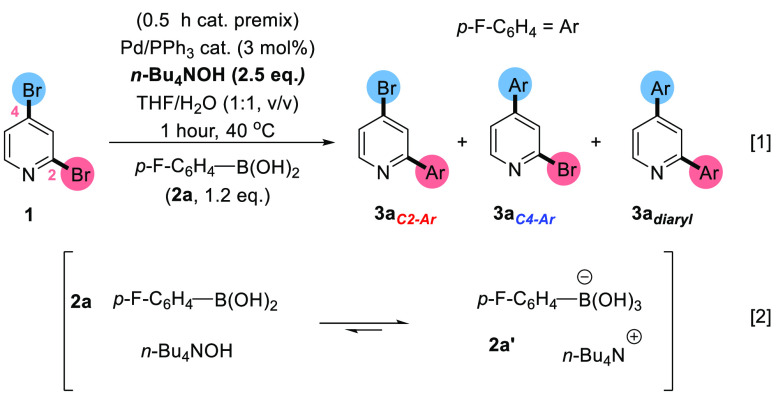
Benchmark SMCC of **1** with *p*-Fluoro-phenylboronic Acid **2a** To Give Typical Product **3a**_***C2-Ar***_, Atypical Product **3a**_***C4-Ar***_, and Diarylated Product **3a**_***diaryl***_ [1]; the Proposed Equilibrium for **2a** and *n-*Bu_4_NOH, Which Is Expected to Lie to the Right-Hand Side Is Shown in [2]

A benchmark SMCC test reaction [1] is shown in Scheme 2, involving 2,4-dibromopyridine **1** and *p*-fluorophenyl boronic acid **2a** to give three products: **3a**_***C2–Ar***_, **3a**_***C4–Ar***_, and **3a**_***diaryl***_. The calculated bond dissociation energies for the C_2_–Br and C_4_–Br bonds in **1** were calculated to be 63.3 and 66.9 kcal mol^–1^ respectively (determined by Density Functional Theory calculations using the B3LYP/DGTZVP level of theory), which indicate that the C_2_–Br bond is weaker that the C_4_–Br bond, mirroring the expected typical site for functionalization.

The reaction conditions described in Scheme 2 [1] are drawn from our earlier studies,^32^ informed by the work of Jutand et al.^39^ The reaction conditions benefit from being homogeneous (THF/H_2_O/[*n-*Bu_4_N]OH base at 40 °C, with a ratio of THF:H_2_O of 1:1). The high basicity ensures that the dominant boron species present in solution is the aryl boronate species **2a**′, stabilized by an *n*Bu_4_N^+^ cation [2].^40a,40b^ We anticipated the importance of this in terms of exploiting site-selectivity changes brought about by Pd catalyst speciation, under varying Pd/ligand ratios. Consistent with the findings reported by Cid,^7^ our reaction conditions employing Pd(PPh_3_)_4_ as the catalyst, gave rise to *typical* C2 site-selectivity at **1** although conversion was low at 40 °C. The latter finding parallels the low reactivity of 2-bromopyridine under identical conditions (i.e., the presence of higher PPh_3_ equivalents results in lower catalyst efficacy).^32^

**Figure 1:**
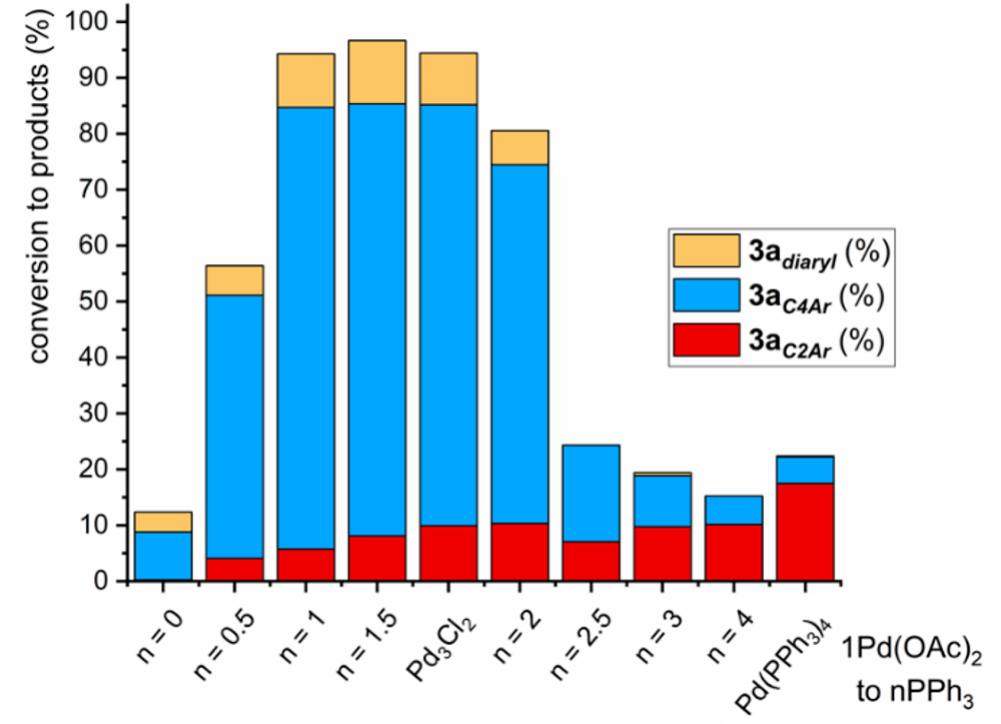
Summarizing Pd catalyst efficacy under different precatalytic Pd:PPh_3_ regimes, showing reaction conversions of which product selectivities for the SMCC (Scheme 2) of **1** with *p*-fluoro-phenylboronic acid **2a** to give typical product **3a**_***C2–Ar***_ and atypical product **3a**_***C4–Ar***_ and bis-arylated product **3a**_***diaryl***_.

C2-selectivity was also observed when employing Pd_2_(dba)_3_·CHCl_3_ (ca. 93% purity)^41^ with 2 or 4 equiv of PPh_3_ under the identical conditions {forming Pd^0^(dba)_3–*n*_/(PPh_3_)_*n*_ where *n* = 1 or 2}. Significant differences in catalyst efficacy were revealed using Pd_3_(OAc)_6_/PPh_3_ precatalyst ratios, hereafter referred to as Pd(OAc)_2_/nPPh_3_ (where *n* = 0.5 to 4) under conditions as summarized in Scheme 2. For each catalytic regime, the conversion of **1** to products **3a**_***C2–Ar***_, **3a**_***C4–Ar***_, and **3a**_***diaryl***_ is given in Figure 1 (note that competing homocoupling reactions/protodebromination or protodeborylation were not observable).

For the Pd(OAc)_2_/nPPh_3_ ratios of 1:3 or 1:4, C2-site selectivity was observed, giving **3a**_***C2–Ar***_ as the major product, an outcome consistent with that observed, including lower product conversions, for the ubiquitous Pd^0^(PPh_3_)_4_ catalyst system. It is well established that Pd^0^(PPh_3_)_*n*_ species (where *n* = 2 or 3), and/or anionic derivatives, are formed from the Pd(OAc)_2_/nPPh_3_ ratios of 1:3 or 1:4, respectively.^32,42−45^

Altering the Pd(OAc)_2_/*n*PPh_3_ ratio to *n* = 2.5 results in a switch in site-selectivity to the atypical **3a**_***C4–Ar***_ product. Concomitant with this switch in site-selectivity is an increase in substrate **1** conversion, an outcome particularly evident on lowering *n*PPh_3_ in the system to *n* < 2. The highest catalyst efficacy and C4-site selectivity are seen for Pd(OAc)_2_/nPPh_3_, in a 1:1 or 1:1.5 ratio. Of particular note is the activity observed for the [Pd_3_(μ-Cl)(μ-PPh_2_)_2_(PPh_3_)_3_]Cl cluster precatalyst (referred to as ‘**Pd**_**3**_**Cl**_**2**_’ in Figure 1). This latter finding correlates with the Pd/P ratio in the **Pd**_**3**_**Cl**_**2**_ cluster which contains three donating PPh_3_ ligands (1 PPh_3_ per Pd) and two pseudohalogen-like anionic PPh_2_ ligands (the average oxidation state per Pd being 4/3). Where *n* = 0, thus under an exogenous phosphine ligand-free regime, the reactivity drops off significantly, although overall **3a**_***C4–Ar***_ product selectivity is maintained. Hence, under our SMCC conditions, merely changing the Pd(OAc)_2_/*n*PPh_3_ ratio results in a switch in site-selectivity and catalyst efficacy, with markedly increased reaction conversions and higher selectivity for the atypical **3a**_***C4–Ar***_ product.

**Scheme 3:**
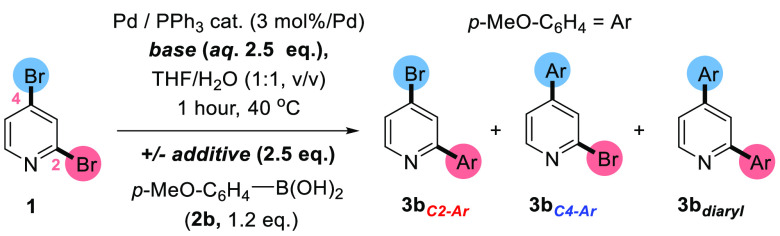
Testing Additive and Base Effects for the SMCC between **1** and **2b**

With the knowledge that Pd(OAc)_2_ and 1 equiv of PPh_3_ provided increased C4-site selectivity in SMCC reactions of **1**, we investigated whether other aspects of the conditions contributed to the atypical site-selectivities (Scheme 3) of **1** with *p*-anisylboronic acid (**2b**), which gave overall **3b**_***C4–Ar***_ selectivity under “benchmark” conditions (entry 1, Table 1).

**Table 1:** Modifying the Base and Additives in the SMCC Reaction between **1** and **2b** (Scheme 3)

Precatalyst	Entry	Base	Additive	Conv (%)^a^	**3b_C2–Ar_**:**3b_C4–Ar_**:**3b_diaryl_**^a^
Pd(OAc)_2_/1PPh_3_	1	*n*-Bu_4_NOH	none	100	10:70:20
	2	KOH	none	89	26:46:28
	3	KOH	*n*-Bu_4_NBr	88	3:70:26
Pd(OAc)_2_/2PPh_3_	4	*n*-Bu_4_NOH	none	100	18:58:24
	5	KOH	*n*-Bu_4_NBr	100	8:79:6
Pd_3_Cl_2_	6	*n*-Bu_4_NOH	none	100	15:69:16
	7	KOH	none	92	38:38:24
	8	KOH	*n*-Bu_4_NBr	94	9:82:10
	9	KOH	*n*-Oct_4_NBr	100	7:90:3

a Determined by ^1^H NMR analysis of the crude reaction mixture, after 1 h.

Using KOH (aq.) as the base in place of *n*-Bu_4_NOH (aq.) (entry 2, Table 1) resulted in a marginal reduction in conversion but, more strikingly, a marked reduction in site-selectivity, as exemplified by the reduced **3b**_***C4–Ar***_:**3b**_***C2–Ar***_ ratio when using a Pd(OAc)_2_/1PPh_3_ catalytic system. This observation indicated the cation of the base, *n*-Bu_4_NOH(aq.), as a critical factor in the higher site-selectivities observed. Indeed, employing KOH (aq.) base alongside an *n*-Bu_4_NBr additive increased the **3b**_***C4–Ar***_:**3b**_***C2–Ar***_ site-selectivity at the expense of relatively higher amounts of **3b**_***diaryl***_ (entry 3, Table 1). Cations have been shown to be able to influence SMCC reaction rates, principally the transmetalation step,^39,46−48^ but to our knowledge this is the first example of such a cation affecting the site-selectivity outcome of a cross-coupling reaction involving a dihalogenated heteroarene. Using a Pd(OAc)_2_/2PPh_3_ catalytic system alongside a KOH (aq.)/*n*-Bu_4_NBr base system similarly boosted C4 selectivity (entries 4 and 5, Table 1). Analogous observations were made employing catalytic **Pd**_**3**_**Cl**_**2**_ (entries 6–9, Table 1). Arguably the best outcome in terms of global **3b**_***C4–Ar***_ product selectivity was obtained using Pd(OAc)_2_/2PPh_3_ or **Pd**_**3**_**Cl**_**2**_ along with KOH/*n*-Bu_4_NBr (entries 3 and 5 respectively). Switching to the longer-chain quaternary ammonium salt *n*-octylammonium bromide (*n*-Oct_4_NBr) in place of *n*-Bu_4_NBr gave the highest product selectivity for **3b**_***C4–Ar***_ (entry 9, Table 1).

**Scheme 4:**
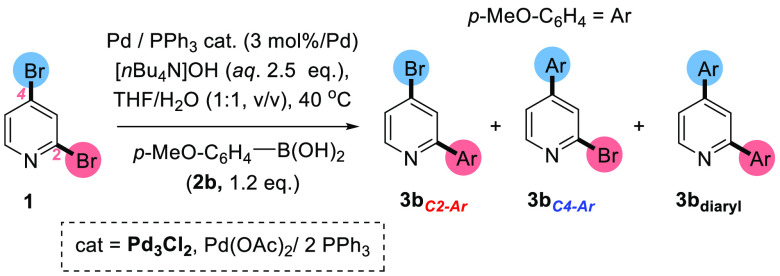
Conditions, Reagents, and Catalysts Used for Kinetic Product Distribution Analysis in SMCC Reactions of **1**

**Figure 2:**
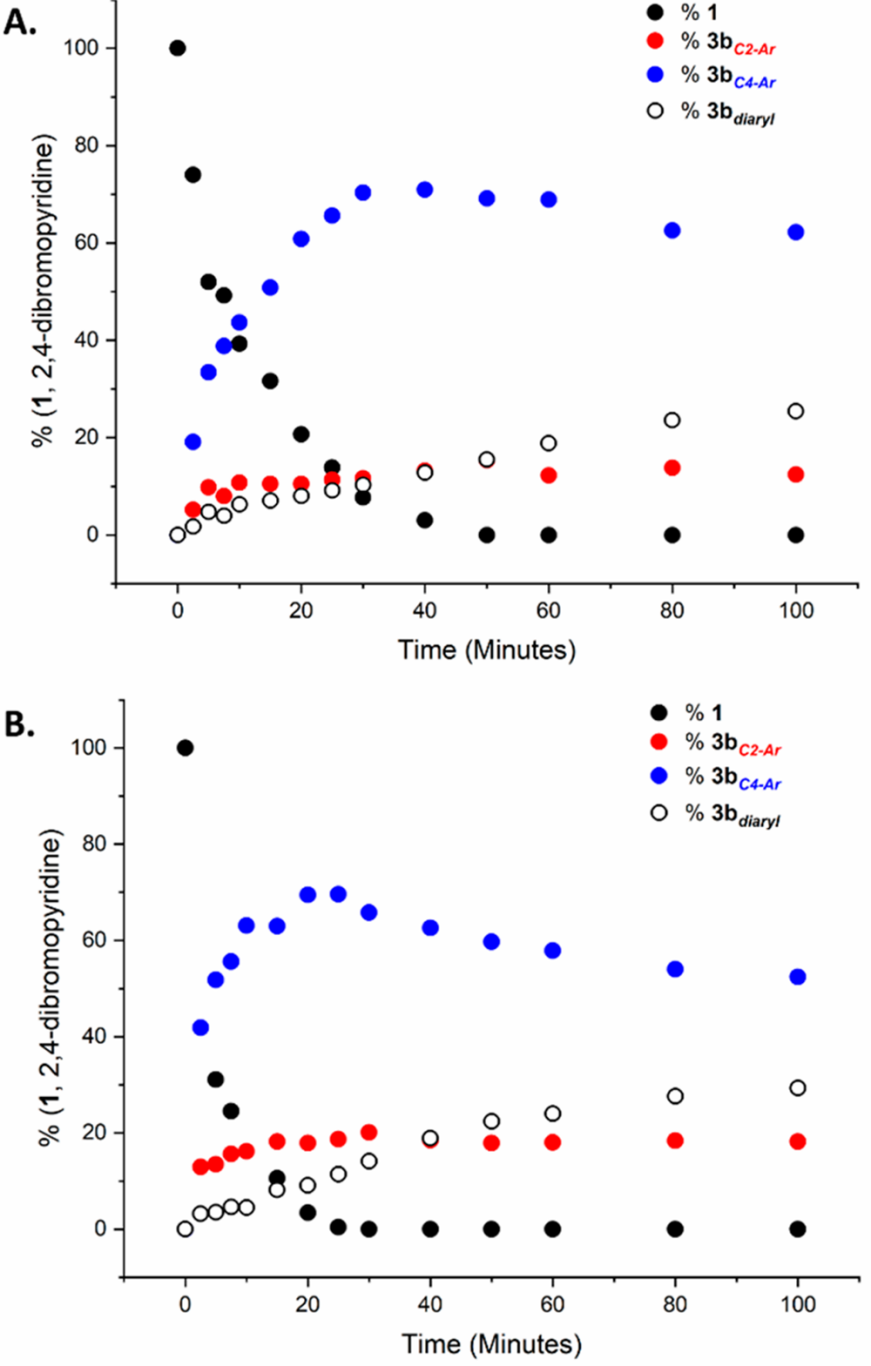
Product distribution of **3b**_***C4–Ar***_, **3b**_***C2–Ar***_, and **3b**_***diaryl***_ as functions of time in the SMCC reaction between **1** and **2b**. Using (A) Pd_3_Cl_2_ and (B) Pd(OAc)_2_/2PPh_3_ as the precatalyst.

An assay was designed to track the product evolution of **3b**_***C4–Ar***_, **3b**_***C2–Ar***_, and **3b**_***diaryl***_ products over time in the SMCC reaction between **1** and *p*-anisylboronic acid **2b**, enabled by the Pd(OAc)_2_/2PPh_3_ and **Pd**_**3**_**Cl**_**2**_ catalyst systems and an *n*-Bu_4_NOH(aq.) base (Scheme 4, Graphs A and B Figure 2) .

Graphs A and B (Figure 2) show that employing **Pd**_**3**_**Cl**_**2**_ or Pd(OAc)_2_/2PPh_3_ as a precatalytic system resulted in broadly comparable overall reactivities with time. In both cases **3b**_***C4–Ar***_ was the predominant product, the quantity of which reached a maximum conversion at approximately 35 and 25 min for **Pd**_**3**_**Cl**_**2**_ and Pd(OAc)_2_/2PPh_3_, respectively. After this time, **3b**_***C4–Ar***_ was slowly converted into **3b**_***diaryl***_ while the amount of **3c**_***C2–Ar***_ remained approximately constant. This study indicated that, with the Pd loading fixed at 3 mol %, the Pd(OAc)_2_/2PPh_3_ catalyst is marginally more efficacious than **Pd**_**3**_**Cl**_**2**_, accounting for the increased **3b**_***diaryl***_ conversion observed with Pd(OAc)_2_/2PPh_3_, compared with **Pd**_**3**_**Cl**_**2**_ in Table 1.

**Scheme 5:**
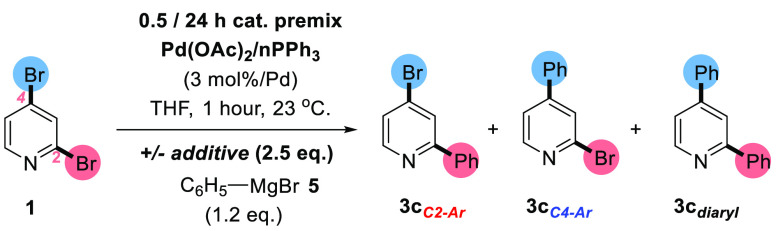
Conditions for the Kumada Cross-Coupling of **1** with Phenylmagnesium Bromide **5** Variables changed are highlighted in bold.

Given our observations on the importance of the Pd/PPh_3_ ratio and aliphatic cation *n*-Bu_4_N^+^ as necessary requirements for atypical **3b**_***C4–Ar***_ site-selectivity in SMCCs, it was decided to assess whether such effects emerge in the Kumada cross-coupling of **1** with phenylmagnesium bromide (**5**) forming **3c**_***C4–Ar***_, **3c**_***C2–Ar***_, and **3c**_***diaryl***_ (Scheme 5).

The Pd(OAc)_2_/*n*PPh_3_ ratio and catalyst prestir time (in THF) were altered, with reactions being run in the presence and absence of *n*-Oct_4_NBr, enabling conversions of **1** and selectivity changes to monoarylated products: **3c**_***C4–Ar***_ and **3c**_***C2–Ar***_ to be fully assessed (Table 2).

**Table 2:** Changes in Conversion of **1** and Product Site-Selectivity Outcomes, upon Changing Reaction Variables in Kumada Cross-Couplings (Scheme 5)

Entry	Pd(OAc)_2_:nPPh_3_	Catalyst prestir time (h)	*n*-Oct_4_NBr	Conv (%)^a^	3c_C2Ar_:3c_C4Ar_:3c_diaryl_^a^
1	No cat.	0.5	–	0	N/A
2	1:4	0.5	–	100	83:0:17
3	1:2	0.5	–	100	91:3:6
4	1:1	0.5	–	99	84:6:9
5	1:1	24	–	85	80:11:8
6	1:1	0.5	+	83	21:68:12
7	1:1	24	+	96	15:77:8
8	1:4	0.5	+	96	67:26:7

a Determined by ^1^H NMR of the crude reaction mixture after 1 h.

In the absence of *n*-Oct_4_NBr, high selectivity for the **3c**_***C2–Ar***_ product was observed (entries 2–4,Table 2). Selectivity for **3c**_***C2–Ar***_ remained, albeit diminishing, when the catalyst prestir time was extended to 24 h (entry 5, Table 2). Employing *n*-Oct_4_NBr instigated a switch in site-selectivity favoring **3c**_***C4–Ar***_ as the major product, thus mirroring the requirement for a quaternary ammonium salt observed for C4-site-selectivity in the SMCC regime *vide supra*.

Lengthening the prestir time to 24 h resulted in a moderate increase in **3c**_***C4–Ar***_:**3c**_***C2–Ar***_ site-selectivity from 3.2:1 to 5.1:1, accompanied by an increase in conversion. The outcome provides an indication that an active and selective Pd catalyst species was generated during this time. The catalyst, generated from Pd(OAc)_2_ and 4PPh_3_, prestirred alongside *n*-Oct_4_NBr (0.5 h, THF), led to **3c**_***C2–Ar***_ product selectivity, confirming the dual requirement of a high Pd:P ratio as well as an additive salt for overall ***3c***_***C4–Ar***_ selectivity under the specified conditions.

**Figure 3:**
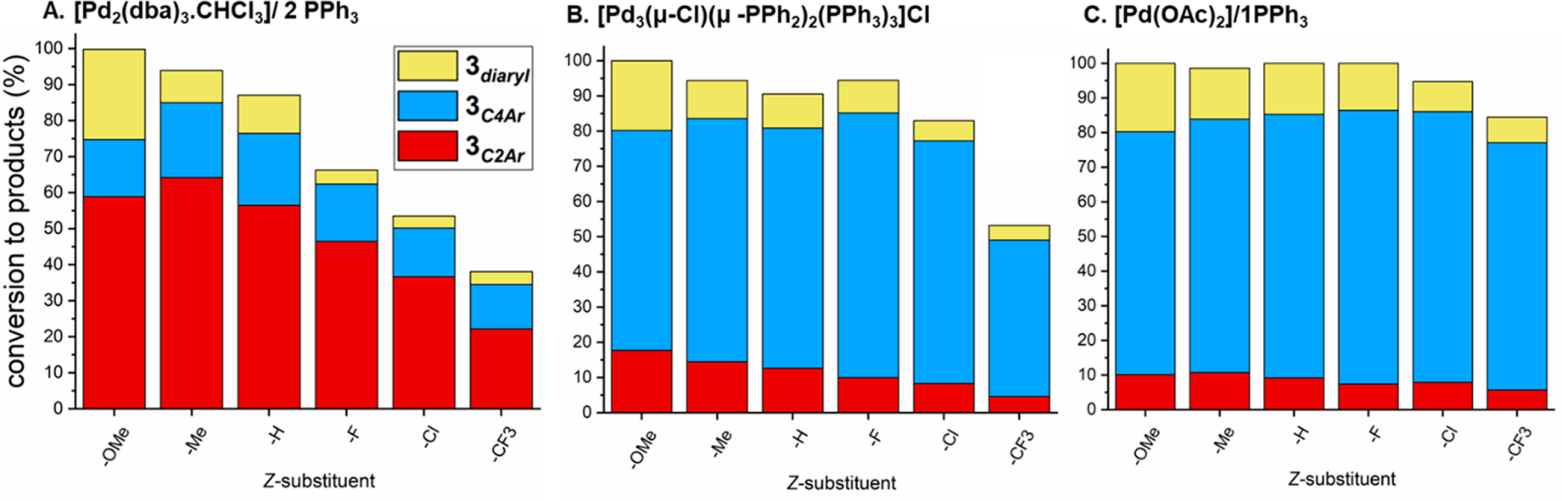
Effect of product selectivities in SMCC reactions as a function of catalyst system employed and *para*-substituent on the phenylboronic acid substrate. (A) Using Pd_2_(dba)_3_·CHCl_3_/2PPh_3_. (B) Pd_3_Cl_2_. (C) Pd(OAc)_2_/1PPh_3_.

**Scheme 6:**
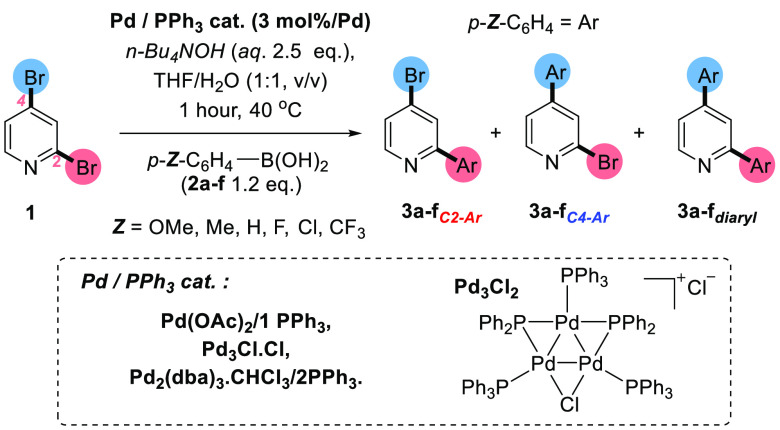
Conditions, Reagents, and Catalysts Used for *para-*Substituent Analysis of Site-Selective SMCC Reactions at **1** Determined by ^1^H NMR analysis of the crude reaction mixture, after 1 h.

To gain insight into the mechanistic dichotomy in site-selectivity seen for **1**, the effect of *para*-aromatic substituents on reaction conversion and site-selectivity was assessed in the SMCC reactions employing appropriate substituted arylboronic acids (Figure 3 and Scheme 6).

The model SMCC reaction was carried out with a series of *para* Z-substituents to determine whether an electronic contribution influenced the overall site-selectivity, in selecting the C_2_–Br or C_4_–Br bonds in **1**. Three different precatalytic systems were employed for this part of the study: first, Pd_2_(dba)_3_·CHCl_3_ (∼93% purity) with 2 PPh_3_, which proved to be an effective **3**_***C2–Ar***_ site-selective catalyst under the conditions. Second, the **3**_***C4–Ar***_ site-selective catalyst systems, namely **Pd**_**3**_**Cl**_**2**_ cluster and Pd(OAc)_2_/1PPh_3_, were assessed (Figure 3).

An important observation from this series of experiments is that the greater the electron-withdrawing capacity of the Z-substituent, the higher the selectivity for the atypical **3**_***C4–Ar***_ product. Concomitant with these observations was lower overall product conversions, indicating that the transmetalation step as rate-determining or that, in some form, the Z-substituent influences reaction site-selectivity involving **1**. Taking the Pd_2_(dba)_3_·CHCl_3_/2PPh_3_ catalyst system (Figure 3A), the most active aryl boronic acid is *para*-anisylboronic acid **2b**, affording high selectivity for **3b**_***C2–Ar***_, although competing **3b**_***diaryl***_ is apparent. Similar behavior was noted for *para*-methyl phenylboronic acid **2d** and phenylboronic acid **2e**.

The response of the **Pd**_**3**_**Cl**_**2**_ cluster catalyst to changes in the *para* Z-substituents of the phenylboronic acids is predictable, in that high selectivity for the **3**_***C4–Ar***_ products were recorded (Figure 3B). A similar but more subtle response is seen for the Pd(OAc)_2_/1PPh_3_ catalyst system (Figure 3C).

**Figure 4:**
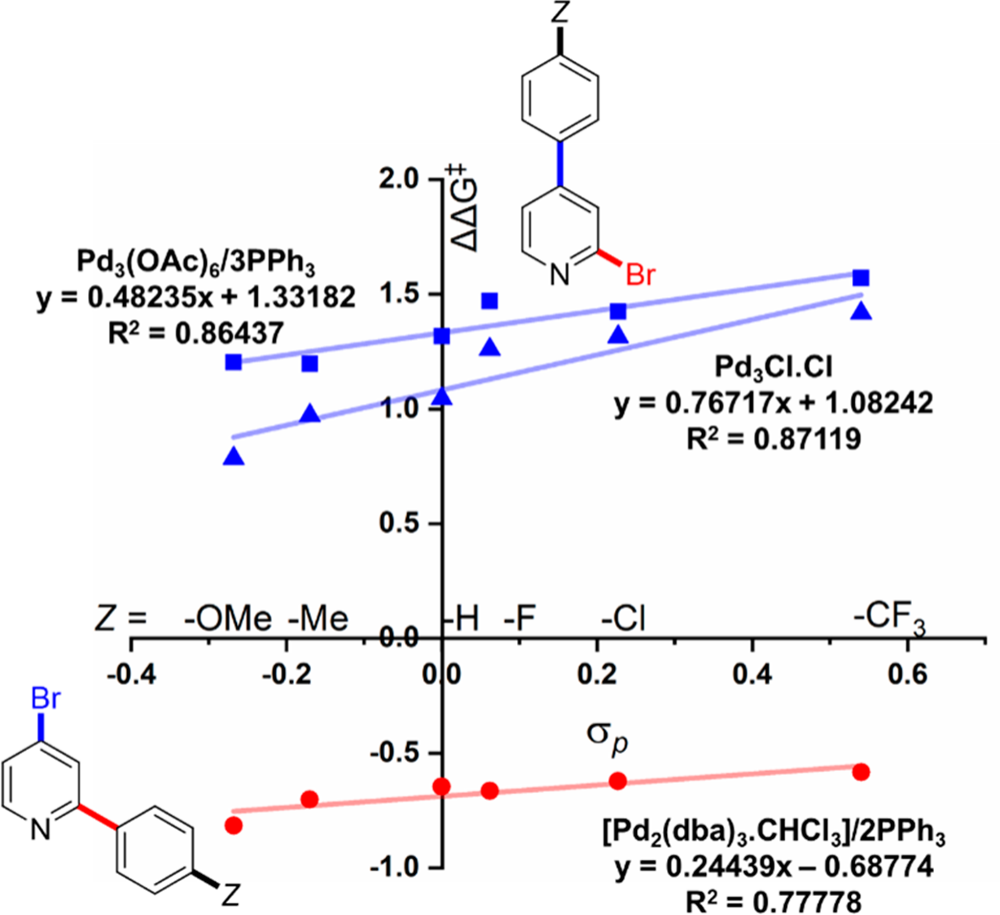
Plot of ΔΔ*G*^‡^ against σ_P_ for *para*-substituent changes in SMCC reactions of **1** with *p*-Z-C_6_H_4_-B(OH)_2_ (**2a**–**f**).

A plot of ΔΔ*G*^‡^ against σ_P_ reveals the reaction sensitivity to the phenylboronic acid *para*-substituent Z (Figure 4). One sees that **Pd**_**3**_**Cl**_**2**_ cluster and Pd(OAc)_2_/1PPh_3_ catalyst systems behave quite differently to Pd_2_(dba)_3_·CHCl_3_/2PPh_3_. The magnitude for the gradient (∼0.24) for the latter catalyst system is in-keeping with the presumption that the aryl boronic acid substituent ought not to affect site-selectivity in **1**, as oxidative addition occurs prior to transmetalation for mononuclear Pd catalysts. However, larger gradients are seen for the **Pd**_**3**_**Cl**_**2**_ cluster (∼0.77) and Pd(OAc)_2_/1PPh_3_ (∼0.48), providing evidence that these catalyst systems behave in a similar manner.

**Scheme 7:**
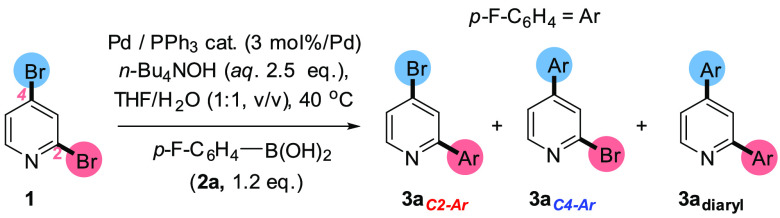
Conditions and Reagents Used for Determining the Effects of a Variety of P-Ligands on Site-Selective SMCC Reactions at **1**

**Figure 5:**
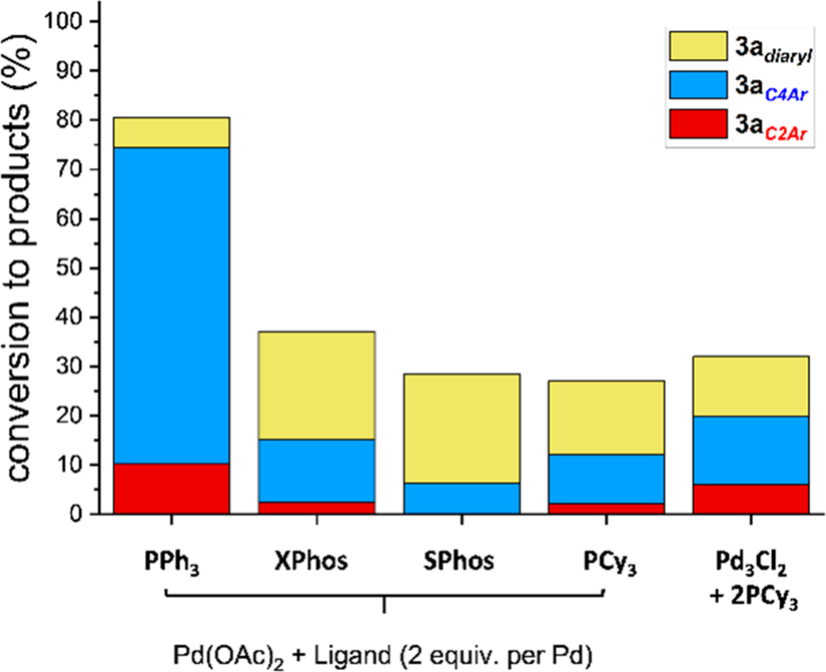
Performance of phosphorus-containing Pd precatalysts systems in site-selective Suzuki–Miyaura cross-coupling of **1**.

Given the response of the SMCC reactions of 2,4-dibromopyridine **1** toward the ubiquitous ligand PPh_3_, we decided to screen other widely used phosphorus-containing ligands (Scheme 7, Figure 5). We tested catalyst mixtures with Pd(OAc)_2_/ligand ratios of 1:2 in the reaction of **1** with phenylboronic acid **2c** to give products **3c**_***C2–Br***_, **3c**_***C4–Br***_, and **3c**_***diaryl***_. Based on consumption of **1** we see low conversions to the monoarylated products, albeit with a bias toward **3c**_***C4–Ar***_. However, the dominant product is **3c**_***diaryl***_ resulting from diarylation.

## Post-rationalization and Further Analysis

**Figure 6:**
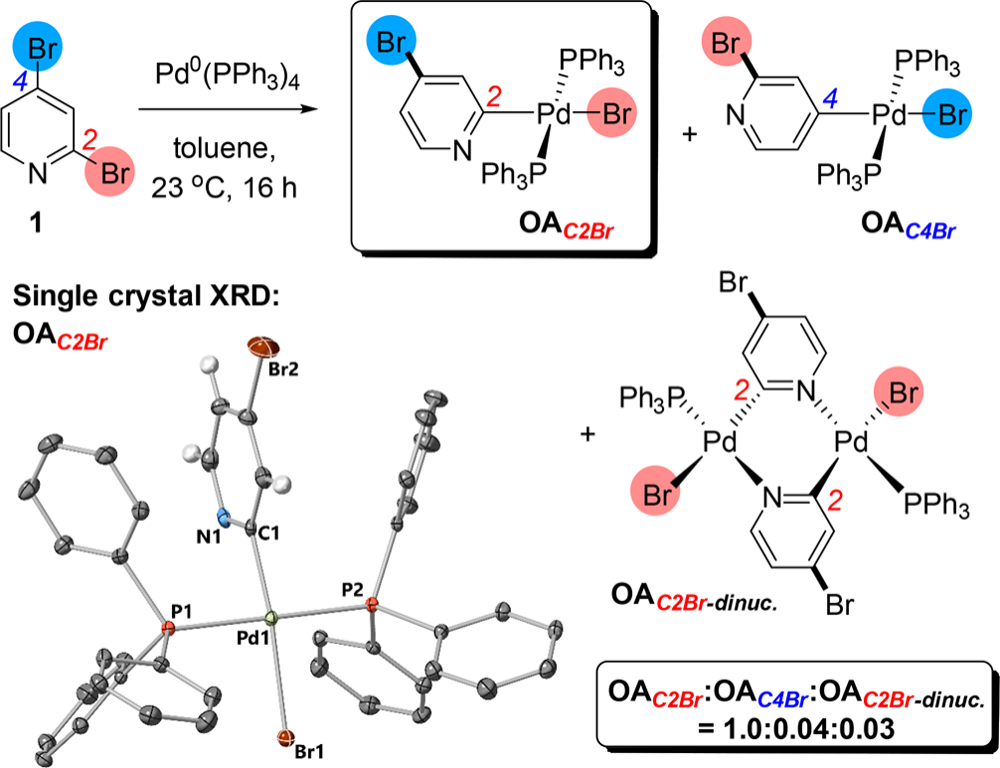
Confirmation of mechanistic reasoning for C_2_–Br site-selectivity in the reaction of Pd^0^(PPh_3_)_4_ with **1** at 23 °C.

The important take-home message from the examples presented thus far is that a switch in site-selectivity for the **3**_***C4–Ar***_ product in SMCC and Kumada cross-coupling reactions occurs when a quaternary ammonium salt *n*-R_4_NX (R = butyl or octyl, X = Br^–^ or HO^–^) is employed alongside a low catalytic equivalence of *n*PPh_3_ per Pd(OAc)_2_ (where 0.5 < *n* ≥ 2.5 in the case of the SMCC reaction). The results point to the existence of different mechanisms being available to Pd, as the Pd(OAc)_2_/*n*PPh_3_ ratios are changed; i.e., the Pd catalyst speciation is different, which is in-keeping with our earlier studies.^32^ The higher C2 site-selectivity for **3**_***C2–Ar***_ using higher equivalences of PPh_3_ relative to Pd mirrors that reported by Cid et al.^7^ using a Pd^0^(PPh_3_)_4_ catalyst which is closely related to the [Pd^0^(PPh_3_)_*n*_(OAc)]^−^ active species that arises from Pd(OAc)_2_/≥3 PPh_3_.^32,42−45^ Indeed, in our study, in line with observations by Cid et al.,^7^ we found that the direct reaction of **1** with Pd^0^(PPh_3_)_4_ in toluene at 23 °C (Figure 6) gave the C2-oxidative addition product **OA**_***C2–Br***_ as the major regioisomer (**OA**_***C2–Br***_/**OA**_***C4–Br***_ ≈ 25:1 by ^31^P NMR spectral analysis of a crude reaction mixture).

The major regioisomer **OA**_***C2–Br***_ was characterized by X-ray diffraction analysis (corroborated by NMR spectroscopic analysis of the single crystal analyzed by X-ray diffraction). Cid et al. characterized the dinuclear Pd complex, **OA**_***C2–Br-dinuc***_ (Figure 6), resulting from loss of PPh_3_ from **OA**_***C2–Br***_ and subsequent dimerization of the putative 14-electron Pd^II^ species. These results indicate that oxidative addition of Pd^0^(PPh_3_)_*n*_ (*n* = 2 or 3) is the starting point for the SMCC of **1** when Pd^0^(PPh_3_)_4_ or Pd(OAc)_2_/≥3PPh_3_ is used as the precatalyst system, accounting for the overall C2 site-selectivity observed in our study, in addition to the previously reported cross-coupling reactions involving **1**.^7,8^

**Figure 7:**
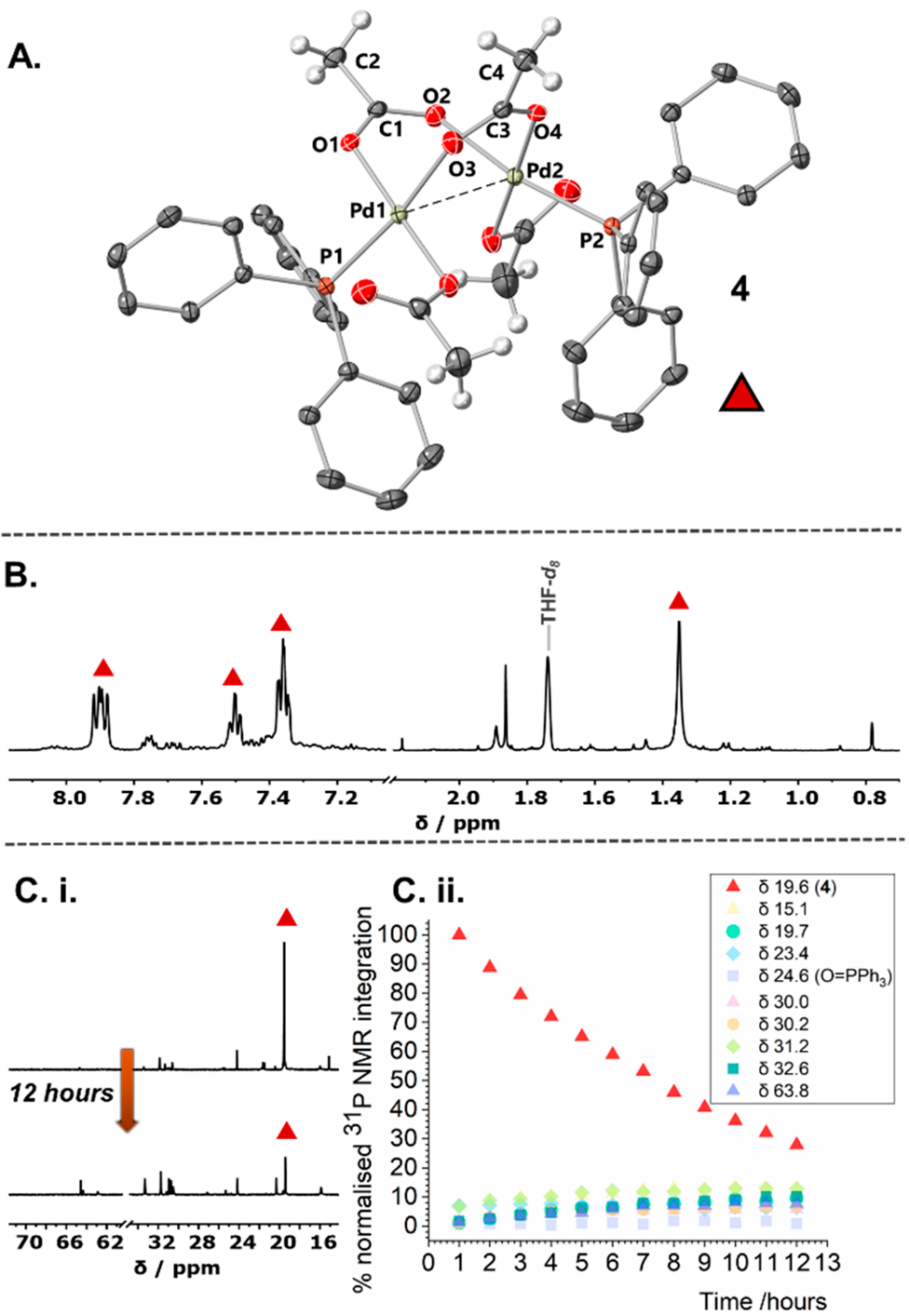
Analysis of the THF-*d*_*8*_. solution arising from the mixture of Pd(OAc)_2_/1PPh_3_. (A) XRD structure of a single crystal of **4** is shown (selected atoms). (B) ^1^H NMR analysis, confirming solution presence of **4** ca. 10 min after mixing at 25 °C. (C. i. and ii.) ^31^P NMR spectral data and reaction speciation, showing the decay of **4** and growth of multiple P-containing species over 12 h, 25 °C.

Further experiments however showed that by stirring a solution of Pd(OAc)_2_ and 1 PPh_3_ at 0 °C for 5 min, layering of the solution with hexane and subsequent storage at −18 °C led to the growth of reddish-brown crystals. These have been confirmed by single crystal X-ray diffraction analysis to be the dinuclear Pd^II^ complex [Pd^II^(μ_2_-OAc)(κ-OAc)(PPh_3_)]_2_ (**4**), containing bridging and terminal acetate groups, with one terminal PPh_3_ ligand at each Pd center (Figure 7A). The structure of **4** was also confirmed by ^1^H NMR spectroscopic analysis to be the major solution species formed immediately after mixing Pd(OAc)_2_ and 1 PPh_3_ (diagnostic peaks for the acetoxy methyl group at δ_H_ 1.34 ppm) (Figure 7B). We did not see any evidence of low-ligated phosphine adducts of Pd_3_(OAc)_6_.^49,50^ The broadness of the single methyl resonance (for the OAc ligands) suggests that the two acetate environments are in exchange at 25 °C, supported by the proximal relationship as indicated in the solid-state.

Complex **4** was originally reported by Wilkinson et al., who described it as unstable in the solid-state^51^—we concur with this description but were fortunate in being successful in obtaining a solid-state structure. Interestingly, the reactivity of **4** under (hydrogenative) reducing conditions has been investigated.^52,53^

**Scheme 8:**
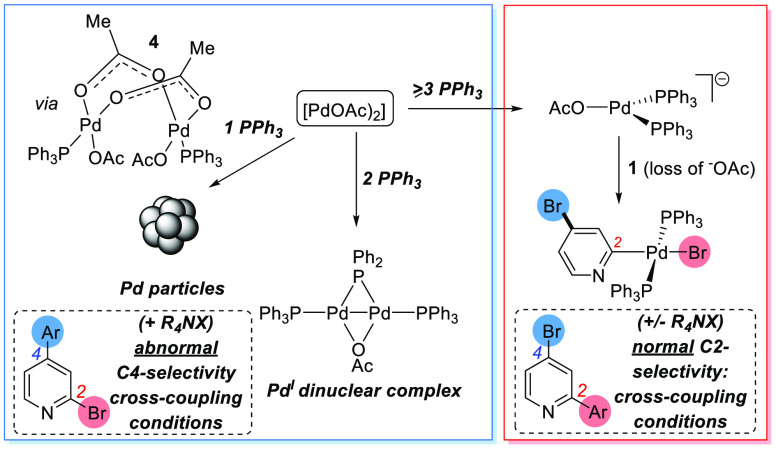
Dichotomy in Site-Selectivity at **1**: Different Pd Species Arising from Different Ratios of Pd(OAc)_2_/*n*PPh_3_ Result in Different Cross-Coupling Selectivities under Cross-Coupling Conditions

We further investigated the solution behavior of **4** in dry *d*_8_-THF at room temperature by ^31^P NMR spectroscopic analysis (Figure 7C). Over time, a darkening of the solution was noted concomitant with the formation of multiple different phosphorus-containing species (Figure 7C. ii.). While Pd(OAc)_2_/2 or ≥3PPh_3_ is known to reduce/activate at the expense of concomitant oxidation of PPh_3_*via. trans*-[Pd(OAc)_2_(PPh_3_)_2_], in this case, O=PPh_3_ was only observed as a minor biproduct of the process. ^1^H NMR spectroscopic analysis of the post reaction solution indicated that Ac_2_O formed as a major byproduct, alongside AcOH, in a 1:3 ratio, respectively. This observation points toward **4** facilitating a different mechanism for activation of Pd(OAc)_2_ in the presence of 1 equiv of PPh_3_, when compared with *trans*-[Pd(OAc)_2_(PPh_3_)_2_].^32,42−45^ TEM analysis of the decomposed solution of **4** (after overnight reaction at room temperature) demonstrated the presence of large, spherical Pd particles (micron-sized).^32^ When Pd(OAc)_2_ was similarly treated with 2PPh_3_ at room temperature, a dinuclear Pd^I^ species was found to be transiently stable in THF. The observation that optimal catalyst activity and selectivity occur when relatively low precatalytic ratios of PPh_3_ to Pd(OAc)_2_ are employed, i.e. enabling formation of aggregated Pd clusters and particles, strongly correlates with the observed reactivity and selectivity involving cross-coupling reactions of **1**, in keeping with differences in reactivity seen for the related 2-bromopyridine substrate.^32^Scheme 8 summarizes our overall findings, linking catalyst speciation under differing Pd(OAc)_2_/*n*PPh_3_ regimes.

In addition to the Pd/P ratio, a key requirement for high **3**_***C4–Ar***_ selectivity is the presence of a quaternary ammonium salt R_4_NX (R = *n*-butyl or *n*-octyl, X = Br^–^, OH^–^). The latter requires further comment and experimental corroboration, as there is a wealth of literature that explores the stabilization of highly active anionic PdNP catalyst species by salts. Dupont et al. reported that catalytic Pd particles, generated by *in situ* reductive activation of a palladacyclic compound, could be stabilized by imidazolium salts for applications in Heck coupling.^54^ The immediate electropositive outer layer of a metal nanoparticle can be stabilized by anions, the sterics and basicity of which influence PdNP stability.^55,56^ In a regime analogous to the electrical double layer, the anionic layer can in turn be stabilized by a layer of cations. Astruc et al. explored this *electrosteric stabilization* in the design of bespoke architectures for the stabilization of PdNPs.^57,58^ This valuable prior knowledge underpins our hypothesis that *electrosteric stabilization* of PdNPs is critical to the site-selectivity switch seen in the cross-coupling reactions of **1**. Thus, stabilized PdNPs, formed *in situ* from either precatalysts Pd(OAc)_2_ and 1PPh_3_ or **Pd**_**3**_**Cl**_**2**_ by additive or *in situ* generated salts, are the catalyst species responsible for this atypical selectivity and relatively high activity, compared to that of the dominant mononuclear catalytic species generated from Pd(OAc)_2_ and ≥3PPh_3_, Pd(PPh_3_)_4_, or Pd_2_(dba)_3_·CHCl_3_/2PPh_3_.

We have tested our hypothesis further and shown that a tris-imidazolium tribromide salt can effectively stabilize PdNPs enabling a marked rise in site-selectivity at **1**, exhibiting a **3b**_***C4–Ar***_:**3b**_***C2–Ar***_ ratio of 17.6:1, with a relatively low formation of **3b**_***diaryl***_ product (see Supporting Information (SI) for further details).

The notion that changes to Pd catalyst speciation might result in different chemoselectivities has been reported by Schoenebeck et al., elaborating on earlier findings by Fu et al.^14,16,59^ They rationalized that cross-coupling selectivities at 4-chlorophenyl triflate occurred at the C–Cl site in reactions catalyzed by [Pd^0^(L)_1_] and the C–OTf side in reactions catalyzed by the analogous [Pd^0^(X)(L)]^−^ complex (where X = an anion present in the system, L = P*t*Bu_3_). In this case, however, both active catalysts were proposed to be mononuclear Pd^0^ ligated species (based on experimental and computational evidence). Indeed, subsequent work used 4-chlorophenyl triflate as a probe to differentiate between mechanisms arising from a dinuclear Pd^I^ precatalyst.^60^*Our work* has similarly shown two different mechanisms for the activation of different sites of the dibrominated heterocycle **1**. However, in the case of selectivity for the C4 position, under the reaction conditions that we have identified, it is highly unlikely that such mononuclear Pd^0^ species can be present, an assertion based on what is known about Pd speciation as the Pd(OAc)_2_/nPPh_3_ ratio is altered (*vide supra*).^32^

Finally, the synthetic utility of the Pd_3_(OAc)_6_/3PPh_3_ catalytic system was demonstrated in the synthesis of a novel 2,4-disubstituted pyridine by successive C4-selective arylation by an SMCC reaction at **1**, followed by an Ullman etherification^61^ at its C2-position (see SI for further details).

## Mechanistic Hypotheses

**Scheme 9:**
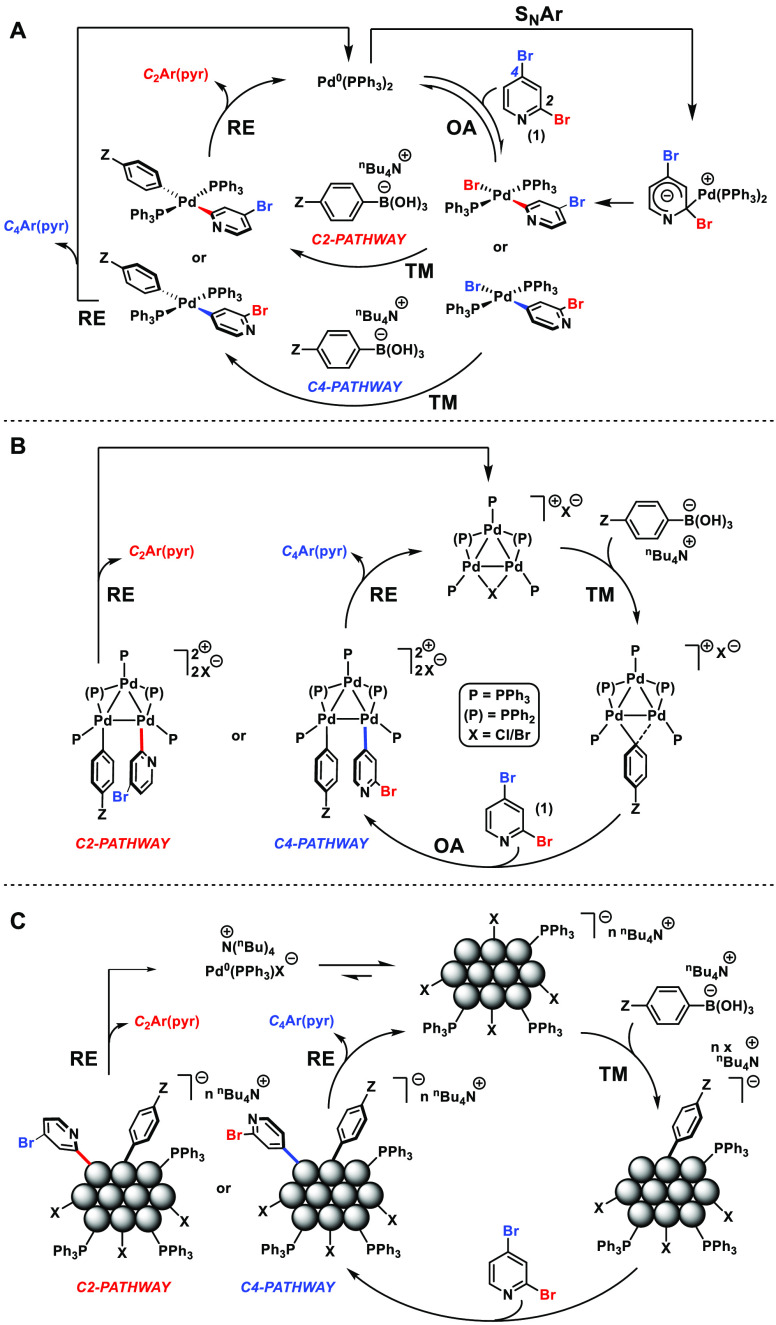
Mechanistic Hypotheses: (A) Catalytic Cycle Involving Mononuclear Pd Species, *via* Classical Pd Intermediates or Alternative Route Involving a S_N_Ar-Type Mechanism; (B) Catalyst Cycle Based on That Evidenced by Li et al.^29^ Involving Pd_3_ Cluster Species; (C) Proposed Involvement of Higher Order Pd Agglomerates (Note: Only Details of Key Steps Are Shown – *trans*–*cis* Isomerizations and Ligand Dissociation/Association Are Involved) Note: (P) = PPh_2_; P = PPh_3_; X = anion, e.g. Br or OH.

Given that such profound site-selectivity changes are seen for cross-couplings of 2,4-dibromopyridine **1**, on changing Pd catalyst speciation in the presence of stabilizing salt additives, a discussion concerning the mechanistic implications is pertinent. If one assumes that only mononuclear Pd species are the relevant catalyst species (dependent on reaction conditions), then selection of the C_2_–Br over C_4_–Br bond occurs on activation of **1** by a Pd^0^(PPh_3_)_*n*_ complex (where that *n* is typically ≥2).^7^ A neutral pathway is depicted in Scheme 9A. Here, the relative rates of oxidative addition would explain the typical site-selectivity for C_2_–Br, presuming this step is irreversible and that the associated higher intrinsic electrophilicity of this bond lowers the barrier to its activation. The case for this catalytic cycle has been made strongly elsewhere;^7^ however, oxidative addition must be reversible (in Scheme 9A) in order to account for our experimental observations. Switching site-selectivity from C_2_–Br to C_4_–Br, i.e. **3**_***C4–Ar***_ over **3**_***C2–Ar***_, arguably requires a quite different ligand environment,^14,59,60,62^ or a complete change in mechanism. We have not shown anionic mononuclear Pd species here, but clearly in the presence of *n*-R_4_NBr, such a pathway could be operative, with *n*-R_4_N^+^ acting as the stabilizing cation.^43,45,63^

Maes and Jutand et al.^64^ have reported strong evidence for the existence of an S_N_Ar mechanism for the activation of 5-substituted-2-bromo-pyridines, which is therefore shown in **A** for the C2-arylation pathway, important given the structural similarity to **1**.

An alternative mechanism based on the strong experimental support reported by Li et al. “Pd_3_ cluster” catalysis is shown in Scheme 9B.^29^ In this case the Pd_3_Cl_2_ cluster catalyst, via formation of a Pd_3_-hydroxo species, was proposed to activate the organoboronic acid first, the adduct of which could then activate the aryl halide. Inversion of the oxidative addition/transmetalation steps could explain the higher than expected Z-substituent sensitivity in the site-selective SMCC reaction involving **1**, particularly in the region where Pd_3_ clusters/Pd nanoparticles are catalytically competent (Figure 3 and Figure 4).

A third scenario (Scheme 9C) highlights the potential role of Pd nanoparticles (agglomerates) in the activation of **1**, in essence like the mechanism depicted in Scheme 9B. The Pd nanoparticles are shown ligated by PPh_3_ and halide ligands, as it is established that such stabilizing surface interactions are important.^65,66^ In this case, an aryl boronate complex could be activated by the Pd nanoparticle surface, prior to oxidative addition of the C_4_–Br bond of **1**. The interaction of base and anionic aryl boron species at Pd nanoparticle surfaces has been proposed by El-Sayed et al.^67^ Such a situation aligns with the Z-substituent effect (aryl boronic acid) on reaction efficacy and site-selectivity. The scenario also fits with the observed speciation arising from Pd(OAc)_2_/1PPh_3_*vide supra*—the optimized catalyst system. There can be no doubt that the mechanistic complexity presented in Scheme 9 requires significant independent investigation. (We have embarked on computational studies (DFT) to support the mechanistic hypotheses described in Scheme 9. However, we are yet to obtain reasonable results, as the conformational flexibility in these large Pd_3_Cl_2_ cluster species, and related downstream intermediates, is high, leading to local energy minima. We selected to not simplify the Pd_3_ structural models, as the ligand microenvironment surrounding these is clearly important in stabilization and in controlling how substrates approach the Pd centers and their activation.) We anticipate that specialist experimental methods (real-time fluorescence^26^ and X-ray absorption spectroscopy^24,25^) might reveal insight into the underlying catalyst speciation behavior and complexity.

## Conclusions

In conclusion, our studies have shown that site-selective cross-couplings of 2,4-dibromopyridine **1** are affected by the type of catalyst system used and catalyst speciation that ultimately results under working reaction conditions. The observations are clear for both SMCC and Kumada cross-coupling reactions. We have confirmed that Pd(OAc)_2_/≥3PPh_3_, and related catalyst systems, enable typical C2-selctivity. However, for the Pd(OAc)_2_/≤2PPh_3_ catalytic system, atypical C4-selectivity is seen, an outcome that is mirrored using the **Pd**_**3**_**Cl**_**2**_ cluster catalyst. The addition of a quaternary ammonium salt proved to be a critical additive for atypical C4-selectivity, supporting the hypothesis that high site-selectivity is attributable to PdNPs formed *in situ*, for which the quaternary ammonium salt plays a stabilizing role. The hypothesis was supported using a bespoke tris-imidazolium tribromide salt, capable of stabilizing Pd nanoparticles.^54,55,57^ Addition of such a salt to the SMCC reaction system led to a significant increase in the C4-selectivity. Our findings mark the first examples of site control of a dihalogenated heteroarene, switching between two halogens of the same type, while using the same Pd source [Pd_3_(OAc)_6_] and the same ligand type PPh_3_. It underlines the importance of controlling precise metal–ligand ratios for optimal catalyst performance. Interestingly, in the context of site-selective SMCCs, Spivey et al.^4^ stated that “*...caution must be applied when trying to rationalise switches in site-selectivities as a function of changes of conditions as the observed products may not arise from the ligated species expected*.” We can now confirm that is the case, but that reaction outcomes can be controlled through understanding fundamental changes in Pd catalyst speciation.

More generally our study has demonstrated that the activity of well-established Pd catalyst mixtures can be very easily altered by small changes to the reaction conditions. We can recognize that understanding and controlling catalytic speciation may allow simple Pd catalytic precursors and simple inexpensive ligands (e.g., PPh_3_) to exhibit unique properties in catalytic cross-coupling chemistries. Such an approach could be potentially exploited to avoid the use of expensive ligand architectures. Furthermore, our approach to understanding the Pd catalyst speciation may serve to complement understanding in other powerful site-selective cross-couplings.^12,13,68−71^

## Supporting Information Available

Full procedures, compound characterization data, catalysis studies, and X-ray details (PDF)

## Accession Codes

CCDC 2060853–2060856 contain the supplementary crystallographic data for this paper. These data can be obtained free of charge via www.ccdc.cam.ac.uk/data_request/cif, or by emailing data_request@ccdc.cam.ac.uk, or by contacting The Cambridge Crystallographic Data Centre, 12 Union Road, Cambridge CB2 1EZ, UK; fax: +44 1223 336033.
