# Supplementary material for: A Dichotomy in Cross-Coupling Site Selectivity in a Dihalogenated Heteroarene: Influence of Mononuclear Pd, Pd Clusters, and Pd Nanoparticles—the Case for Exploiting Pd Catalyst Speciation
Source: J Am Chem Soc. 2021 Jun 21;143(25):9682–93. doi: 10.1021/jacs.1c05294 (PMC8297865; doi:10.1021/jacs.1c05294)
Supplement: Supplementary file 1 — ja1c05294_si_001.pdf [file ja1c05294_si_001.pdf]

Supporting Information for

A dichotomy in cross-coupling site-selectivity in a dihalogenated heteroarene: Influence of mononuclear Pd, Pd clusters and Pd nanoparticles – the case for exploiting Pd catalyst speciation

Neil W. J. Scott[†], Mark J. Ford[‡], Neda Jeddi[†], Anthony Eyles[†], Lauriane Simon[†], Adrian C. Whitwood[†], Theo Tanner[†], Charlotte E. Willans[ǂ] and Ian J. S. Fairlamb*[†]

[†] Department of Chemistry, University of York, Heslington, York, YO10 5DD, U.K.

* ian.fairlamb@york.ac.uk

[‡] Bayer AG, Alfred-Nobel-Strasse 50, 40789 Monheim, Germany.

[ǂ]School of Chemistry, University of Leeds, Leeds, LS2 9JT, U.K.

__Table of Contents__

1.0. General Information ____ 2

1.1. Compound Preparatory Techniques and Methods for In-laboratory Analysis ____ 2

2. Experimental Details ____ 4

2.1. General Procedure: $Pd_3(OAc)_6$/nPPh_3 Ratio Experiments (Figure 1 – Main Paper) ____ 4

2.2. Base and Additive Effects – SMCC Reactions (Table 1 – Main Paper) ____ 5

2.3. Product Evolution Curves for Site-selective SMCC Reactions at 2,4-Dibromopyridine 30 (Figure 2 – Main Paper) ____ 6

2.4. Site-selectivity in Kumada-Corriu Cross-couplings at 1 (Table 2 – Main Paper) ____ 7

2.5. Para-substituent Effects on Site-selectivity in SMCC Reactions at 1 (Figures 3 & 4 – Main Paper) ____ 9

2.6. General Workup Procedure for SMCC Reactions ____ 10

2.7. ^{1}H NMR Analysis of NMR Samples of Cross-coupling Reactions ____ 10

2.8. Oxidative Addition Reaction of [$Pd^0(PPh_3)_4$] to 1 (Figure 6 – Main Paper) ____ 12

2.9. Reaction of $Pd_3(OAc)_6$ with 3 Equivalents of PPh_3 (Figure 7 – Main Paper) ____ 15

3. Characterisation of Organic Products ____ 18

3.1. 2,4-Disubstituted Pyridines: Suzuki-Miyaura/Kumada Cross-coupling Reactions ____ 18

3.2. Synthesis of Tris-imidazolium Tribromide (6) ____ 36

3.3. Synthetic Utility Employing a C4-Site-Selective SMCC Reaction First, Followed by an Ullmann Coupling ____ 40

4. X-Ray Crystallography ____ 42

5. NMR Spectral Data for Isolated Organic Compounds ____ 49

6. References ____ 70

1.0. General Information

1.1. Compound Preparatory Techniques and Methods for In-laboratory Analysis

Reagents were purchased from Merck (Sigma-Aldrich), Alfa Aesar, Acros Organics or Fluorochem and used as received unless otherwise stated. $Pd(OAc)_2$ (indicated as >99% purity based on Pd; nitrite-free as judged by IR and NMR spectroscopic analysis) was obtained from Precious Metals Online (PMO) and used as received. Triphenylphosphine (PPh_3) was purified by recrystallization from hot ethanol and dried under high vacuum, before being stored over P_2O_5 for *ca.* 7 days. THF was dried by refluxing over finely-sliced sodium metal (2 × 8 hours) before being distilled, transferred to an ampoule *via* cannula and subsequently deoxygenated by bubbling with argon for *ca.* 30 min. THF-d_8 and CD_2Cl_2 were freeze-pump-thaw degassed before being distilled into an ampoule and stored under Ar. Petroleum ether (petrol/PET) refers to the fraction of petroleum that distils at 40-60 °C. Brine refers to a saturated, aqueous solution of NaCl.

2,4-Dibromopyridine **1** was purchased from Fluorochem and used as received (special note: significant bromide impurities in material purchased from other suppliers were found, likely to be bromide salts, thus the purity was checked by ^{1}H NMR using trimethoxylbenzene as an external standard; typical purity was >99%). Aryl boronic acids were purchased from Tokyo Chemical Industries (TCI) or Fluorochem and used as received. *N*-tetrabutyl ammonium hydroxide (n-Bu_4NOH {aq.}) was purchased (Merck) as a 1.5 M aqueous solution, diluted to 1.0 M using deionised water before being transferred into an ampoule and deoxygenated by bubbling through with N_2 on a Schlenk line. KOH (1.0 M, aq.) was prepared in-house using deionised H_2O and deoxygenated by bubbling through with N_2 and stored in a J. Youngs tap-sealed ampoule. Phenylmagnesium bromide (**5**) solution (1.2 mL, 1.0 M in THF) was purchased from Merck. After receipt, the solution was transferred *via* cannula, driven by N_2 balloon pressure, into an oven-dried ampoule, sealed with a J. Youngs tap. Tetra-*n*-butylammonium bromide (n-Bu_4NBr) and tetra-*n*-octylammonium bromide (n-$octyl_4NBr$) were purchased from Merck and used as received, taking care to minimise exposure to air, due to their hygroscopicity.

$[Pd_3(\mu\text{-}Cl)(\mu\ PPh_2)_2(PPh_3)_3]Cl$ (abbreviated to $\mathbf{Pd_3Cl_2}$ in the paper) was prepared according to an adapted literature procedure.[1] $[Pd^0(PPh_3)_4]$ was prepared using an established literature procedure, published by D. R. Coulson and stored in an Argon-filled glovebox at -30 °C.[2]

All reactions were carried out using an Ar-filled glovebox or by Schlenk techniques (high vacuum, liquid nitrogen trap on a standard in-house built dual line), to eliminate atmospheric air or adventitious moisture from the reaction systems. Room temperature upper and lower limits are stated as 13-25 °C, but typically 21 °C was recorded. Thin layer chromatography (TLC) was carried out using Merck 5554 aluminium–backed silica plates (silica gel 60 F254) and spots were visualized using UV light (at 254 nm). Retention factors (R_f) are reported in parentheses along with the solvent system used. Flash column chromatography was performed using Sigma-Aldrich 60 Å silica gel (SiO_2, particle size 40–63 µm) and a solvent system as reported in the text.

1.2 Instrument Details and Methods for Compound Characterisation

NMR spectra were obtained in the solvent indicated in the text below, using a Bruker AVIIIHD 500 instrument (500 MHz [^{1}H], 470 MHz [^{19}F], 203 MHz [^{31}P] 125 MHz [^{13}C]) or JEOL ECX400 or JEOL ECS400 spectrometer (400 MHz [^{1}H], 101 MHz [^{13}C] and 377 MHz [^{19}F]). Chemical shifts (δ) are reported in parts per million (ppm) and were referenced to the residual non-deuterated solvent of the deuterated solvent used; $C\mathit{H}Cl_3$: δ ^{1}H = 7.26 and ^{13}C = 77.16 ($\mathit{C}DCl_3$), CD_2Cl_2 : ^{1}H = 5.31 ($CD\mathit{H}Cl_2$) and ^{13}C = 54.0, THF-d_8 δ ^{1}H = 3.59 ($OC\mathit{H}_2CH_2$), ^{13}C = 67.57 $O\mathit{C}H_2CH_2$), ^{1}H = 1.73 ($OCH_2C\mathit{H}_2$) ^{13}C = 25.37 ($OCH_2\mathit{C}H_2$). Spectral data was typically collected at 298 K (25 °C).

^{31}P NMR spectral data were collected with proton decoupling, unless otherwise stated. ^{31}P NMR spectra were typically recorded using 128 scans and a spectral window of 300 ppm (δ 250 to -50 ppm). Chemical shifts for ^{31}P resonances were calibrated by externally referencing to an 85% H_3PO_4 in H_2O (w/w). This was practically carried out by inserting a sealed, vacuum-dried capillary tube containing 85% H_3PO_4 in H_2O (w/w) into an NMR tube containing the sample of interest, collecting a ^{31}P NMR spectrum and setting the $H_3\mathit{P}O_4$ resonance to 0 ppm. All ^{31}P and ^{13}C NMR spectra were obtained with ^{1}H decoupling. All NMR spectra were processed using MestReNova (MNova) software (using versions 12–14).

HRMS ESI-MS spectra were measured using a Bruker Daltronics micrOTOF MS, Agilent series 1200LC with electrospray ionisation (ESI) or on a Thermo LCQ using electrospray ionisation, with <5 ppm error recorded for all HRMS samples. LIFDI mass spectrometry was carried out using an JEOL AccuTOF GCx-plus instrument (JMS-T200GC), fitted with a probe produced by Linden CMS. The probe was equipped with 13 µm emitters on an AccuTOF. Alternatively, LIFDI-MS was carried out using a Waters GCT Premier MS Agilent 7890A GC instrument. Mass to charge ratios (*m/z*) are reported in Daltons. High resolution mass spectra (HRMS) are reported with <5 ppm error (ESI and LIFDI). For clarity, LIFDI data are reported for ^{106}Pd, the most abundant natural isotope of Pd, which is part of 'exact mass' values.

Infrared spectra were obtained using a Bruker ALPHA-Platinum FTIR Spectrometer with a platinum-diamond ATR sampling module.

Melting points were recorded using a Stuart digital SMP3 melting point analysis machine.

Elemental analysis (Carbon, Hydrogen and Nitrogen {CHN} content) was carried out on an Exeter Analytical Inc. CE-440 analyser.

For single crystal X-ray crystallographic analysis details, see Section 4.

2. Experimental Details

2.1. General Procedure: $Pd_3(OAc)_6/nPPh_3$ Ratio Experiments (Figure 1 – Main Paper)

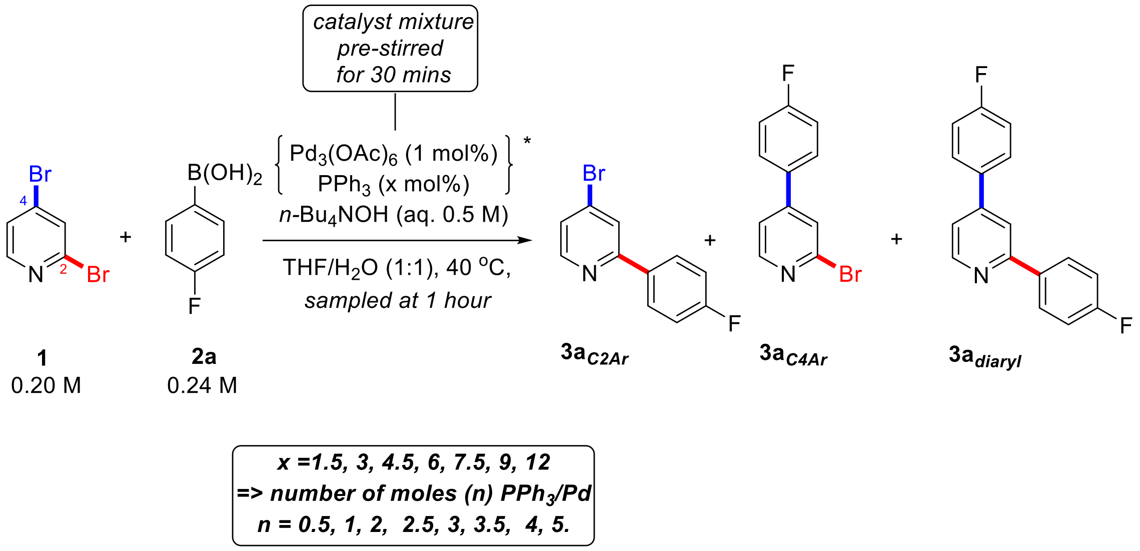

Scheme S1 Summarising the conditions and reagents used for the SMCC between 2,4-dibromopyridine 1 and 4-fluorobenzeneboronic acid 2a. Conversions and site-selectivities were determined by analysing the ^{1}H NMR spectrum of a sample taken after 1 hour.

An oven-dried Schlenk tube (Flask 1) charged with $Pd_3(OAc)_6$ (6.7 mg, 0.03 mmol {3 mol%/Pd}) and triphenylphosphine (Table S1) was evacuated and backfilled with N_2. THF (2.5 mL; dry, degassed) was added *via* a syringe and the immediately formed greenish-yellow suspension was stirred in an oil bath which was pre-heated to 40 °C. Another oven-dried Schlenk tube (*Flask 2*) was charged with 2,4-dibromopyridine (236.9 mg, 1.00 mmol, 1 equiv.) and *p*-fluorophenylboronic acid **2a** (167.9 mg, 1.20 mmol) and subsequently evacuated and backfilled with N_2. After 30 minutes of stirring, the contents of *Flask 1* were transferred into *Flask 2 via* a cannula and the resulting mixture was stirred for 5 mins in order to thermally equilibrate at 40 °C. Aqueous *Tetra-N-butylammonium* hydroxide (2.5 mL, 1.0 M, degassed) was then added (t_0) to commence the reaction.

Samples (*ca.* 125 µL), taken at the specified times using a 1000 µL syringe were rapidly quenched by dissolution in CH_2Cl_2 solution before being filtered through a pipette fitted with a Celite® plug (*ca.* 1 cm depth) (to remove black particles). The filtrate was concentrated in vacuum to reveal a reddish-brown oil which was dissolved in $CDCl_3$ (0.5 mL) for NMR sampling. Samples were analysed according to the procedure highlighted in Section 2.7.

Table S1 Detailing the ratio of $Pd_3(OAc)_6$ to PPh_3, alongside the quantity (mmol and mg) of triphenylphosphine PPh_3 used in each reaction.

$Pd_3(OAc)_6$:PPh_3 (mol%:mol%)	Pd:P ratio	Amount of PPh_3 (mmol)	Mass of PPh_3 (mg)
**1:0**	1:0	N/A	N/A
**1:1.5**	1:0.5	**0.015**	**3.9**
**1:3**	1:1	**0.03**	**7.9**
**1:4.5**	1:1.5	**0.045**	**11.8**
**1:6**	1:2	**0.06**	**15.7**
**1:7.5**	1:2.5	**0.075**	**19.7**
**1:9**	1:3	**0.09**	**23.6**
**1:12**	1:4	**0.12**	**31.5**

2.2. Base and Additive Effects – SMCC Reactions (Table 1 – Main Paper)

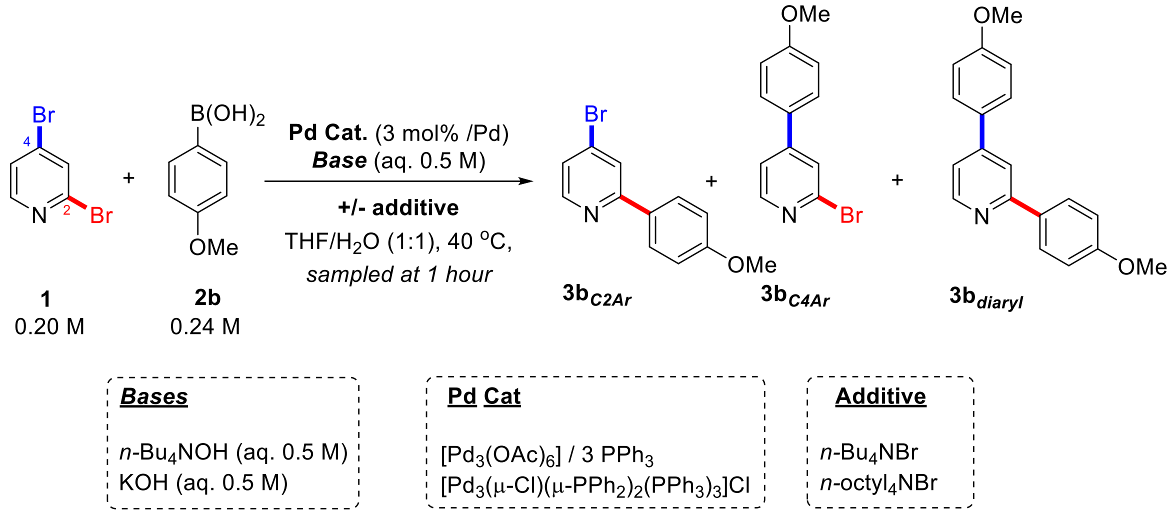

Scheme S2 Conditions and reagents for the investigation into base and additive effects on the model site-selective SMCC reaction at 1, catalysed by $[Pd_3(\mu\text{-}Cl)(\mu\text{-}PPh_2)_2(PPh_3)_3]Cl$ or $Pd(OAc)_2$/1 or 2 PPh_3.

An oven-dried Schlenk tube (Flask 1) charged with 2,4-dibromopyridine **1** (236.9 mg, 1.0 mmol), *para*-anisyl boronic acid **2b** (182.4 mg, 1.2 mmol) Pd Cat. (3 mol% Pd loading; see Table S2 for specifics) was evacuated and backfilled with N_2. Depending on the reaction, the salt additive was added at this point (See Table S3 for amounts used). The flask atmosphere was then evacuated and backfilled with N_2 before THF (2.5 mL; dry, degassed) was added *via* a syringe and stirred for 5 minutes at 40 °C (pre-heated oil bath). After this time, aqueous **base** (2.5 mL, 1.0 M, degassed) was added (t_0) to commence the reaction. The resulting reaction solution was hence stirred at 40 °C. Samples (*ca.* 125 μL), taken at the specified times using a 1000 μL syringe were rapidly quenched by

dissolution in CH_2Cl_2 solution before being filtered through a pipette fitted with a Celite® plug (*ca.* 1 cm depth) (to remove black particles). The filtrate was concentrated in vacuum to reveal a reddish-brown oil which was dissolved in $CDCl_3$ (0.5 mL) for NMR sampling. See Section 2.7, for details of sample analysis.

Table S2 Catalyst/PPh_3 amounts used

**Pd Cat.**	**m / mg**	**n / mmol**
$Pd_3(OAc)_6$ / {3PPh_3}	6.7 / {7.9}	0.01 / {0.03}
$Pd_3(OAc)_6$ / {6PPh_3}	6.7 / {15.7}	0.01 / {0.06}
$[Pd_3(\mu\text{-Cl})(\mu\text{-PPh}_2)_2(PPh_3)_3]Cl$	15.5	0.01

Table S3 Additive amounts used

Additive	m / mg	n / mmol
n-Bu_4NBr	805	2.5
n-Oct_4NBr	1367	2.5

2.3. Product Evolution Curves for Site-selective SMCC Reactions at 2,4-Dibromopyridine 30 (Figure 2 – Main Paper)

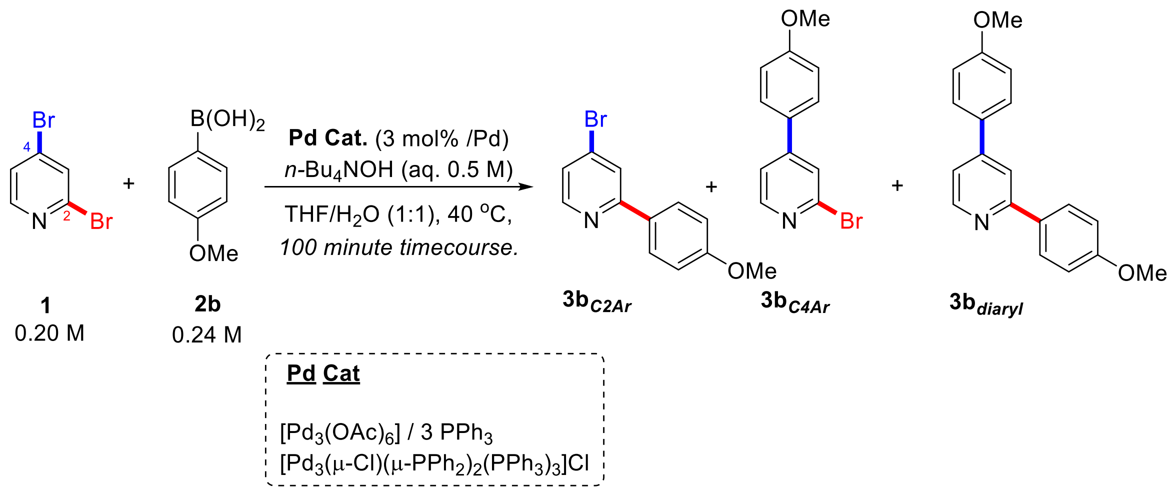

Scheme S3 Conditions and reagents for the product evolution assays on the model site-selective SMCC reaction between 1 and 2b, catalysed by $[Pd_3(\mu\text{-Cl})(\mu\text{-PPh}_2)_2(PPh_3)_3]Cl$ or $Pd(OAc)_2$/2PPh_3.

To an oven-dried Schlenk tube, 2,4-dibromopyridine **1** (473 mg, 2.0 mmol, 1.0 equiv.), *para*-anisylboronic acid **2b** (365 mg, 2.4 mmol, 1.2 eq) and 'Pd catalyst' (3 mol%/Pd, see Table 4) were added. The flask was sealed (Suba-seal®), THF (2.5 mL; dry, degassed) was added using a syringe and the resulting mixture was magnetically stirred for 5 minutes, in a pre-heated oil bath. After this time, all solids were seen to have dissolved into a clear

solution and the temperature of the reaction was measured at 40 ± 0.5 °C using an internal thermocouple. The cross-coupling reaction was then initiated by addition of *tetra-n*-butylammonium hydroxide solution (aq. 2.5 mL, 1.0 M→0.5 M, 2.5 equiv.) which was added by a rapid injection using a syringe over the Suba-seal®. The reaction was stirred, and samples were taken at the 0, 2.5, 5, 7.5, 10, 15, 20, 25, 20, 40, 50, 60, 80 and 100 minutes. Samples (*ca*. 100 µL), taken at the specified times *via* a 1000 µL syringe were rapidly quenched by dissolution in CH_2Cl_2 before being filtered through a pipette fitted with a Celite® plug (*ca.* 1 cm depth) (to get rid of any particulate Pd). Each filtrate was concentrated in vacuum to reveal a reddish-brown oil which was dissolved in $CDCl_3$ (0.5 mL) for NMR sampling. Each NMR spectrum was analysed according to the method reported below (Section 2.7) and the results were graphed using Origin 2018 software.

Table 4 Catalysts, loadings and masses used in product evolution assays (Graphs A & B, Figure 2 – main paper)

Pd Catalyst	mol / %	n / mmol	m / mg
$[Pd_3(\mu\text{-}Cl)(\mu\text{-}PPh_2)_2(\mu\text{-}PPh_3)_3]Cl$ (Pd_3Cl_2, Graph A)	1	0.02	30.9
$Pd_3(OAc)_6$/{$2PPh_3$} (Graph B)	1/{6}	0.02/{0.012}	13.5/{31.5}

2.4. Site-selectivity in Kumada-Corriu Cross-couplings at 1 (Table 2 – Main Paper)

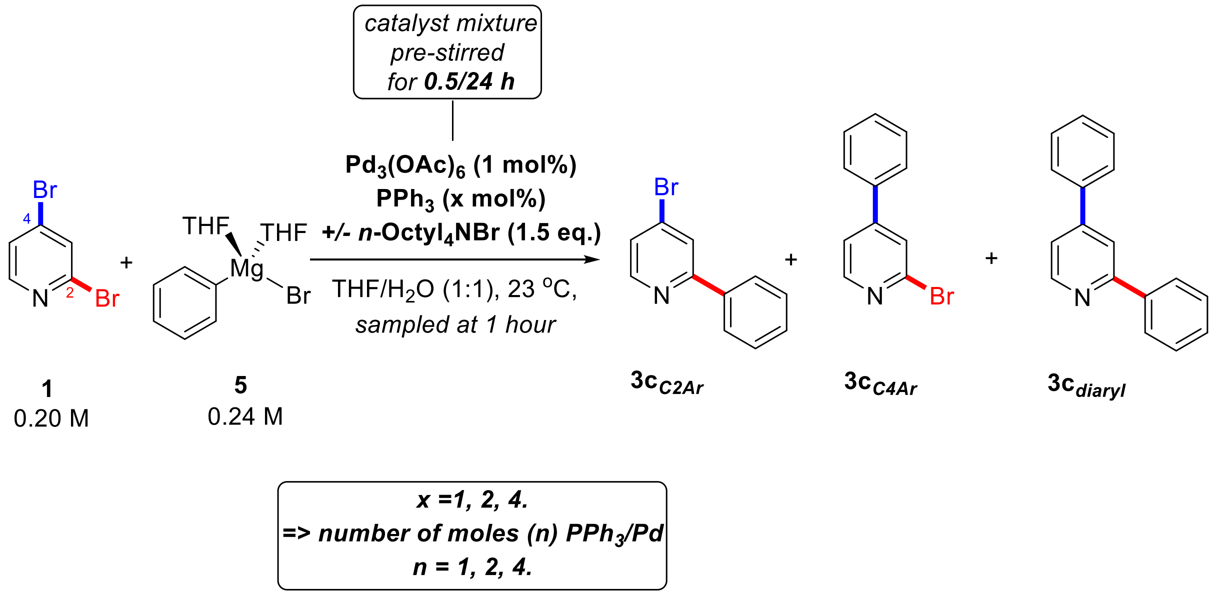

Scheme S4 Conditions and reagents used for the Kumada cross-coupling of 2,4-dibromopyridine 1 with phenylmagnesium bromide 5

A Schlenk flask (*Flask 1*) was charged with $Pd_3(OAc)_6$ (6.7 mg, 0.01 mmol, 1 mol%, Table S5) and PPh_3(3, 6, or 12 mol%, Table S5), tetra-*N*-octylammonium bromide (805.92 mg, 1.5 equiv.) and sealed (rubber septum) before being evacuated and backfilled with N_2 three times. THF (2.5 mL; dry, deoxygenated) was added and the resulting mixture was stirred at 23 °C for 0.5 or 24 hours.

A second Schlenk tube (Flask 2) was charged with 2,4-dibromopyridine **1** before being evacuated and backfilled with N_2 three times. THF (1.3 mL) was added and the resulting clear solution was stirred at room temperature. After the specified premixing time, the contents of *Flask 1* were transferred *via* cannula to *Flask 2*. The resulting mixture was allowed to equilibrate for 1 minute with stirring after which time phenylmagnesium bromide solution (**5**, 1.2 mL, 1.0 M in THF => total volume of 5 mL) was added in a rapid injection. The subsequent solution was stirred for one hour.

A sample (125 μL) was taken *via* a syringe and quickly quenched by addition to a vial containing 100 μL of NH_4Cl solution (*aq*., sat.). EtOAc (2 mL) was added to the sample and the resulting mixture was vigorously shaken. The EtOAc was removed using a pipette and the aqueous layer was extracted with another aliquot of EtOAc (2 mL). The combined organics were filtered through a $MgSO_4$ plug (*ca*. 1 cm depth) which was washed through with EtOAc (1 mL). The filtrate was concentrated *in vacuo* to afford a yellow oil which was dissolved in $CDCl_3$ and subjected to ^{1}H NMR analysis (see Section 2.7).

Table S5 Detailing ratio of $Pd_3(OAc)_6$ to PPh_3, alongside the quantity (mmol and mg) of triphenylphosphine PPh_3 used in this study.

$Pd_3(OAc)_6$:PPh_3 (mol%:mol%)	Pd:P ratio	Amount of PPh_3 (mmol)	Mass of PPh_3 (mg)
**1:3**	1:1	**0.03**	**7.9**
**1:6**	1:2	**0.06**	**15.7**
**1:12**	1:4	**0.12**	**31.5**

2.5. *Para*-substituent Effects on Site-selectivity in SMCC Reactions at 1 (Figures 3 & 4 – Main Paper)

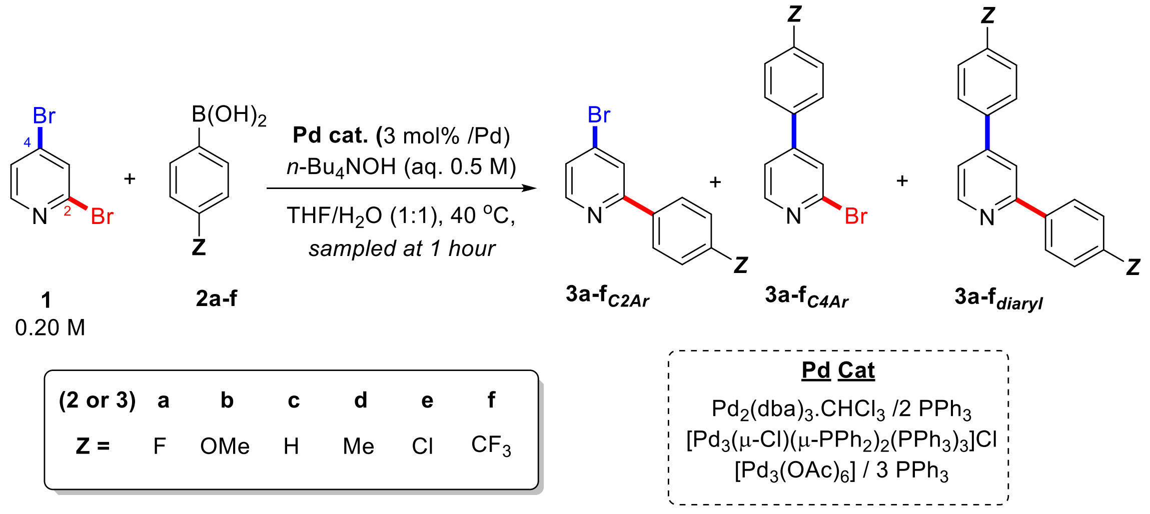

Scheme S5 Reagents and conditions used for investigating phenylboronic acid *para*-substituent effects on the site-selective SMCC reaction at 1.

An oven-dried Schlenk tube, charged with a Pd catalyst (3 mol%/Pd, see Table S6), 2,4-dibromopyridine **1** (236.9 mg, 1.0 mmol, boronic acid **2a-f** (1.2 mmol), (Scheme S5) was sealed (Suba-seal®) before being evacuated and backfilled with N_2. THF (2.5 mL; dry, degassed) was added *via* a syringe (over the Suba-seal®) and the resulting solution was stirred in an oil bath which was pre-heated to 40 °C for 5 mins in order to thermally equilibrate. *Tetra-n-butylammonium* hydroxide solution (2.5 mL, 1.0 M; degassed) was then added (t_0) to commence the reaction.

Samples (*ca.* 125 μL), taken at 1 hour using a 1000 μL (1 mL) gas-tight syringe were rapidly quenched by dissolution in CH_2Cl_2 solution before being filtered through a pipette fitted with a Celite® plug (*ca.* 1 cm depth) (to remove black particles). The filtrate was concentrated in vacuo to furnish a reddish-brown oil which was dissolved in $CDCl_3$ (0.5 mL) for NMR sampling (see Section 2.7).

Table S6 Catalyst amounts used

Pd Cat.	m / mg	n / mmol
$Pd_2(dba)_3.CHCl_3$ {$2PPh_3$}	16.5 {7.9}	0.015 {0.03}
$[Pd_3(\mu\text{-}Cl)(\mu\text{-}PPh_2)_2(PPh_3)_3]Cl$	15.5	0.01
$Pd_3(OAc)_6$ {PPh_3}	6.7 {7.9}	0.01{0.03}

2.6. General Workup Procedure for SMCC Reactions

The above cross-coupling reactions (from Sections 2.1-2.5) were worked-up using the following procedure. After the reaction time, the reaction solution was quenched using a of NH_4Cl (sat. aq). The organics were then extracted using EtOAc (4 × 10 mL) and combined before drying over $MgSO_4$, filtered and subsequently concentrated *in vacuo*. The resulting residue, which generally appeared as a reddish-brown oil, could be purified by column chromatography (SiO_2) using a hexane or petroleum ether/EtOAc solvent system. See Section 3 for purification details for specific compounds.

2.7. ^{1}H NMR Analysis of NMR Samples of Cross-coupling Reactions

The reaction conversion and site-selectivity of cross-coupling reactions at 2,4-dibromopyridine **1** were determined using a ^{1}H NMR spectroscopy-based assay of crude reaction samples (unless otherwise stated). Crude reaction samples were prepared according to the relevant method. Products were identified from the crude reaction mixtures by reference to literature published data or, if the product was not reported, by comparison to characterisation data obtained for the isolated product. The pyridyl-H_6 protons of the starting materials and products were generally well-resolved, due to the significant deshielding (proximity to electronegative N), thus, integration of these protons was generally used to determine the relative amounts of starting material and products; ***C2Ar***, ***C4Ar*** and ***diaryl*** *(see* Figure S1 for a typical example of how this integration was done for each reaction).

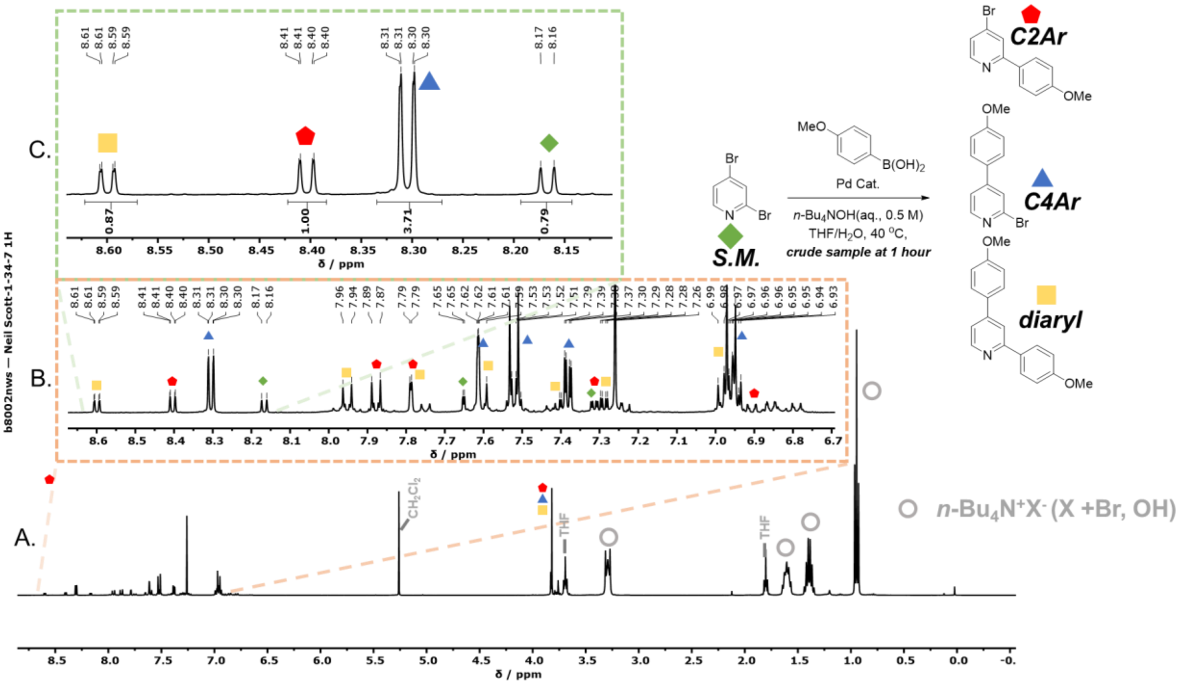

Figure S1 Method used for identifying and quantifying products of site-selective cross-coupling reactions at 2,4-dibromopyridine by ^{1}H NMR spectroscopy. A) crude NMR spectrum, B) expansion of aromatic region, allowing for the characterisation of products, C) Expansion of the *pyridine*-H_6 region, integration of which allowed for determination of reaction conversion and site-selectivity of the reaction.

How conversion and site-selectivity data was obtained:

e.g. **Overall Conversion** (%) = $\frac{integration\ C2+C4+diaryl}{integration\ \ S.M.+C2+C4+diaryl} \times 100 = \underline{\mathbf{87.6\%}}$

Conversion of given product *e.g.* C2 (%) = $\frac{integration\ C2}{integration\ \ S.M.+C2+C4+diaryl} \times 100 = \mathbf{15.7}\%$

Table 7 Example, processing of integration data

	S.M. (1)	***C4Ar***	***C2Ar***	***diaryl***
**Integral**	0.79	3.71	1.00	0.87
**Conversion (%)**	12.4	58.2	15.7	13.7

The above method was validated using an internal standard, 1,3,5-trimethoxybenzene. 1,3,5-Trimethoxybenzene (56.0 mg, 0.333 mmol) was added as a solid, alongside the other solids of the reaction mixture: $Pd(OAc)_2$/$1PPh_3$, 2,4-dibromopyridine **1** (1.0 mmol) and the *para*-substituted phenylboronic acid (**2a-f**, 1.20 mmol). The reaction was carried out and sampled as highlighted in Section 2.5. The sample was analysed by ^{1}H NMR spectroscopic analysis (Figure S2) (see Section 3 below for specific details concerning product identification).

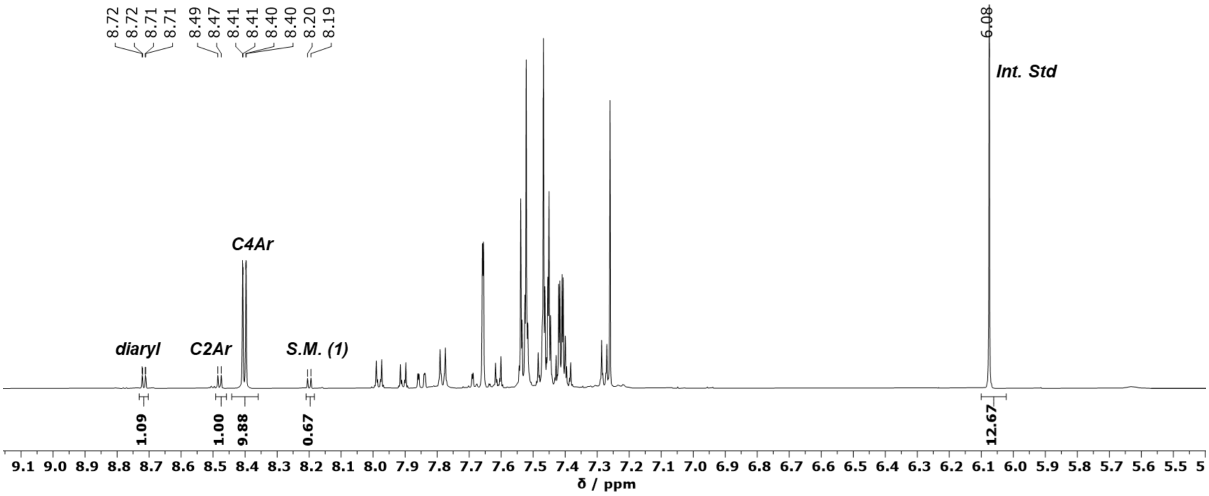

Figure S2 A ^{1}H NMR spectrum (400 MHz, $CDCl_3$), showing how the pyridine-H_6 peaks representing 1 and the three SMCC products were quantified against the aryl-H resonance of the 1,3,5-trimethoxybenzene internal standard ('Int Std') in a crude reaction sample.

The products could then be quantified against the internal standard as follows (Table S8), providing confidence in the assay as a validated quantitative method.

Table S8 An example of how the method of analysis of the crude SMCC reaction mixture was validated by use of a 1,3,5-trimethoxybenzene internal standard. *Internal standard = Peak at δ_H 6.08 ppm, representing 3 × Aryl-H protons was used.

	S.M. (1)	*C4Ar*	*C2Ar*	*diaryl*	*Int Std**
**Integral**	0.67	9.88	1.00	1.09	12.67
**Conversion (%)**	5.3	78.2	7.9	8.6	N/A
**Conversion (%) against Int Std***	5.3	78.0	8.0	8.6	N/A

2.8. Oxidative Addition Reaction of [Pd^0(PPh$_3$)$_4$] to 1 (Figure 6 – Main Paper)

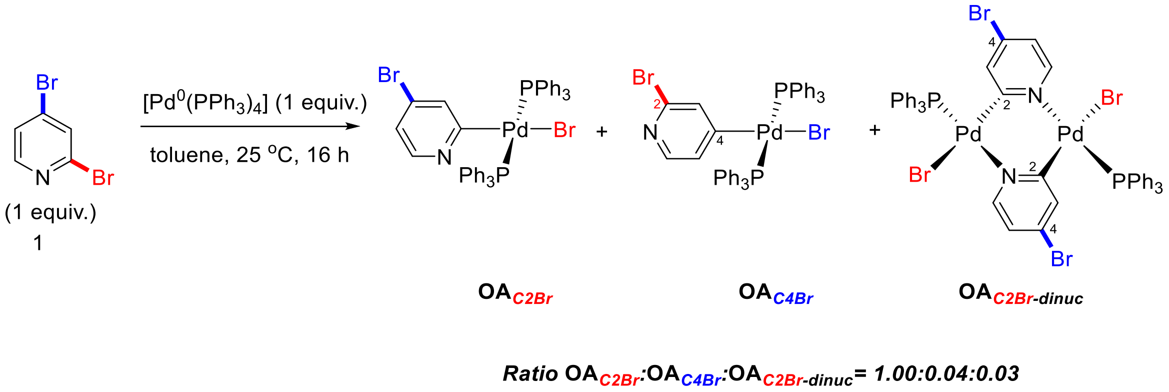

Scheme S6 The direct reaction between [Pd^0(PPh$_3$)$_4$] and 2,4-dibromopyridine 1.

$[Pd^0(PPh_3)_4]$ (0.20 g, 0.173 mmol) was treated with 2,4-dibromopyridine **1** (41.0 mg, 0.173 mmol) at room temperature, in toluene (0.5 mL dry, degassed) with stirring for 16 hours, according to the experimental method previously reported by Cid and co-workers (Scheme S6).[3] After removing the toluene solvent *in vacuo* and washing the residue with diethyl ether (3 × 0.5 mL; dry, degassed), ^{31}P spectroscopic analysis of a CD_2Cl_2 solution of the crude reaction product (a. Figure S3) confirmed the site-selectivity of the oxidative addition, with resonances at δ_P 22.8, 24.3, 27.6, respectively, representing products in a ratio of $\mathbf{OA_{C2Br}:OA_{C4Br}:OA_{C2Br\text{-}dinuc}}$ **= 1.00:0.04:0.03** (verified against data reported by Cid and co-workers) under the conditions.[3] According to ^{31}P NMR spectroscopic analysis, the C2/C4-selectivity for the oxidative addition is *ca.* 25:1, consistent with the overall C2-selectivity observed for the SMCC reactions employing $[Pd^0(PPh_3)_4]$.

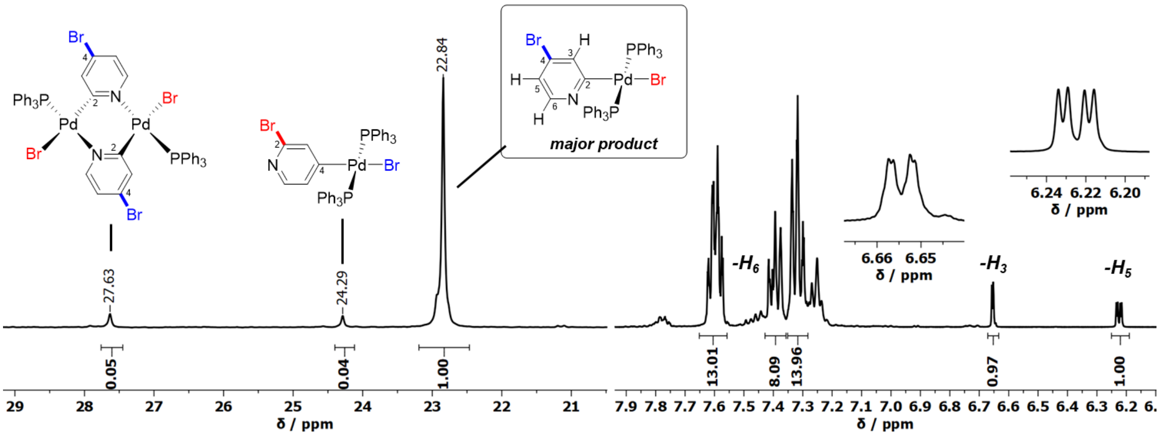

Figure S3 (LEFT.) ^{31}P NMR spectrum (162 MHz, CD_2Cl_2), showing the distribution of products oxidative addition of $[Pd^0(PPh_3)_4]$ to 1. (RIGHT.) ^1H NMR spectrum of the same crude sample (400 MHz, CD_2Cl_2), showing $OA_{C2\text{-}Br}$ as the major species, H_3 and H_5 protons on the 4-bromopyridiyl ligand have been assigned.

Crystals were grown by carefully layering the above CD_2Cl_2 solution with excess dry, degassed hexane (1:3 v/v). The crystalline product could therefore be isolated for further characterisation (*vide infra*) in a 26% yield of isolated $\mathbf{OA_{C2\text{-}Br}}$ product (unoptimized crystallisation). Upon subjecting one such crystal to XRD analysis, the solid-state structure was confirmed as being that of the $\mathbf{OA_{C2\text{-}Br}}$ oxidative addition product (Figure S4).

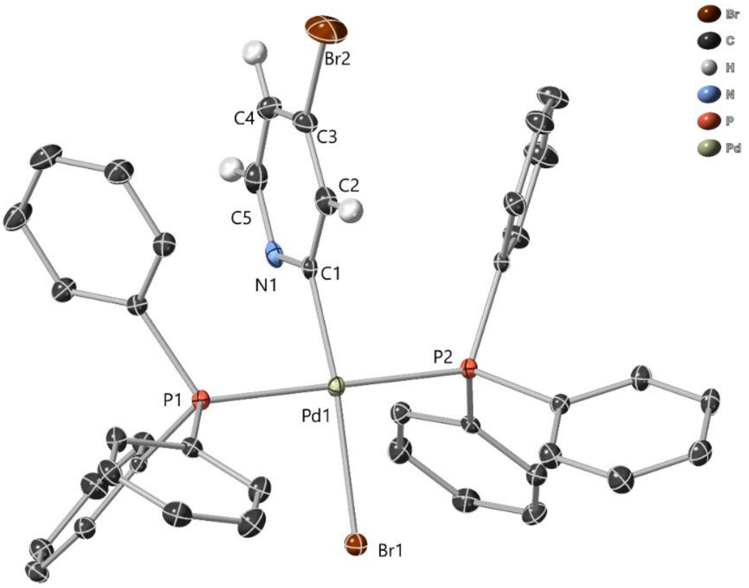

Figure S4 XRD structure of a single crystal of OA$_{C2\text{-}Br}$, obtained from the reaction of 2,4-dibromopyridine 1 with [$Pd^0(PPh_3)_4$].

^{1}H NMR spectroscopic analysis of a CD_2Cl_2 solution of the crude reaction product showed significantly shielded aromatic resonances, at δ_P 6.65 and 6.23 ppm (Right, Figure S3), which were assigned as –H_3 and –H_5 protons on the 4-brompyridyl ligand based on their coupling constants and relative integrations. This upfield-shifting of these proton resonances is likely a result of the interaction of the aromatic ring currents associated with the proximal phenyl moieties. Such an interaction is evident in the crystal structure (Figure S4), which shows an interfacial π-stacking interaction – the 4-bromopyridyl ligand is sandwiched between two phenyl groups from the *trans*-configured triphenylphosphine ligands, providing further support that the crystal obtained (XRD) is the major species observed by ^{1}H NMR spectroscopic analysis (in solution).

Characterisation Data for Bromo(4-bromo-C2-pyridinyl),bis(triphenylphosphine)Palladium^{II} OA$_{C2\text{-}Br}$[3]

(*N.B. in solution* **OA$_{C2\text{-}Br}$** converts over time to the dimer product **OA$_{C2\text{-}Br\text{-}dinuc}$** with release of uncoordinated PPh_3, therefore after isolation by crystallisation during solution characterisation of **OA$_{C2\text{-}Br}$** {by *e.g.* NMR spectroscopy} a mixture of the three species rapidly arises)

^{31}P NMR (202 MHz, dichloromethane -d_2) δ 22.84 (s) ppm.^{1}H NMR (500 MHz, dichloromethane-d_2) δ 7.64 – 7.54 (m, 12H, PPh_3), 7.44 – 7.36 (m, 7H, PPh_3 {6H} + *pyridyl*-C_6-H{1H}), 7.35 – 7.28 (m, 12H), 6.66 (d, *J* = 1.9 Hz, 1H, *pyridyl*-C_3-H), 6.23 (dd, *J* = 5.3, 1.9 Hz, 1H, *pyridyl*-C_5-H) ppm.

ESI-MS Data (+ve mode) mode: Found 786.0328 [M–Br]$^+$ Calc. (for $C_{42}H_{39}O_6P_2Pd_2$) 786.0301.

IR (v/cm^{-1}, ATR): 3049 (w, C–H), 1589 (m), 1535 (m), 1525 (m), 1480 (m), 1433 (s), 1346 (m), 1186 (m), 1094 (m), 998 (m), 807 (m), 740 (br, m), 690 (br, s), 517 (br, s), 499 (s).

Elemental analysis (% CHN), calculated for $C_{41}H_{33}Br_2NP_2Pd$: C, 56.74; H, 3.83; N, 1.61; Found: C, 56.55; H, 3.79; N, 1.61.

See Section 4 for X-Ray Crystallographic Data (ijsf1805).

2.9. Reaction of $Pd_3(OAc)_6$ with 3 Equivalents of PPh_3 (Figure 7 – Main Paper)

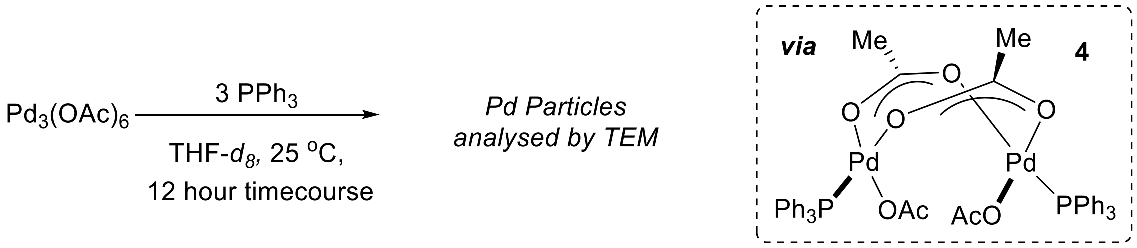

Scheme S7 Conditions used for the reaction between $Pd_3(OAc)_6$ and 3 PPh_3 (Pd:P = 1:1) in THF-*d_8*

NMR reaction monitoring: $Pd_3(OAc)_6$ (2.25 ± 0.05 mg, 3.3 μmol) and PPh_3 (1 equiv., 2.60 ± 0.05 mg) were carefully weighed and transferred into a J. Youngs NMR tube (in an Ar-filled Glove box). THF-*d_8* (0.5 mL) was added and the sample was shaken, forming a reddish-brown solution, which was swiftly introduced to an NMR spectrometer where data was collected (within *ca.* 15 minutes). The NMR evidence for the formation of **4** is discussed in the main paper. The presence of **4** was evident by detection of $[Pd^{II}(\mu_2\text{-OAc})(\kappa\text{-OAc})(PPh_3)]_2$ as a $[M–OAc]^+$ cation, m/z = 913.01877, by LIFDI-MS after immediate analysis of the reaction solution upon mixing $[Pd_3(OAc)_6]$ with 3 PPh_3 (*i.e.* Pd:PPh_3 = 1:1).Thusly, an NMR time course was recorded, tracking the degradation of **4** (δ_P 19.6 ppm) with time, along with the formation of unidentified degradation products. Integration data as a function of time was recorded using MNova software data analysis function and plotted in the form of a graph (Figure S5). Based on its chemical shift, the peak detected at δ_P 24.6 ppm was assigned to O=PPh_3, a minor product of this process. TEM imaging of similar post-reaction solutions has been reported by our group[1] (see later).

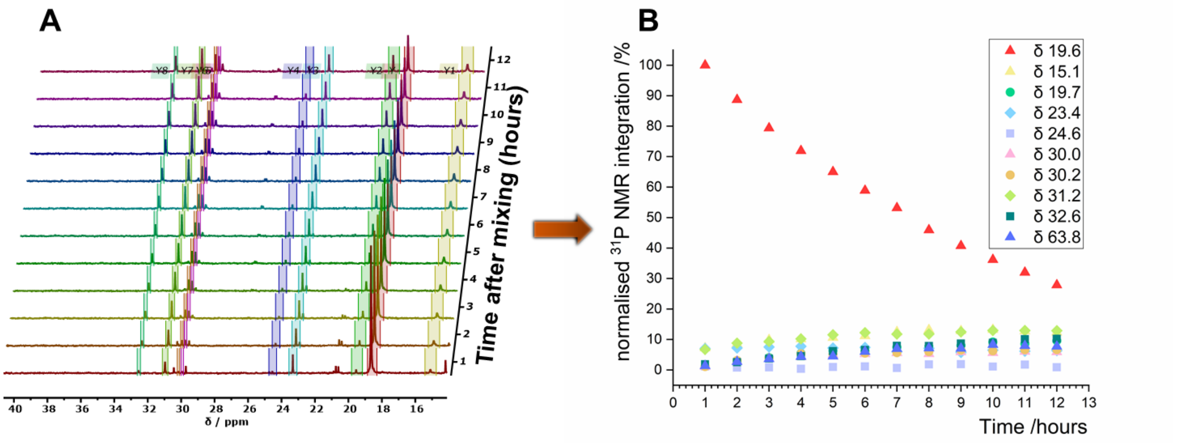

Figure S5 The process used to monitor the degradation of 4 and the concomitant formation of the new products over 12 hours. A: Stack of ^{31}P NMR spectra, recorded each hour throughout the process. B: Profiles for the ^{31}P resonances (integral data), as a function of time.

^{1}H NMR analysis of the degraded solution of $Pd_3(OAc)_6$ and 3 PPh_3

Acetic anhydride was detected by comparison of the ^{1}H NMR spectrum of the post-reaction mixture with the ^{1}H NMR spectrum containing an authentic sample, both in THF-d_8 (Figure S6). Acetic acid was also detected as a bi-product of the process and they appear in the post-reaction solution in an apparent molecular ratio (Ac_2O:AcOH) of 1:3 (based on integration of the ^{1}H resonances).[4] This observation is in-keeping with one acetate ligand being acylated *per* degradation of **4**, and the other three acetate ligands picking up a proton from the reaction mixture and converting to acetic acid. ^{1}H NMR spectroscopic analysis of the post-reaction mixture indicated significant degradation to acetate and phenyl-containing products (which are concomitant with the new phosphorus-containing species detected by ^{31}P NMR spectroscopic analysis).

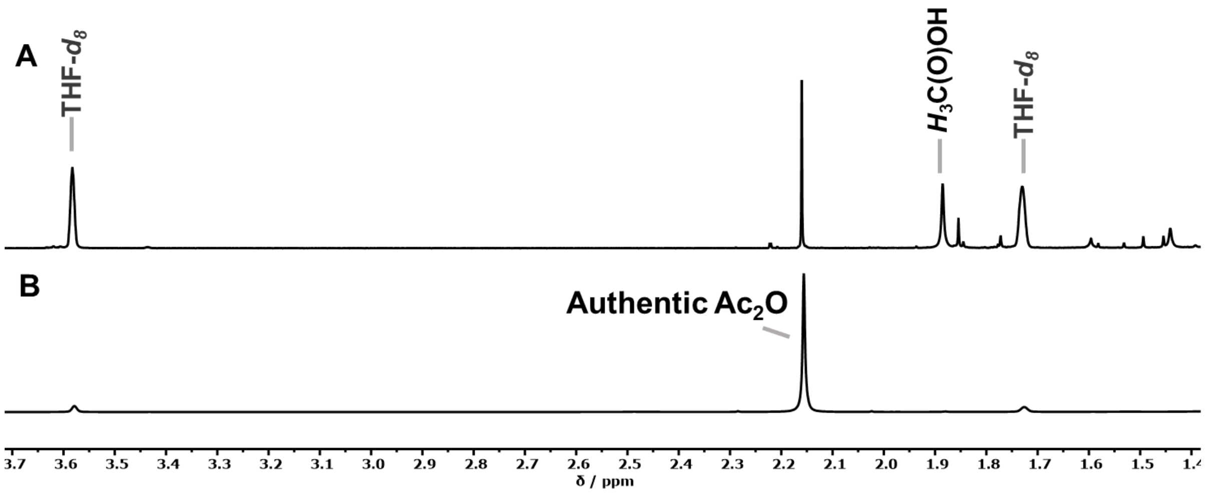

Figure S6 Expanded ^{1}H NMR stack of: A. the post-reaction solution of $Pd(OAc)_2$/1PPh_3 (*ca.* 12 hours after mixing); B. An authentic sample of acetic anhydride in THF-d_8.

Characterisation Data for $[Pd^{II}(\mu_2\text{-}OAc)(\kappa\text{-}OAc)(PPh_3)]_2$ 4[5]

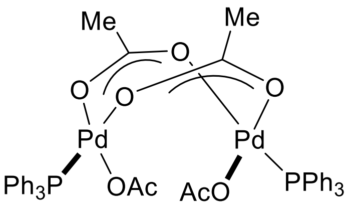

4 was not isolated in preparative form from solution due to its instability (*vide supra* and discussion in the main paper). The following data is presented for its characterisation in solution after direct reaction between $Pd_3(OAc)_6$ and 3 equivalents of PPh_3, in THF-d_8 as highlighted above.

^{1}H NMR (500 MHz, THF-d_8) δ 7.90 (m, 12H), 7.54 – 7.46 (br m, 6H), 7.36 (m, 12H), 1.35 (s, 12H).

^{31}P NMR (203 MHz, THF-d_8) δ 19.55 (s).

LIFDI-MS Data (+ve mode): Found 913.0309 $[M–OAc]^+$ Calculated (for $C_{42}H_{39}O_6P_2Pd_2$) 913.0188.

IR (ν/cm^{-1}, ATR): 1634 (s, κ-CO_{2asym}), 1557 (br,s, μ-CO_{2asym}), 1409 (s, μ-CO_{2sym}), 1309 (br, s, κ-CO_{2sym}).[5]

*Isolation of single crystal of **4***

A Schlenk tube was charged with $Pd_3(OAc)_6$ (6.7 mg, 0.01 mmol) and 3 equivalents of PPh_3 ({7.9 mg, 0.03 mmol}: Pd:P = 1:1). The Schlenk tube atmosphere was evacuated and backfilled with N_2 before being put on ice. THF (2.5 mL; pre-cooled to 0 ^{o}C) was added and the resulting mixture was stirred at 0 ^{o}C for 5 minutes, appearing as a brownish-red solution. A sample (0.5 mL) was taken *via* a syringe and added to a J. Youngs-type NMR tube under Schlenk conditions, on ice. The reaction mixture was then layered with pre-cooled hexane and swiftly stored at -18 ^{o}C. This crystal data is presented in the main paper and in Section 4 of this document.

3. Characterisation of Organic Products

3.1. 2,4-Disubstituted Pyridines: Suzuki-Miyaura/Kumada Cross-coupling Reactions

3.1.1. SMCC of 1 with 4-Fluorophenylboronic acid (2a)

Products could be identified from crude reaction mixtures (Figure S7). Characterisation data matched that reported in the literature,[3] and/or with data collated for (novel) isolated compounds reported *vide infra*.

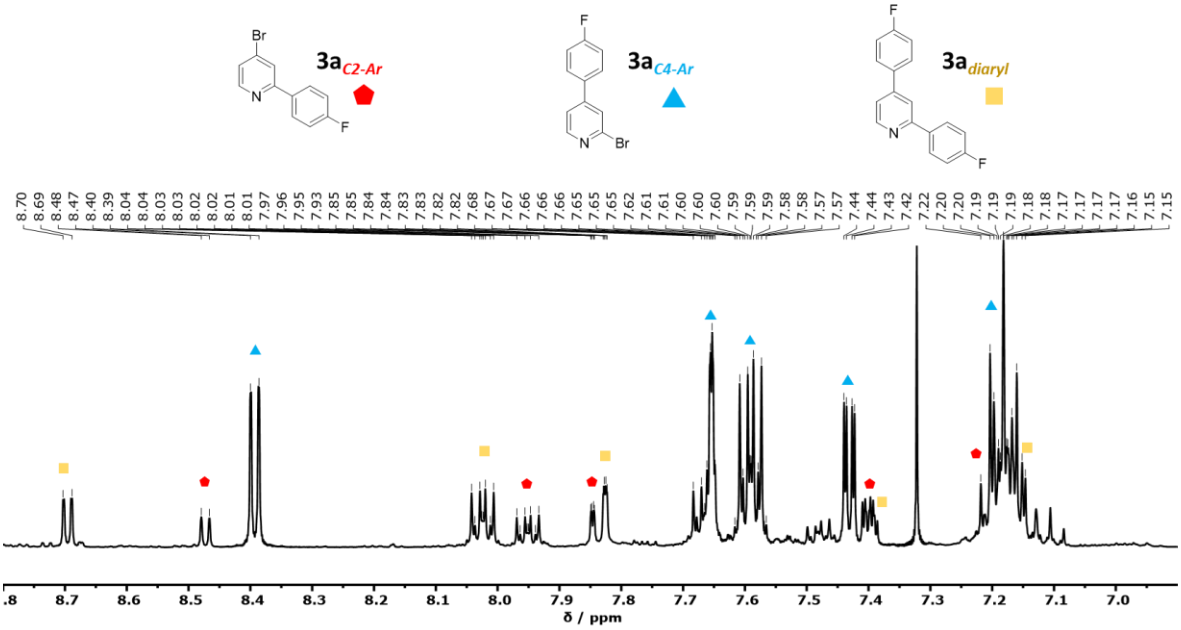

Figure S7 ^{1}H NMR spectrum (400 MHz, $CDCl_3$) showing 3a$_{C4-Ar}$, 3a$_{C2-Ar}$ and 3a$_{diaryl}$ product identification from a crude reaction mixture. (note: small differences in ^{1}H chemical shift from authenticated products are seen here).

2-Bromo-4-(4-fluorophenyl)pyridine (3a$_{C4\text{-}Ar}$)

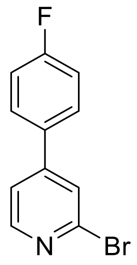

Although the title compound is commercially available, no literature data was found for its characterization or any alternative syntheses. It was isolated from the reaction mixture using flash chromatography (SiO_2) and a hexane/EtOAc (95:5) solvent system with a gradient, starting from neat hexane. Yield from the model SMCC, catalysed by **Pd_3Cl_2** (Section 2.5) = 161.9 mg (65 %). Appeared as a colorless solid. Melting point – 157.0-158.9 ^{o}C.

^{1}H NMR (400 MHz, Chloroform-*d*) δ 8.40 (dd, *J* = 5.1, 0.7 Hz, 1H, *pyridyl*-C_6-H), 7.66 (dd, *J* = 1.6, 0.7 Hz, 1H, *pyridyl*-C_3-H), 7.63 – 7.54 (m, 2H, *aryl*), 7.41 (dd, *J* = 5.2, 1.6 Hz, 1H, *pyridyl*-C_5-H), 7.24 – 7.13 (m, 2H) ppm.
^{13}C NMR (101 MHz, Chloroform-*d*) δ 163.8 (d, $^{1}J_{C\text{-}F}$ = 250.4 Hz, C–F), 150.6 (C), 150.2 (C–H), 143.1 (C), 132.9 (d, $^{4}J_{C\text{-}F}$ = 3.3 Hz, *aryl*-C_q), 129.0 (d, ^{3}J = 8.3 Hz, *aryl*-C–H), 125.8 (C–H), 120.8 (C–H), 116.4 (d, $^{2}J_{C\text{-}F}$ = 21.7 Hz, *aryl*-C–H) ppm.
^{19}F NMR (376 MHz, Chloroform-*d*) δ -111.18 (ddd, *J* = 13.7, 8.3, 5.2 Hz).
IR (v/cm^{-1}, ATR): 3051 (w), 1587 (m), 1528 (m), 1512 (m), 1455 (m), 1370 (m), 1300 (m), 1223 (m), 1159 (m), 1126 (m), 1102 (m), 1080 (m), 1040 (m), 986 (m), 963 (m), 883 (m), 822 (vs), 754 (m), 685 (m), 593 (m), 554 (s), 517 (m), 434 (m).
HRMS ESI-MS m/z = 251.9816 $[M+H]^+$: $C_{11}H_8BrFN$ requires 251.9824
R_f (hexane/EtOAc 95:5) = 0.11

4-Bromo-2-(4-fluorophenyl)pyridine (3a$_{C2\text{-}Ar}$)[3]

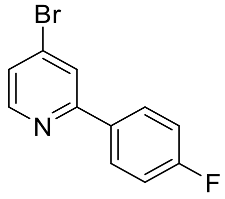

Compound characterisation data agrees with that previously reported in literature.[3] It was isolated from the reaction mixture using flash chromatography (SiO_2) with a hexane/EtOAc (95:5) solvent system, which was run

with a gradient, starting from neat hexane. Yield from the model SMCC (Section 2.5), catalysed by **Pd_3Cl_2** = 21 mg (8%). Appeared as a colourless oily film.

^{1}H NMR (400 MHz, Chloroform-*d*) δ 8.49 (d, *J* = 5.3 Hz, 1H, *pyridyl*-C_6-H), 8.01 – 7.91 (m, 2H, *aryl*), 7.86 (d, *J* = 1.8 Hz, 1H, *pyridyl*-C_3-H), 7.40 (dd, *J* = 5.2, 1.8 Hz, 1H, *pyridyl*-C_5-H), 7.22 – 7.11 (m, 2H, *aryl*) ppm.
^{13}C NMR (101 MHz, Chloroform-*d*) δ 164.0 (d, $^1J_{C-F}$ = 249.5 Hz, C_q), 157.9 (C), 150.5 (C–H), 134.3 (C), 133.8 (C), 129.1 (d, $^3J_{C-F}$ = 8.5 Hz), 125.4 (C–H), 123.8 (C–H), 116.0 (d, $^2J_{C-F}$ = 21.8 Hz) ppm.
^{19}F NMR (376 MHz, Chloroform-d) δ -111.70 (ddd, *J* = 13.8, 8.5, 5.5 Hz) ppm.
HRMS ESI-MS m/z = 251.9814[M+H]$^+$: $C_{11}H_8BrFN$ requires 251.9824.
R_f (TLC, hexane/EtOAc 95:5) = 0.29

2,4-Bis-(4-fluorophenyl)pyridine (3a*diaryl*)[6]

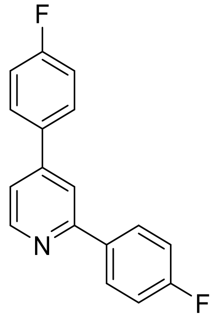

Compound characterisation data agrees with that previously reported in literature.[6] It was isolated from the reaction mixture using flash chromatography (SiO_2) a hexane/EtOAc (95:5) solvent system, which was run with a gradient, starting from neat hexane. Yield from an SMCC reaction (Section 2.5), catalysed by **Pd_3Cl_2** = 50.0 mg (19 %). Appeared as a colorless powder.

δ ^{1}H NMR (400 MHz, Chloroform-*d*) δ 8.71 (dd, *J* = 5.1, 0.7 Hz, 1H, *pyridyl*-C_6-H), 8.07 – 7.99 (m, 2H, *aryl*), 7.82 (dd, *J* = 1.7, 0.8 Hz, 1H, *pyridyl*-C_3-H), 7.69 – 7.62 (m, 2H, *aryl*), 7.39 (dd, *J* = 5.1, 1.7 Hz, 1H, *pyridyl*-C_5-H), 7.23 – 7.12 (m, 4H, *aryl*) ppm.
^{13}C NMR (101 MHz, Chloroform-*d*) δ 164.1 (d, $^1J_{C-F}$ = 249.8 Hz, C_q–F), 163.6 (d, $^1J_{C-F}$ = 250.6 Hz, C_q–F), 157.3 (CH, 150.3, 148.5, 135.7 (d, $^3J_{C-F}$ = 3.1 Hz, *aryl*-C–H), 134.7 (d, $^3J_{C-F}$ = 3.3 Hz *aryl*-C–H), 129.1 – 128.6 (m), 120.2 (C), 118.4 (C), 116.3 (d, $^2J_{C-F}$ = 21.7 Hz, *aryl*-C–H), 115.9 (d, *J* = 21.6 Hz, *aryl*-C–H) ppm.
^{19}F NMR (376 MHz, Chloroform-*d*) δ -112.35 (ddd, *J* = 13.3, 8.3, 5.2 Hz), -112.72 (ddd, *J* = 14.0, 8.6, 5.2 Hz) ppm.
HRMS ESI-MS m/z =268.0932 [M+H]$^+$: $C_{17}H_{12}F_2N$ requires 268.0938
R_f (TLC, hexane/EtOAc 95:5) = 0.05.

3.1.2. SMCC of 1 with 4-Anisylboronic acid (2b)

Products could be identified from crude reaction mixtures (Figure S8). Characterisation data matched that reported in the literature,[3] and/or with data collated for isolated compounds reported *vide infra*.

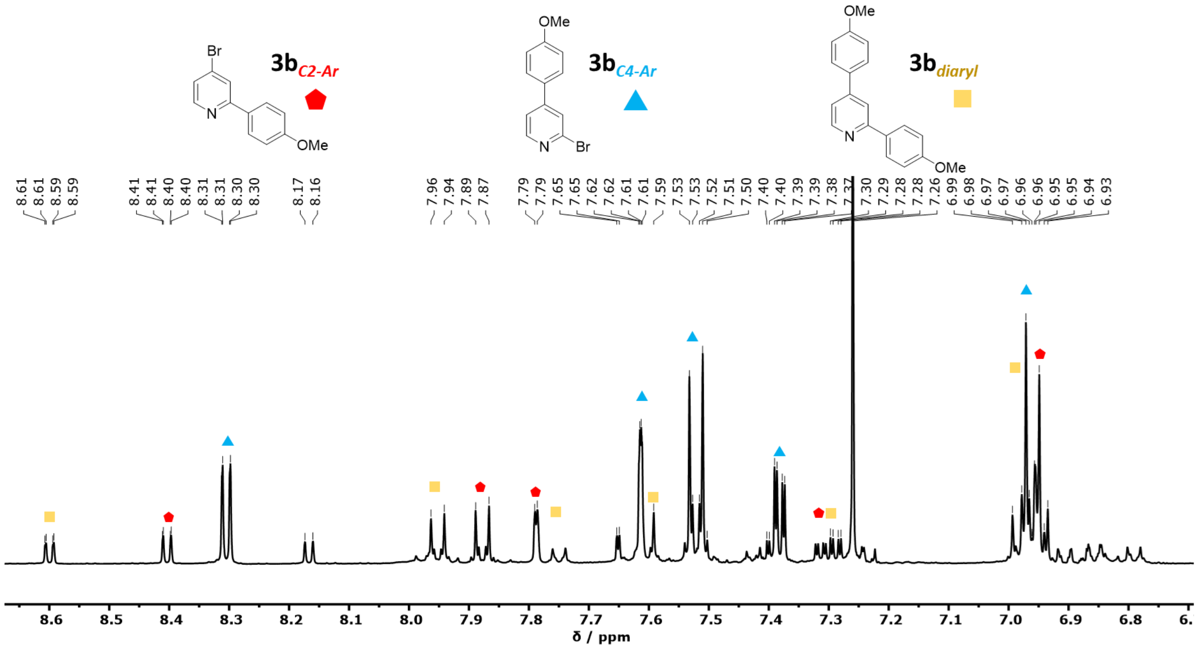

Figure S8 ^{1}H NMR spectrum (400 MHz, $CDCl_3$) showing 3b$_{C4\text{-}Ar}$, 3b$_{C2\text{-}Ar}$ and 3b$_{diaryl}$ product identification from a crude reaction mixture. (note: small differences in ^{1}H chemical shift from authenticated products are seen here).

2-Bromo-4-(4-anisyl)pyridine (3b$_{C4\text{-}Ar}$)[3]

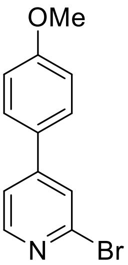

Compound characterisation data agrees with that previously reported in literature.[3] It was isolated from the reaction mixture using flash chromatography (SiO_2) and a hexane/EtOAc (95:5) solvent system, which was run with a gradient starting from neat hexane. Yield from the model SMCC (Section 2.1, 3.0 mmol scale in 2,4-dibromopyridine **1**), catalysed by $Pd_3(OAc)_6$/3PPh_3 = 510.5 mg (64 %). Appearance as a colorless powder. Melting point – 55.5-56.9 °C (57 °C lit.).[3]

^{1}H NMR (400 MHz, Chloroform-*d*) δ 8.37 (dd, *J* = 5.3, 0.7 Hz, 1H, *pyridyl*-C_{6}-H), 7.68 (dd, *J* = 1.7, 0.7 Hz, 1H, *pyridyl*-C_{3}-H), 7.62 – 7.53 (m, 2H, *aryl*), 7.44 (dd, *J* = 5.3, 1.7 Hz, 1H, *pyridyl*-C_{5}-H), 7.05 – 6.97 (m, 2H, *aryl*), 3.87 (s, 3H, OCH_3) ppm.

^{13}C NMR (101 MHz, Chloroform-*d*) δ 161.2 (C), 151.2 (C), 150.2 (C–H), 142.8 (C), 128.9 (C), 128.45(2C–H), 125.3 (C–H), 120.4 (2C–H), 114.9 (C–H), 55.6 (C–OCH_3) ppm.

IR (v, ATR): 3045 (w, C–H), 2935 (w, C–H), 2832 (w, C–H), 1609 (m), 1586 (s), 1515(s), 1457(m), 1429 (m), 1418(m), 1375 (m), 1292 (m), 1247 (vs), 1126 (m), 1082 (m), 1045 (m), 1018 (m), 986 (m), 815 (vs), 755 (m), 685 (m), 568 (m), 435 (m).

HRMS ESI-MS m/z = 264.0018 $[M+H]^+$: $C_{12}H_{11}{}^{79}BrNO$ requires 264.0019

Elemental analysis (% CHN), calculated for $C_{12}H_{10}BrNO$: C, 54.57; H, 3.82; N, 5.30; Found: C, 54.48; H, 3.88; N, 5.13.

R_f (TLC, Petroleum ether/EtOAc, 85:15) = 0.10.

4-Bromo-2-(4-anisyl)pyridine (3b*$_{C2-Ar}$*)[3]

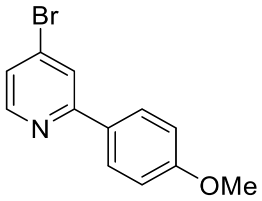

Compound characterisation data agrees with that previously reported in literature.[3] It was isolated from the reaction mixture using flash chromatography (SiO_2) with a hexane/EtOAc (95:5) solvent system, which was run with a gradient starting from neat hexane. Yield from the model SMCC (section 2.1, 3.0 mmol scale in 2,4-dibromopyridine **1**), catalysed by $Pd_3(OAc)_6/3PPh_3$ = 72.9 mg (9%). Appeared as a colorless powder.

^{1}H NMR (400 MHz, Chloroform-*d*) δ 8.46 (d, *J* = 5.3 Hz, 1H, *pyridyl*-C_{6}-H), 7.97 – 7.89 (m, 2H, *aryl*), 7.84 (d, *J* = 1.8 Hz, 1H, *pyridyl*-C_{3}-H), 7.34 (dd, *J* = 5.3, 1.8 Hz, 1H, *pyridyl*-C_{5}-H), 7.04 – 6.95 (m, 2H, *aryl*), 3.87 (s, 3H, OCH_3) ppm.

^{13}C NMR (101 MHz, Chloroform-*d*) δ 161.0 (C), 158.6 (C), 150.3 (C–H), 133.6 (C), 130.6 (C), 128.5 (C–H), 124.6(C–H), 123.2 (C–H), 114.3 (C–H), 55.5 C–OCH_3).

HRMS ESI-MS m/z = 264.0019 $[M+H]^+$: $C_{12}H_{11}{}^{79}BrNO$ requires 264.0019.

R_f (TLC, Petroleum ether/EtOAc, 85:15) = 0.27.

2,4-Bis(4-anisyl)pyridine (3b*diaryl*)[3]

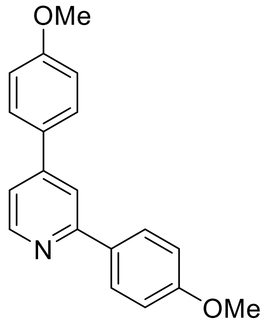

Compound characterisation data agrees with that previously reported in literature. [3] It was isolated from the reaction mixture using flash chromatography (SiO_2) with a hexane/EtOAc (95:5) solvent system, which was run with a gradient starting from neat hexane. Yield, from the model SMCC (Section 2.5, 3.0 mmol scale in 2,4-dibromopyridine **1**), catalysed by $Pd_3(OAc)_6/3PPh_3$ = 98.0 mg (11%). Appeared as a colorless powder.

^{1}H NMR (400 MHz, Chloroform-*d*) δ 8.65 (dd, *J* = 5.2, 0.8 Hz, 1H, *pyridyl*-C_6-H), 8.04 – 7.94 (m, 2H, *aryl*), 7.83 (dd, *J* = 1.8, 0.8 Hz, 1H *pyridyl*-C_3-H), 7.68 – 7.58 (m, 2H, *aryl*), 7.36 (dd, *J* = 5.2, 1.8 Hz, 1H, *pyridyl*-C5-H), 7.01 (dd, *J* = 8.8, 3.4 Hz, 4H, *aryl*), 3.86 (s, 6H, OCH_3, *overlapping*) ppm.
^{13}C NMR (101 MHz, Chloroform-*d*) δ 160.6 (2C), 157.6 (C) , 149.7 (C), 149.0 (C–H), 132.0 (C), 130.9 (C), 128.4 (C–H), 128.4 (C–H), 119.2 (C–H), 117.6 (C–H), 114.6(C–H), 114.2 (C–H), 55.5 (C–CH_3), 55.5 (C–OCH_3) ppm.
HRMS ESI-MS m/z = 292.1332 $[M+H]^+$: $C_{11}H_9FN$ requires 292.1338.
R_f (TLC, Petroleum ether/EtOAc, 85:15) = 0.07.

3.1.3. SMCC of 1 with Phenylboronic acid (2c)

Products could be identified from crude reaction mixtures (Figure S9). Characterisation data matched that reported in the literature,[3] and/or with data collated for isolated compounds reported *vide infra*.

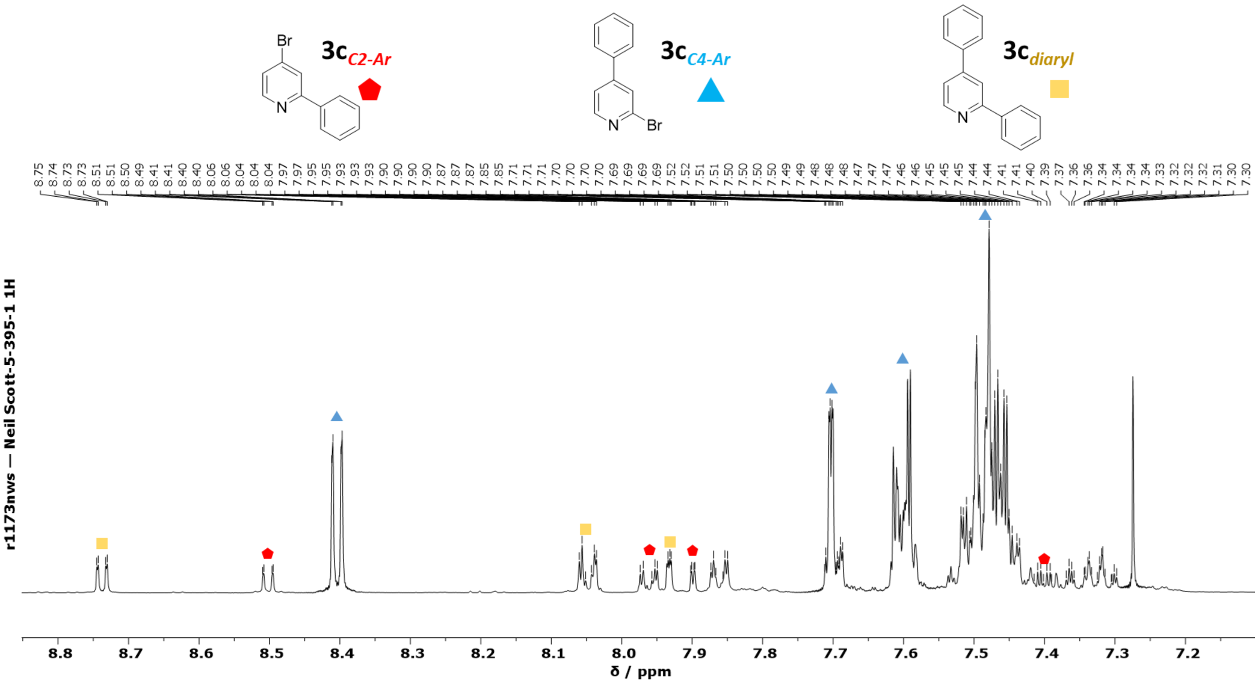

Figure S9 ^{1}H NMR spectrum (400 MHz, $CDCl_3$) showing $3c_{C4\text{-}Ar}$, $3c_{C2\text{-}Ar}$ and $3c_{diaryl}$ from a crude post-reaction mixture. (note: small differences in ^{1}H chemical shift from authenticated products are seen here).

2-Bromo-4-phenylpyridine ($3c_{C4\text{-}Ar}$)[3]

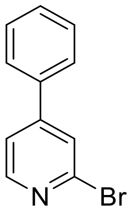

Compound characterisation data agrees with that previously reported in literature.[3] It was isolated from the reaction mixture, catalysed by **Pd_3Cl_2** (Section 2.5) using flash chromatography (SiO_2) with a hexane/EtOAc (95:5) solvent system, which was run with a gradient starting from neat hexane. Yield = 145.5 mg (61%) Appeared as a colorless powder. Melting point – 65.0-67.1 °C.[3]

^{1}H NMR (400 MHz, Chloroform-*d*) δ 8.41 (dd, *J* = 5.2, 0.7 Hz, 1H, *pyridyl*-C_6-H), 7.70 (dd, *J* = 1.7, 0.7 Hz, 1H *pyridyl*-C_3-H), 7.65 – 7.56 (m, 2H, *aryl*), 7.54 – 7.42 (m, 4H, *pyridyl*-C_5-H{1H}, *aryl*{3H}) ppm.

^{13}C NMR (101 MHz, Chloroform-*d*) δ 151.5 (C), 150.5(C–H), 143.0 (C), 136.8 (C), 129.8 (*aryl*-C–H), 129.4 (*aryl*-C–H), 127.2 (*aryl*-C–H), 126.0 (C–H), 121.0 (C–H) ppm.

IR (v/cm^{-1}, ATR): 3057 (d, C–H), 1584 (m), 1526 (m), 1502 (m), 1454 (m), 1444 (m), 1369 (m), 1335 (w), 1317 (w), 1135 (m), 858 (m), 755 (m), 698, (m), 690 (s), 611 (m), 599 (m).

HRMS ESI-MS m/z = 232.1122 $[M+H]^+$: $C_{17}H_{14}{}^{79}BrN$ requires 232.1121.

Elemental analysis (% CHN), calculated for $C_{11}H_8BrN$: C, 56.44; H, 3.44; N, 5.98 Found: C, 56.48; H, 3.51; N, 5.89.

4-Bromo-2-phenylpyridine (3c$_{C2\text{-}Ar}$)

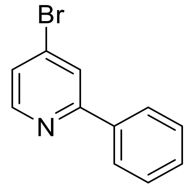

Reaction data for this compound, characterised as part of the crude post-reaction mixture, matched that previously reported in literature (see Figure S9 above). [3]

^1H NMR (400 MHz, Chloroform-*d*) δ 8.49 (d, *J* = 5.2 Hz, 1H, *pyridyl*-C_6-H), 7.95 (m, 2H, *aryl*), 7.88 (dd, *J* = 1.8, 0.6 Hz, 1H, *pyridyl*-C_3-H), 7.65 – 7.54 (m, 2H, , *aryl*). 7.39 (dd, *J* = 5.3, 1.8 Hz,1H, *pyridyl*-C_5-H) ppm.

HRMS ESI-MS m/z = 232.1122 $[M+H]^+$: $C_{17}H_{14}N$ requires 232.1121.

2,4-Bis-phenylpyridine (3c$_{diaryl}$)

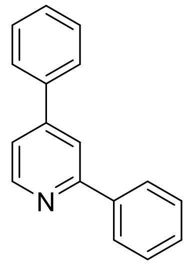

Reaction data for this compound, obtained as part of the crude post-reaction mixture, matched that previously reported in literature (see Figure S9 above). Appearance, colorless powder[3]

^1H NMR (400 MHz, Chloroform-*d*) δ 8.75 (d, *J* = 5.1 Hz, 1H, *pyridyl*-C_6-H), 8.09 – 8.02 (m, 2H, *aryl*), 7.94 (dd, *J* = 1.8, 0.8 Hz, 1H, *pyridyl*-C_3-H), 7.74 – 7.67 (m, 2H, *aryl*), 7.56 – 7.40 (m, 7H, *aryl* {6H}, *pyridyl*-C_5-H {1H}) ppm.

HRMS ESI-MS m/z = 233.9912 $[M+H]^+$: $C_{11}H_9{}^{79}BrN$ requires 233.9913.

3.1.4. SMCC of 1 with 4-Tolylboronic acid (2d)

Products could be identified from crude reaction mixtures (Figure S10). Characterisation data matched that reported in the literature,[3, 7-8] and/or with data collated for isolated compounds reported *vide infra*.

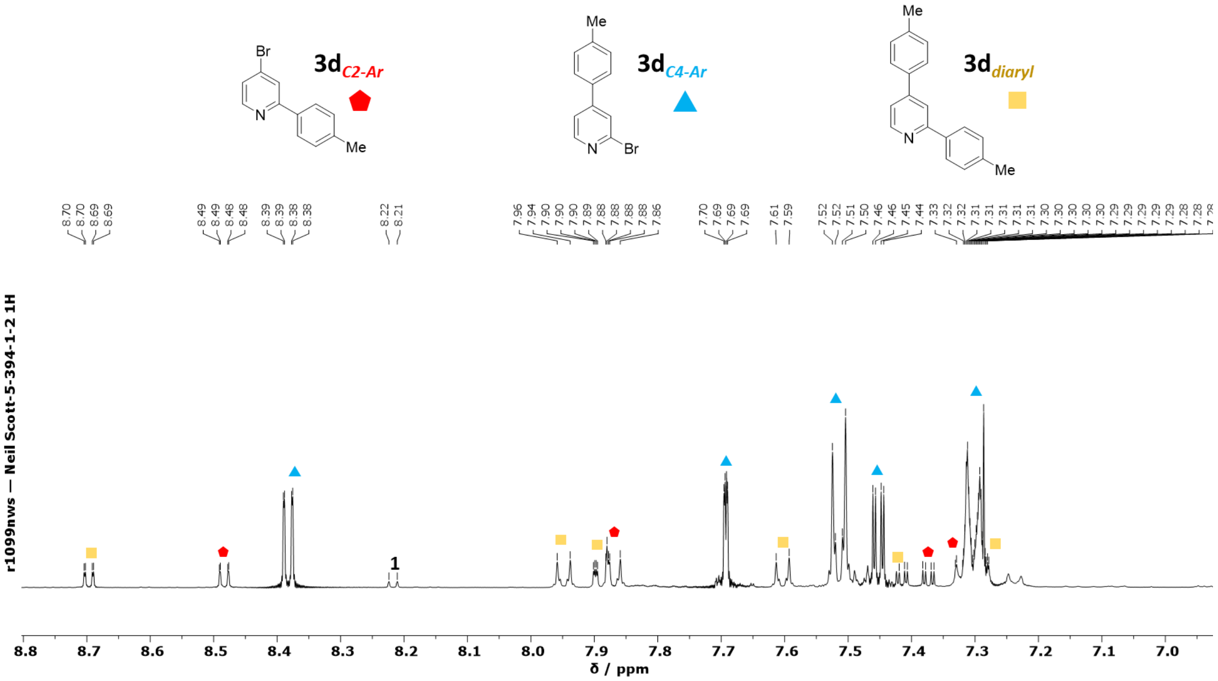

Figure S10 ^{1}H NMR spectrum (400 MHz, $CDCl_3$) showing 3d$_{C4-Ar}$, 3d$_{C2-Ar}$ and 3d$_{diaryl}$ product identification from a crude reaction mixture. (note: small differences in ^{1}H chemical shift from authenticated products are seen here).

2-Bromo-4-(4-tolyl)pyridine (3d$_{C4-Ar}$)[8]

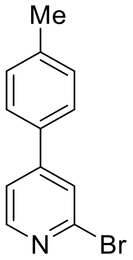

Compound characterisation data agrees with that previously reported in literature.[8-9] It was isolated from the reaction mixture using flash chromatography (SiO_2) with a hexane/EtOAc (95:5 v/v) solvent system (Section 2.5, catalysed by **Pd_3Cl_2**), which was run with a gradient. Yield = 133.7 mg (54%). Appeared as a colorless powder. Melting point – 72.5-72.9 °C (68-70°C lit.).[9]

^{1}H NMR (400 MHz, Chloroform-*d*) δ 8.38 (dd, *J* = 5.2, 0.7 Hz, 1H, *pyridyl*-C_{6}-H), 7.69 (dd, *J* = 1.7, 0.7 Hz, 1H, *pyridyl*-C_{3}-H), 7.53 – 7.48 (m, 2H, *aryl*), 7.44 (dd, *J* = 5.2, 1.7 Hz, 1H, *pyridyl*-C_{5}-H), 7.33 – 7.27 (m, 2H, *aryl*), 2.42 (s, 3H, C*H*$_3$) ppm.

^{13}C NMR (101 MHz, Chloroform-*d*) δ 151.4 (C), 150.4 (C–H), 143.0 (C–H), 140.2 (C) 133.8 (C), 130.1 (C–H), 127.0 (C–H), 125.7 (C–H), 120.7 (C), 21.4 (C–CH_3) ppm.

IR (v/cm^{-1}, ATR): 3011 (w, C–H), 2912 (w, C–H), 1571 (m), 1543 (m), 1511 (m), 1450 (m), 1106 (m), 1042 (m), 984 (m), 866 (m), 802 (vs), 751 (m), 681 (m), 502 (m), 424 (s).

HRMS ESI-MS m/z = 248.0067 [M+H]$^+$: $C_{12}H_{11}{}^{79}BrN$ requires 248.0069.

Elemental analysis (% CHN), calculated for $C_{12}H_{10}BrN$: C, 58.09; H, 4.06; N, 5.65, Found: C, 57.89; H, 4.08; N, 5.54.

R_f (TLC, hexane/EtOAc 95:5 (*v/v*) = 0.17.

4-Bromo-2-(4-tolyl)pyridine (3d*$_{C2-Ar}$*)

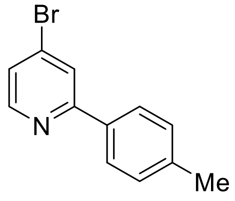

Compound characterisation data agrees with that previously reported in literature.[3] It was isolated from the reaction mixture using flash chromatography (SiO_2) with a hexane/EtOAc (95:5, v/v) solvent system (Section 2.5, catalysed by **Pd_3Cl_2**), which was run with a gradient, starting from neat hexane. Yield = 28.3 mg (11%). Appeared as a colourless oil.

^{1}H NMR (400 MHz, Chloroform-*d*) δ 8.48 (d, *J* = 5.3 Hz, 1H, *pyridyl*-C_{6}-H), 7.88 (s, 1H, *pyridyl*-C_{3}-H), 7.87–7.84 (m, 1H, *aryl*), 7.37(dd, *J* = 5.3, 1.8 Hz, 1H, *pyridyl*-C_{5}-H), 7.32–7.27 (m, 2H, *aryl*), 2.41 (s, 3H, C*H*$_3$) ppm.

^{13}C NMR (101 MHz, Chloroform-*d*) δ 159.0 (C), 150.4 (C–H), 139.9 (C), 135.3 (C), 133.5 (C), 129.7 (C–H), 127.0(C–H), 125.0 (C–H), 123.7 (C–H), 21.4 (C–*C*H_3) ppm.

IR (v/cm^{-1}, ATR): 3009 (w, C–H) 2959 (br,w, C–H), 2852(w, C–H), 1569 (m), 1542 (m), 1510 (m), 1468 (m), 1448 (m), 1408 (m), 1374 (m), 1314 (m), 1260 (m), 1091 (m), 1044 (m), 1015 (m), 807 (vs), 749 (m), 731 (m), 660 (m), 585 (m), 426 (m).

HRMS ESI-MS m/z = 248.0067 [M+H]$^+$: $C_{12}H_{11}{}^{79}BrN$ requires 248.0069.

R_f (TLC, hexane/EtOAc 95:5 (*v/v*) = 0.28

2,4-Bis(4-tolyl)pyridine (3d*diaryl*)[7]

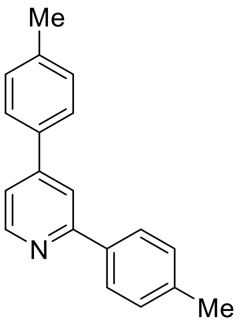

Compound characterisation data agrees with that previously reported in literature. It was isolated from the reaction (Section 2.1, catalyzed by **Pd_3Cl_2**) mixture using flash chromatography with a hexane/EtOAc (95:5 v/v) solvent system, which was run with a gradient starting from neat hexane. Yield = 24.6 mg (9%). Appeared as a colorless powder.

^{1}H NMR (400 MHz, Chloroform-*d*) δ 8.70 (dd, *J* = 5.2, 0.8 Hz, 1H, *pyridyl*-C_6–H), 8.02 – 7.93 (m, 2H, *aryl*), 7.90 (dd, *J* = 1.7, 0.8 Hz, 1H *pyridyl*-C_3–H), 7.70 – 7.55 (m, 2H, *aryl*), 7.41 (dd, *J* = 5.2, 1.7 Hz, 1H, *pyridyl*-C_5–H), 7.26-7.35 (m, 4H, *aryl*), 2.43 (s, 6H, 2 × CH_3 {overlapping}).
HRMS ESI-MS m/z = 260.1437 $[M+H]^+$: $C_{19}H_{18}N$ requires 260.1434.
R_f (TLC, hexane/EtOAc 95:5 (*v/v*) = 0.10.

3.1.5. SMCC of 1 with 4-Chlorophenylboronic acid (2e)

Products could be identified from crude reaction mixtures (Figure S11). Characterisation data matched that reported in the literature,[7, 10-11] and/or with data collated for (novel) isolated compounds reported *vide infra*.

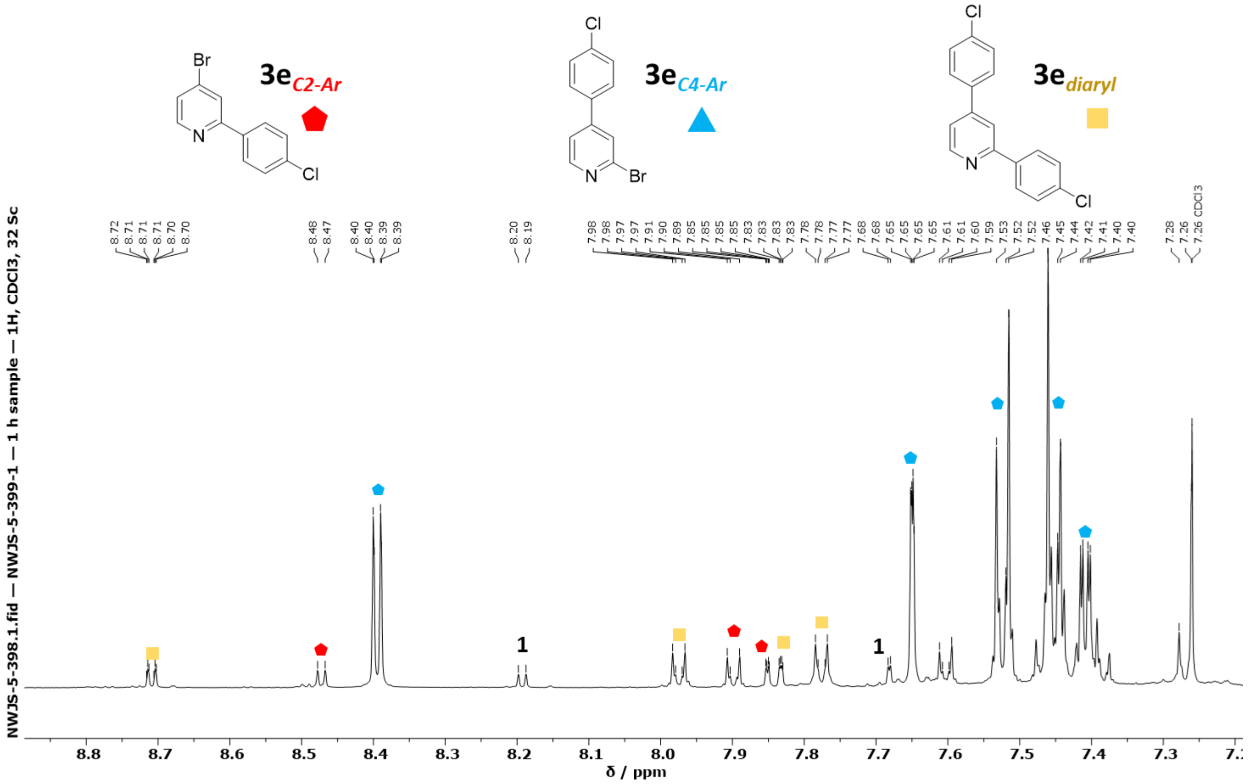

Figure S11 ^{1}H NMR spectrum (500 MHz, $CDCl_3$) showing 3e$_{C4\text{-}Ar}$, 3e$_{C2\text{-}Ar}$ and 3e$_{diaryl}$ compound identification from a crude reaction mixture. (note: small differences in ^{1}H chemical shift from authenticated products are seen here).

2-Bromo-4-(4-chlorophenyl)pyridine (3e$_{C4\text{-}Ar}$)[10]

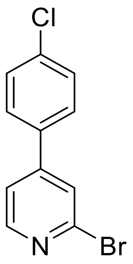

Compound characterisation data agrees with that previously reported in literature. It was isolated from the reaction mixture (Section 2.5, catalyzed by **Pd_3Cl_2**) using flash chromatography (SiO_2) with a hexane/EtOAc (98:2 v/v) solvent system, which was run with a gradient starting from neat hexane. Yield from reaction catalysed by **Pd_3Cl_2** = 110.7 mg (41%). Appeared as a colorless powder. Melting point – 138.2-139.5 °C (129-131°C lit.).[10]

^{1}H NMR (400 MHz, Chloroform-*d*) δ 8.41 (dd, *J* = 5.2, 0.7 Hz, 1H, *pyridyl*-C_6-H), 7.66 (dd, *J* = 1.7, 0.7 Hz, 1H, *pyridyl*-C_3-H), 7.57 – 7.50 (m, 2H, *aryl*), 7.50 – 7.44 (m, 2H, *aryl*), 7.42 (dd, *J* = 5.2, 1.7 Hz, 1H, *pyridyl*-C_5-H) ppm.
^{13}C NMR (101 MHz, Chloroform-*d*) δ 150.7 (*pyridyl*-C–H), 150.1 (C), 143.2 (C), 136.1 (C), 135.2 (C), 129.6 (*aryl*-C–H), 128.4 (*aryl*-C–H), 125.8 (*pyridyl*-C–H), 120.7 (*pyridyl*-C–H) ppm.
IR (v/cm^{-1}, ATR): 3053 (w, C–H), 3018(w, C–H), 1586 (s), 1525 (m), 1496 (m), 1454 (m), 1406 (m), 1370 (m), 1093(s), 1081 (m), 1043 (m), 1012 (m), 792 (s), 760 (s), 509 (m), 475 (m), 422 (m).
HRMS ESI-MS m/z = 267.9519 $[M+H]^+$: $C_{11}H_8{}^{79}BrClN$ requires 267.9523
Elemental analysis (% CHN), calculated for $C_{11}H_7BrClN$: C, 49.20; H, 2.63; N, 5.22; Found: C, 49.05; H, 2.54; N, 5.03.
R_f (TLC, PET/EtOAc, 98:2, {v/v}) = 0.33.

4-Bromo-2-(4-chlorophenyl)pyridine (3e*$_{C2-Ar}$*)[11]

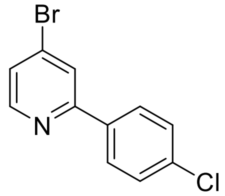

Compound characterisation data agrees with that previously reported in literature.[11] It was isolated from the reaction mixture (Section 2.5) using flash chromatography (SiO_2) with a hexane/EtOAc (98:2 v/v) solvent system, which was run with a gradient. Yield from reaction catalysed by **Pd_3Cl_2** = 34.7 mg (12.9 %). Appeared as a colorless powder.

^{1}H NMR (400 MHz, Chloroform-*d*) δ 8.49 (dd, *J* = 5.3, 0.6 Hz, 1H, *pyridyl*-C_6-H), 7.98 – 7.88 (m, 2H, *aryl*), 7.87 (dd, *J* = 1.8, 0.6 Hz, 1H, *pyridyl*-C_3-H), 7.54 – 7.37 (m, 5H, aryl{4H} *pyridyl*-C_5-H {1H}) ppm.
^{13}C NMR (101 MHz, Chloroform-*d*) δ 157.77, 150.58, 136.59, 135.99, 133.76, 129.23, 128.42, 125.68, 123.85 ppm.
IR (v/cm^{-1}, ATR): 2957(w), 2920 (w), 2852 (w), 1586 (m), 1570 (m), 1542 (m), 1455 (m), 1372 (m), 1076 (m), 1041 (m), 1015 (m), 985 (m), 802 (vs), 750 (m), 681 (m), 574 (m), 556 (m), 526 (m), 501 (m), 473 (m), 424 (m).
HRMS ESI-MS m/z = 267.9518 $[M+H]^+$: $C_{11}H_8BrClN$ requires 267.9523
R_f (TLC, PET/EtOAc {98:2}) = 0.22.

2,4-Bis(4-chlorophenyl)pyridine (3e$_{diaryl}$)[7]

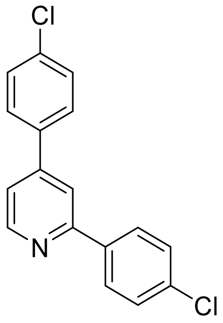

Compound characterisation data agrees with that previously reported in literature.[7] It was isolated from the reaction mixture (Section 2.5) using flash chromatography with a hexane/EtOAc (98:2) solvent system, which was run with a gradient starting from neat hexane. Yield from reaction catalysed by **Pd_3Cl_2** = 22.9 mg (8%). Appeared as a colorless powder.

^{1}H NMR (400 MHz, Chloroform-*d*) δ 8.73 (dd, *J* = 5.1, 0.8 Hz, 1H, *pyridyl*-C_6-H), 8.04 – 7.93 (m, 2H, *aryl*), 7.85 (dd, *J* = 1.7, 0.8 Hz, 1H, *pyridyl*-C_3-H), 7.67 – 7.56 (m, 2H, *aryl*), 7.53 – 7.43 (m, 4H, *aryl*), 7.42 (dd, *J* = 5.1, 1.7 Hz, 1H, *pyridyl*-C_5-H) ppm.

^{13}C NMR (101 MHz, Chloroform-*d*) δ 157.16 (C), 150.46 (*pyridyl*-C–H), 148.38 (C), 137.84 (C), 136.93 (C), 135.57 (C), 135.47 (*aryl*-C–H), 129.54 (*aryl*-C–H), 129.14 (*aryl*-C–H), 128.50 (*aryl*-C–H), 128.44 (*aryl*-C–H), 120.40 (*pyridyl*-C–H), 118.40 (*pyridyl*-C–H) ppm.

HRMS ESI-MS m/z = 300.0338 $[M+H]^+$: $C_{17}H_{12}Cl_2N$ requires 300.0347.

R_f (TLC, PET/EtOAc, 98:2 {v/v}) = 0.11.

3.1.6. SMCC of 1 with 4-Trifluoromethyl-phenylboronic acid (2f)

Products could be identified from crude reaction mixtures (Figure S12). Characterisation data matched that reported in the literature,[7, 12] and/or with data collated for isolated compounds reported *vide infra*.

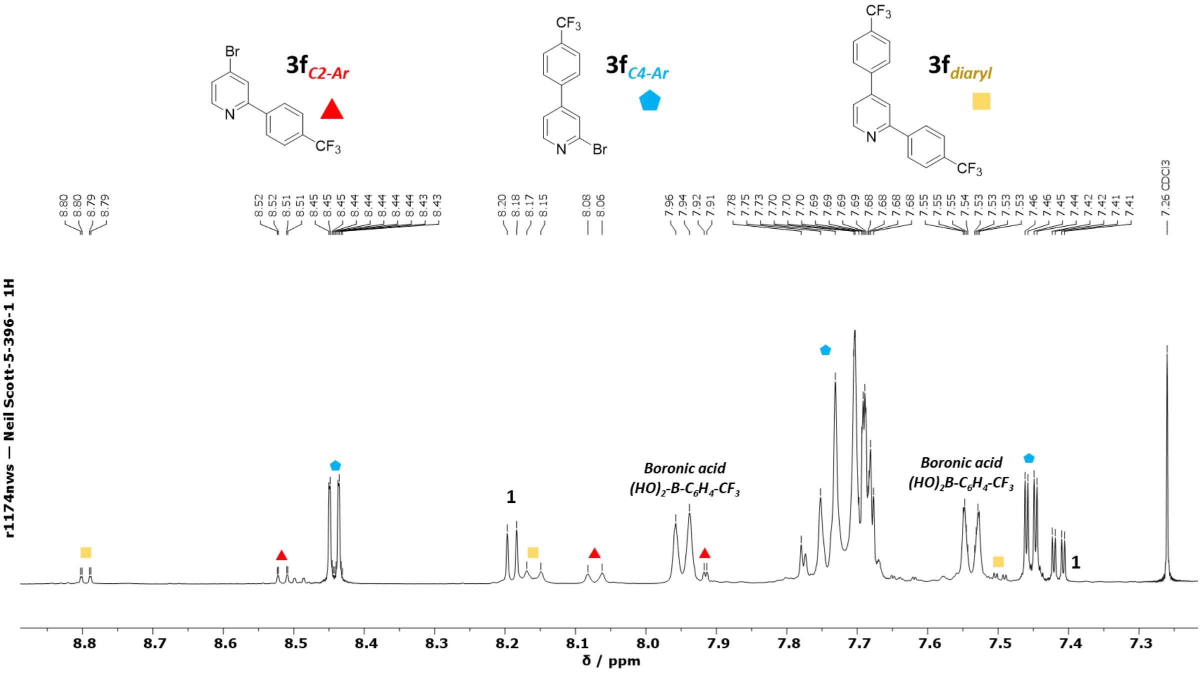

Figure S12 ^{1}H NMR spectrum (500 MHz, $CDCl_3$) showing 3f$_{C4\text{-}Ar}$, 3f$_{C2\text{-}Ar}$ and 3f$_{diaryl}$ compound identification from a crude reaction mixture. (note: small differences in ^{1}H chemical shift from authenticated products are seen here).

2-Bromo-4-(4-trifluoromethylphenyl)pyridine (3f$_{C4\text{-}Ar}$)[12]

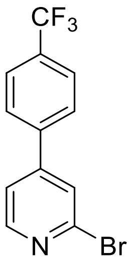

Compound characterisation data agrees with that previously reported in literature.[12] It was isolated from a reaction mixture (Section 2.5, **Pd_3Cl_2**) using flash chromatography (SiO_2) with a hexane/EtOAc (98:2 v/v) solvent system, which was run with a gradient starting from neat hexane. Yield from reaction catalysed by **Pd_3Cl_2** = 141.3 mg (47%). Appeared as a colorless powder. Melting point – 115.0-117 °C (123.5-124.5 °C lit.).[12]

^{1}H NMR (500 MHz, Chloroform-*d*) δ 8.46 (dd, *J* = 5.2, 0.6 Hz, 1H, *pyridyl*-C$_6$–H), 7.74 – 7.79 (m, 2H, *aryl*), 7.73 – 7.66 ({m, 2H, *aryl*} + {*pyridyl*-C$_3$–H}), 7.47 (dd, *J* = 5.2, 1.6 Hz, 1H, *pyridyl*-C$_5$–H) ppm.

^{13}C NMR (126 MHz, Chloroform-*d*) δ 150.4 (*pyridyl*-C–H), 149.5 (C), 142.9 (C), 140.0 (C), 131.4 (q, $^{2}J_{C-F}$ = 33.0 Hz *aryl*-C_4'), 127.3 (*aryl*-C–H) 126.0 (q, $^{3}J_{C-F}$ = 3.7 Hz, *aryl*-C_3'–H), 125.8 (C–H) , 123.6 (q, *J* = 272.4 Hz; $^{1}J_{C-F}$ *C*F_3) 120.7 (C–H) ppm.

^{19}F NMR (376 MHz, Chloroform-d) δ –62.65 ppm.

IR (ν/cm^{-1}, ATR): 3019(w), 2981(w), 2831(w), 1608(m), 1585(m), 1515(s), 1457(m), 1429.42(m), 1374.92(m), 1292.08(m), 1184.00 (m), 1017.76(m), 814.70(s), 574.42(m), 567.88(m), 519.79(m).

HRMS ESI-MS m/z = 301.9773 [M+H]$^+$: $C_{12}H_8{}^{79}BrF_3N$ requires 301.9787.

Elemental analysis (% CHN), calculated for $C_{12}H_7BrF_3N$; C 47.71; H, 2.34; N, 4.64 Found: C, 48.09; H, 2.63; N, 4.12.

R_f (TLC, Hexane/EtOAc, 98:2 {v/v}) = 0.12.

XRD analysis (ijsf1803; CCDC 2060853):

Crystal was grown by vapour diffusion of pentane on a dichloromethane solution of 3f$_{C4\text{-}Ar}$.

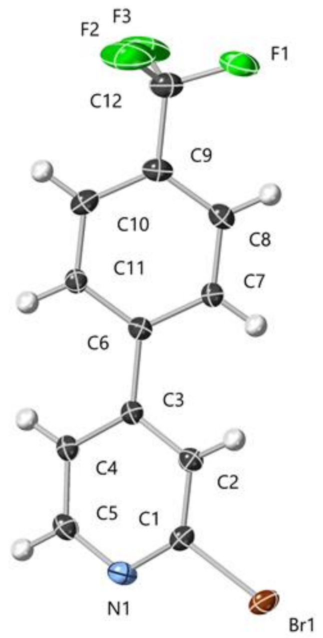

Figure S13 Structure obtained from X-ray diffraction analysis of a single crystal of 2-bromo, 4-(4-trifluoromethylphenyl)pyridine (3f$_{C4\text{-}Ar}$).

The exact crystal run (by XRD) was subjected to ^{1}H NMR spectroscopic analysis (700 MHz) which matched data of that of the isolated material (*vide supra*). This confirms conclusively the identity of the NMR characterised species as the C4-arylated product. Thus, X-ray crystallographic analysis shows distortion of the pyridine ring (Table S9 & Table S10).

Table S9 Analysis of selected bond angles from XRD analysis of a crystal of 3f$_{C4\text{-}Ar}$

Angle sweep	Θ /°	deviation from 120 degrees
C(5)–N(1)–C(1)	115.21	4.79
N(1)–C(1)–C(2)	126.02	-6.02
N(1)–C(5)–C(4)	124.25	-4.25
C(1)–C(2)–C(2)	118.01	1.99
C(5)–C(4)–C(2)	119.17	0.83
C(2)–C(4)–C(4)	117.3	2.7

Table S10 Analysis of selected bond lengths from XRD analysis of a crystal of 3f$_{4\text{-}Ar}$

Bond	Bond length /Å
N(1)–C(1)	1.323(3)
N(1)–C(5)	1.347(3)
C(1)–C(2)	1.381(3)
C(5)–C(4)	1.378(3)
C(2)–C(4)	1.396(3)
C(4)–C(2)	1.398(3)
C(1)–Br(1)	1.906(2)

See Appendix 4 for further X-Ray Crystallographic Data (ijsf1803).

4-Bromo-2-(4-trifluoromethylphenylphenyl)pyridine (3f$_{C2\text{-}Ar}$)

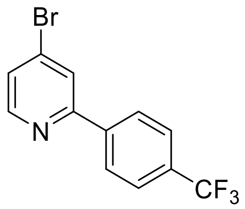

To our knowledge, data for this compound was not previously reported within literature. It was isolated from the crude reaction mixture, catalysed by **Pd_3Cl_2** (Section 2.5) using PTLC (SiO_2) with a hexane/EtOAc (98:2) solvent system. Yield from reaction catalysed by **Pd_3Cl_2** = 3.7 mg (1 %). Multiple solvent runs were needed to allow separation from the starting material. Appeared as a colorless powder.

^{1}H NMR (400 MHz, Chloroform-*d*) 8.54 (dd, *J* = 5.2, 0.6 Hz, 1H; *pyridyl*-C_6-H), 8.07 – 8.13 (m, 2H, *aryl*), 7.94 (dd, *J* = 1.8, 0.6 Hz, 1H, *pyridyl*-C_3–H), 7.71 – 7.76 (m, 2H, *aryl*), 7.47 (dd, *J* = 5.2, 1.8 Hz, 1H, *pyridyl*-C_5–H).

^{13}C NMR (101 MHz, Chloroform-*d*) δ 157.4 (C), 150.7 (C–H), 141.4 (C), 133.9 (C), 131.6 (q, $^{2}J_{C\text{-}F}$ = 32.6 Hz, C$_4$'), 127.5 (C–H), 126.3 (C–H), 126.0 (q, $^{3}J_{C\text{-}F}$ = 3.8 Hz, C$_3$'–H), 124.4 (C–H), 124.2 (q, $^{1}J_{C\text{-}F}$ = 272.2, *C*F$_3$).

^{19}F NMR (376 MHz, Chloroform-d) δ –62.56 ppm.

HRMS ESI-MS m/z = 301.9773 [M+H]$^+$: $C_{12}H_8{}^{79}BrF_3N$ requires 301.9787.

IR (ν/cm^{-1}, ATR): 3071 (w), 3054 (w), 2934 (w), 1616 (w), 1566 (m), 1548 (m), 1466 (m), 1376 (m), 1323 (s), 1263 (m), 1169 (s), 1110 (s), 1091 (s), 1070 (s), 1042 (m), 1011 (m), 851 (s), 824 (s), 754 (m), 687 (m), 606 (s), 434 (s).

Elemental analysis (% CHN), calculated for $C_{12}H_7{}^{79}BrF_3N$; C 47.71; H, 2.34; N, 4.64 Found: C, 47.15; H, 2.37; N, 4.56.

R$_f$ (TLC, Hexane/EtOAc, 98:2 {v/v}) = 0.33.

Bis-2,4-(4-trifluoromethylphenyl)pyridine (3f*diaryl*)[7]

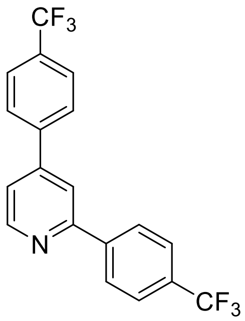

Identified in the crude reaction mixture by comparison to literature reported ^{1}H NMR spectroscopic data (*see above*).[7]

^{1}H NMR (400 MHz, Chloroform-*d*) δ 8.80 (dd, *J* = 5.1, 0.8 Hz, 1H), 8.18 – 8.14 (m, 2H), 7.50 (dd, *J* = 5.1, 1.7 Hz, 1H) ppm. {partial data, from crude ^{1}H NMR}

^{19}F NMR (376 MHz, Chloroform-*d*) δ -62.47, -62.55 ppm. {partial data, from crude ^{1}H, ^{19}F NMR}

HRMS ESI-MS m/z = 368.0858 [M+H]$^+$: $C_{19}H_{12}F_6N$ requires 368.0868.

3.2. Synthesis of Tris-imidazolium Tribromide (6)

3.2.1. Step 1 – Synthesis of 1,3,5-Tris(bromomethyl)-2,4,6-triethyl-benzene

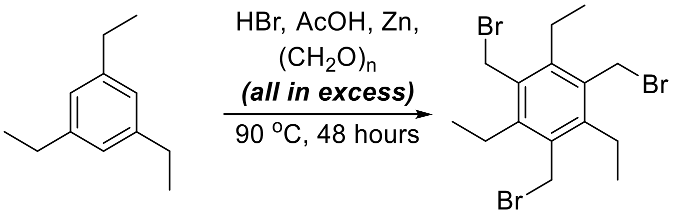

Adapted procedure[13-14]**:** An oven dried round bottomed flask, fitted with a water condenser, was charged with Zn powder (2.5 g, 38 mmol) and AcOH (25 mL, 436.5 mmol). HBr in AcOH (33% wt, 25 mL) was added over a 30-minute period with magnetic stirring until completely dissolved, resulting in a deep orange solution. 1,3,5-Triethylbenzene (5 g, 31 mmol), paraformaldehyde (10 g, 333 mmol, 10.7 eq.) and HBr in AcOH (33% wt, 74 mL, 1238 mmol, 39.9 equiv.) were then added and the solution heated to 90 °C for 48 hours, resulting in a dark brown solution. After this time, the solution was left to cool slowly to room temperature with magnetic stirring, resulting in precipitation of a yellow powder over the course of 2 hours. The solid was filtered, washed with H_2O (3 × 50 mL) and allowed to dry under vacuum (~7.5 × 10^{-3} mmHg) for 24 hours, yielding a pale-yellow solid (7.83 g {57%}). The data obtained matched with the literature.[13-14] Melting point – 171.0-173.0 °C.[14]

^{1}H NMR (400 MHz, 8 scans, Chloroform-*d*) δ 4.57 (s, 6H, CH_2CH$_3$), 2.93 (q, *J* = 7.7 Hz, 6H), 1.33 (t, *J* = 7.7 Hz, 9H, CH$_2$CH_3) ppm.

^{13}C NMR (101 MHz, Chloroform-*d*) δ 145.1 (Ar), 132.7 (Ar), 28.7 (CH$_2$Br), 22.8 (CH$_2$), 15.7 (CH$_3$) ppm.

IR (ν, ATR, solid state, cm^{-1}): 2966 (s), 2931 (m), 2901 (m), 2871 (m), 1714 (w, broad), 1571 (m), 1452 (s), 1204 (s), 1041 (m), 957 (m), 898 (m), 764 (s), 705 (s), 587 (s), 502 (s).

EI-MS m/z = 437.91699 $[M]^{\cdot+}$: $C_{15}H_{21}{}^{79}Br_3$ requires 437.91934.

3.2.2. Step 2 – Synthesis of 1,3,5-Tris(methyl(2-methylimidazole))-2,4,6-triethylbenzene

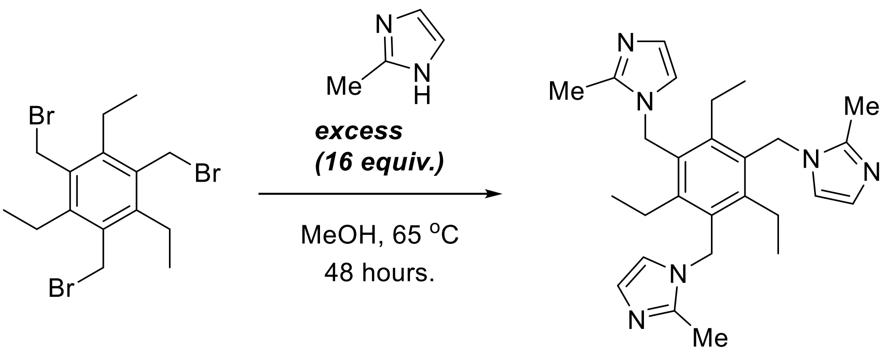

Adapted Procedure[15]: To a mixture of 1,3,5-tris(bromomethyl)-2,4,6-triethylbenzene (3 g, 6.83 mmol), 2-methylimidazole (9.05 g, 8.3 mL, 110.2 mmol, 16 equiv) and MeOH (75 mL) was added. The mixture was stirred at reflux (65 °C) for 48 hours. After this time, the solvent was removed in vacuum yielding a yellow, oily solid. Solids were dissolved in water (20 mL) and $NaHCO_3$ (aq. sat.) (20 mL) was added. The organic layer was extracted with CH_2Cl_2 (3 × 30 mL) and the combined organic layer was concentrated in vacuum to yield a yellow oil. A precipitate subsequently began to appear over time at room temperature. For purification by crystallisation, the precipitate was taken up in the minimum hot water under reflux conditions and allowed to cool slowly to room temperature, before being stored in a fridge overnight to aid further crystallisation. The crude product was filtered off and washed with diethyl ether (3 × 10 mL) to give needle-like crystals 1.54 g, 51%.

^{1}H NMR (400 MHz, Chloroform-*d*) δ 6.83 (s, 3H, NC*H*CHN), 6.19 (s, 3H, NCHC*H*N), 5.02 (s, 6H, ArCH_2N), 2.58 (q, *J* = 7.6 Hz, 6H, CH_2CH$_3$), 2.52 (s, 9H, NC(CH_3)N), 1.00 (t, *J* = 7.6 Hz, 9H, CH$_2$CH_3).

^{13}C NMR (101 MHz, DMSO-d_6) 145.2 (Ar), 144.5 (N*C*(CH$_3$)N), 131.4 (Ar), 127.0 (N*C*HCHN), 117.7 (NCH*C*HN), 43.7 (*C*H$_2$N), 23.4(*C*H$_2$Ar), 15.4 (*C*H$_3$), 13.5 (*C*H$_3$) ppm.

^{13}C NMR DEPT 135 (101 MHz, DMSO-d_6) δ 127.0, 117.7, 43.7 (-ve, sp^3 CH$_2$), 23.4 (-ve, sp^3 CH$_2$), 15.4, 13.5 ppm. Disappearance of quaternary signals at δ 145.2, 144.5, 131.4 ppm.

HRMS ESI-MS m/z = 445.3072[M+H]$^+$, APCI-MS m/z : 445.309087 [M+H]$^+$: $C_{27}H_{37}N_6$ requires 445.3080.

IR (ν, ATR, solid state, cm^{-1}): 3495 (m, broad), 3179 (m, very broad), 2966 (m), 2926 (m), 2871 (w), 1684 (m, broad), 1531 (m), 1496 (m), 1452 (s), 1417 (s), 1383 (s), 1259 (s), 1135 (s), 987 (s), 729 (s), 680 (s), 591 (s).

*XRD analysis for **6** (ijsf2006):*

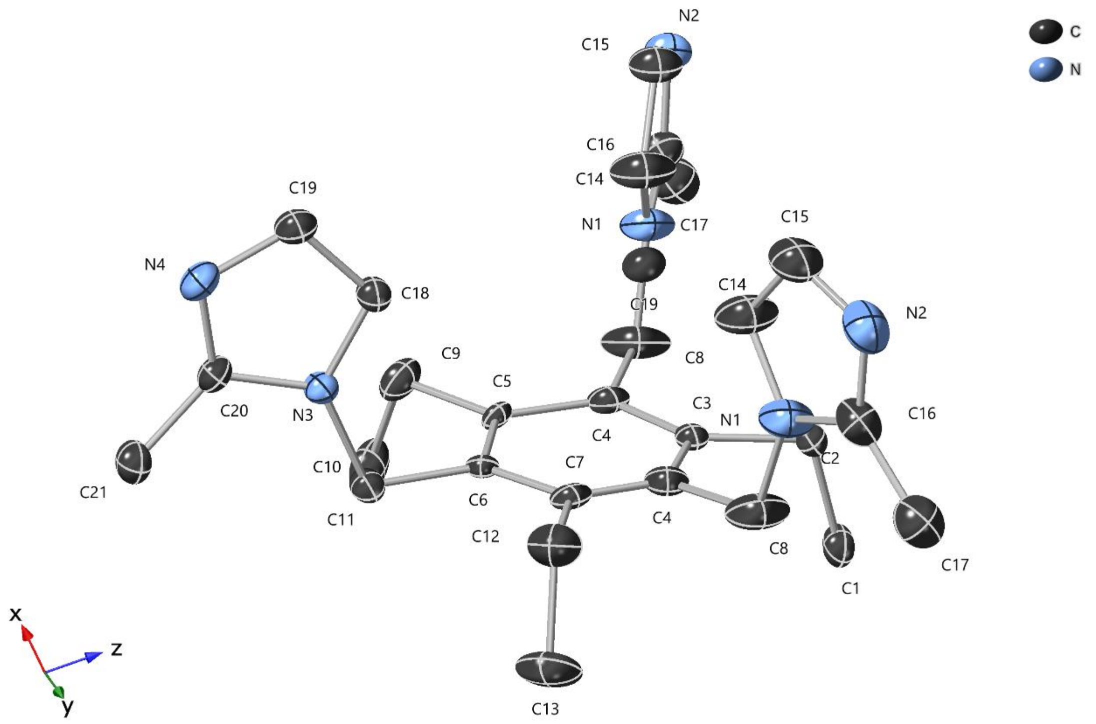

Figure S14 Structure of 1,3,5-tris(methyl(2-methylimidazole))-2,4,6-triethylbenzene, obtained *via* X-ray diffraction of a single crystal. Thermal ellipsoids are set at 50% probability, H_2O molecules of crystallisation and H atoms are omitted for clarity) Selected bond lengths /Å: C4-C5 = 1.505(12); C14-N1 = 1.381(2); N3-C20 = 1.361(3); C16-N1 = 1.356(2); C8-N1 = 1.4777(19); N2-C11 = 1.377(3). Selected bond angles /° C5-C7-C8 = 110.828(6); C6-C14A-N3A = 112.058(); C4-C9-N1 = 110.40(15) {ijsf2006; CCDC 2060856).

3.2.3. Step 3 – Synthesis of 2-Methyl-3-(3-methylbutyl)-1-([2,4,6-triethyl-3,5-bis(([2-methyl-3-(3-methylbutyl)-1H-imidazol-3-ium-1-yl]methyl))phenyl]methyl)-1H-imidazol-3-ium (6)

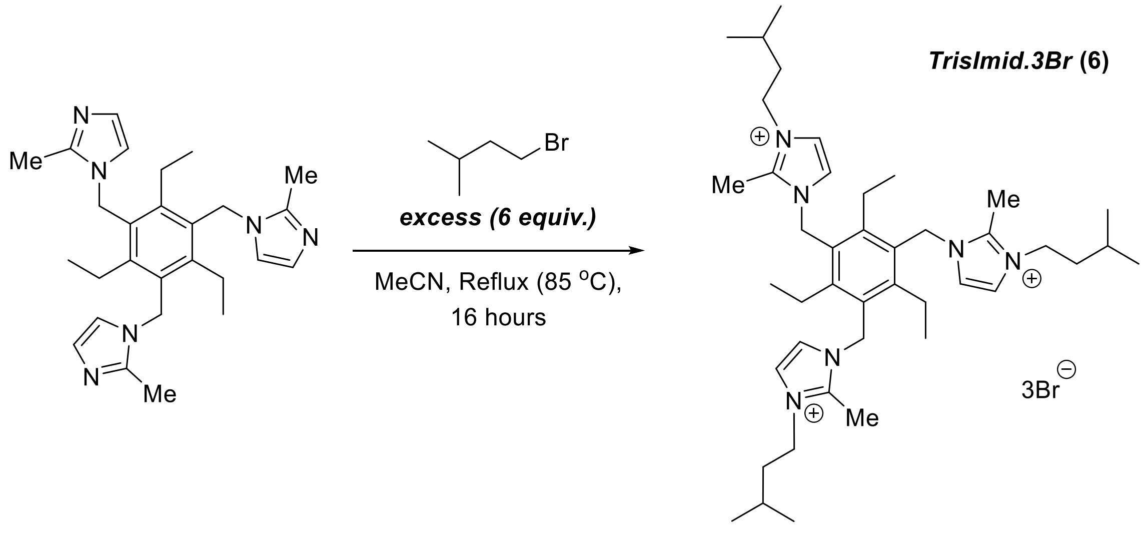

Adapted procedure[16]: A stirred solution of 1,3,5-tris(methyl(2-methylimidazole))-2,4,6-triethylbenzene (1.5 g, 3.375 mmol, 1 equiv.) and 2-methyl-4-bromobutane (2.55 mL, 20.25 mmol, 6 equiv.) in MeCN (60 mL) was heated to reflux overnight. The solvent was partially evaporated, and a colorless, powdery precipitate appeared after addition of diethyl ether. The resulting solid was filtered and washed with diethyl ether to yield the title compound which appeared as a colorless powder and was subsequently dried under high vacuum (~7.5 × 10^{-3} mmHg) and stored under nitrogen, 2.09 g yield (69%). Note: colorless powder product was very hygroscopic, reversibly forming a transparent gel when exposed to atmospheric H_2O. It was sealed under an N_2 atmosphere to prevent absorption of moisture. Melting point – 138.2-139.5 °C

^{1}H NMR (400 MHz, 64 scans, $CDCl_3$) δ 7.50 (d, *J* = 2.2 Hz, 3H, N*CH*CHN), 7.26 (d, *J* = 2.2 Hz, 3H, NCH*CH*N), 5.51 (s, 6H, Ar*CH₂*N), 4.07 (t, *J* = 6.0 Hz, 6H, N^+CH_2), 3.05 (s, 9H, NC(CH_3)N), 2.58 (q, *J* = 7.5 Hz, 6H, CH_2CH_3), 1.75 – 1.59 (m, 9H, with broad H_2O overlapping, Alkyl), 1.12 (t, *J* = 7.5 Hz, 9H, CH_3CH_2), 0.96 (d, *J* = 6.4 Hz, 18H, CH(CH_3)$_2$ ppm.
^{13}C NMR (101 MHz, 1024 scans, $CDCl_3$) δ 148.7 (Ar), 143.3 (N*C*(CH_3)N), 128.3 (Ar), 122.4 (N*C*HCHN), 121.9 (NCH*C*HN), 47.4 (*C*H_2N), 47.2 (N^+*C*H_2), 38.4 (Alk), 25.7 (Alk), 24.3 (*C*H_2Ar), 22.3 (Alk), 16.1 (Alk), 12.0 (Alk) ppm.
HRMS ESI-MS m/z = 219.1857 $[M+H]^{3+}$, $C_{42}H_{69}N_6]^{3+}$ requires 219.1856.
IR (ν, ATR, solid state, cm^{-1}): 2956.96(br, s), 2970.81 (m), 1575.52(m), 2523.01 (m), 1505.04 (m), 1463.95 (s), 1434.50 (s), 1388.32 (m), 1367.62 (m), 1249.55 (m), 1193.72 (s), 1174.52 (m), 1074.19 (w), 1041.33 (w), 921.49 (w), 767.73 (br, s), 668.22 (m).
Elemental analysis (% CHN), calculated for $C_{42}H_{72}Br_3N_6O_{1.5}$ (as the hydrate, modelled containing 1.5 H_2O molecules), C 54.22; H, 7.72; N, 9.20 Found: C, 54.14; H, 7.75; N, 9.36.

3.2.4. Assessment of the effects of tris-imid.3Br 6 in a SMCC reaction between 1 and 2b

Tris-imidazolium tribromide **6** was applied to the benchmark SMCC conditions (see conditions and results below), catalyzed by our optimized catalyst precursor system, $Pd(OAc)_2$/1 PPh_3. We noted a marked rise in site-selectivity at **1**, exhibiting a **3b**$_{C4-Ar}$:**3b**$_{C2-Ar}$ ratio of 17.6:1, with a relatively low formation of **3b**$_{diaryl}$ product.

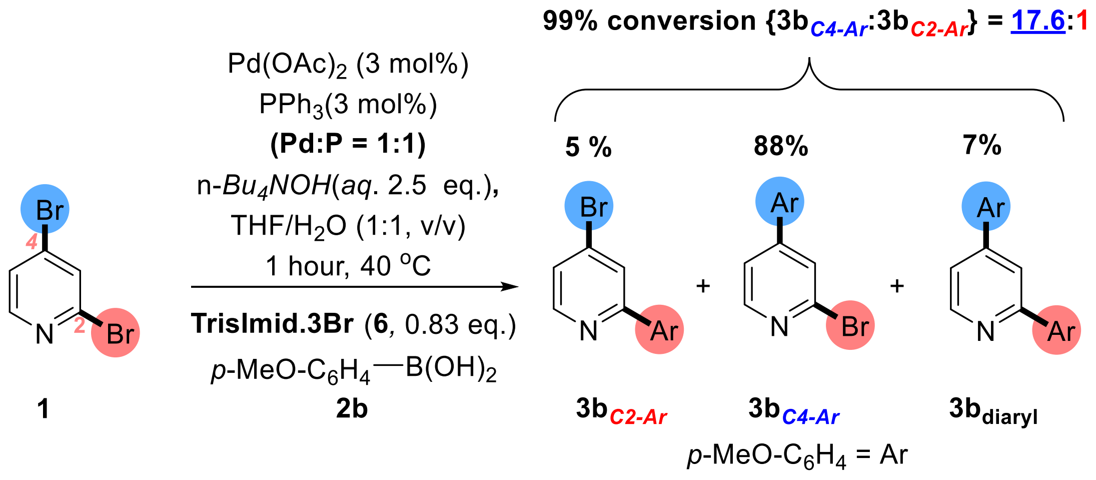

3.3. Synthetic Utility Employing a C4-Site-Selective SMCC Reaction First, Followed by an Ullmann Coupling

4[4-anisyl, 2-pyridinyl}oxy]-benzeneacetic acid methyl ester

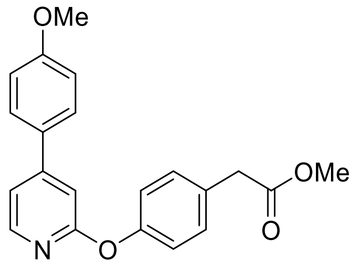

Synthesised in 2 steps from 2,4-dibromopyridine **1**.

Step 1 *Site-selective SMCC at 2,4-dibromopyridine*: 2-bromo,4-(4-anisyl)pyridine (**3b$_{4\text{-Ar}}$**) was synthesised using the model reaction conditions $Pd_3(OAc)_6$/3PPh_3 as the catalyst system (Section 2.1). An oven-dried Schlenk tube (Flask 1) charged with $Pd_3(OAc)_6$ (23.6 mg, 0.03 mmol {3 mol%/Pd}) and triphenylphosphine (23.6 mg, 0.03 mmol) was evacuated and backfilled with N_2. THF (2.5 mL; dry, degassed) was added *via* a syringe and the immediately formed greenish-yellow suspension was stirred in an oil bath which was pre-heated to 40 °C. Another oven-dried Schlenk tube (*Flask 2*) was charged with 2,4-dibromopyridine **1** (710 mg, 3.00 mmol, 1 equiv.) and *p*-anisylboronic acid **2b** (479 mg, 3.15 mmol) and subsequently evacuated and backfilled with N_2. After 30 minutes of stirring, the contents of *Flask 1* were transferred into *Flask 2 via* a cannula and the resulting mixture was stirred for 5 mins in order to thermally equilibrate at 40 °C. Aqueous *tetra-N-butylammonium* hydroxide (2.5 mL, 1.0 M, degassed) was then added (t_0) to commence the reaction. The reaction was stirred overnight at 40 °C. After the reaction time the reaction solution was quenched using a of NH_4Cl (sat. aq.). The organics were extracted using EtOAc (4 × 10 mL) before being combined and dried over $MgSO_4$, filtered and subsequently concentrated *in vacuo*. The resulting residue, which appeared as a reddish-brown oil was adsorbed to silica and purified by flash chromatography (SiO_2) using a Hexane or petroleum ether/EtOAc (85:15) solvent system to give the product which appeared as a colourless oil (550 mg, 69%).

Step 2 Ullman Coupling at **3b$_{4\text{-Ar}}$**: Adapted from a procedure published by Zhao *et al.*[17] A Schlenk tube, charged with CuI (18.6 mg, 0.1 mmol) and TMEDA (11.4 mg, 0.1 mmol) was evacuated and backfilled (three times), before being dissolved in dry, degassed toluene (5 mL) and subsequently stirred for 30 min at 23 °C. Cesium carbonate (638.6 mg, 2 mmol) and methyl (4- hydroxy-phenyl)-acetate (166.2 mg, 1.00 mmol) were added into the mixture (as solids) and stirred at room temperature for another 4 h. Finally, solution of 2-bromo,4-(4-anisyl)-pyridine **3b$_{4\text{-Ar}}$** (200 mg, 0.758 mmol) in dry toluene (3 mL) was added (total volume = 8 mL => 0.125 M {respect to pyridine substrate}). The reaction mixture was placed in oil-bath which was preheated to 110 °C (refluxed, under a sealed system, with a cold

finger) under a nitrogen atmosphere for 24 h. Reaction progress was monitored by TLC (85:15 v/v Pet ether/ EtOAc). On completion, the reaction mixture was cooled to room temperature. The solvent was evaporated under vacuum (Schlenk) before the residue was dissolved in CH_2Cl_2 (20 mL), filtered (sinter funnel) and concentrated in vacuum to afford the crude product which appeared as a colorless powder, 264 mg (53%) yield of isolated product (Step 2). Melting point – 103.2-104.0 ^{o}C.

^{1}H NMR (400 MHz, Chloroform-*d*) δ 8.19 (d, *J* = 5.3 Hz, 1H, *pyridyl*-C_6–H), 7.59 (d, *J* = 8.8 Hz, 2H, *aryl*), 7.33 (d, *J* = 8.5 Hz, 2H, *aryl*), 7.19 (dd, *J* = 5.3, 1.6 Hz, 1H, *pyridyl*-C_5–H), 7.13 (d, *J* = 8.5 Hz, 2H, *aryl*), 7.09 (d, *J* = 1.5 Hz, 1H, *pyridyl*-C_5–H), 6.98 (d, *J* = 8.8 Hz, 2H), 3.87 (s, 3H, CH_3), 3.71 (s, 3H, CH_3), 3.64 (s, CH_2).

^{13}C NMR (101 MHz, Chloroform-*d*) δ 172.6 (C), 165.0 (C), 161.2 (C), 154.0 (C), 152.3 (C), 148.5 (C–H), 130.7 (C–H), 130.2 (C–H), 128.8 (C–H), 121.8 (C–H), 117.1 (C–H), 115.1 (C–H), 109.1 (C–H), 55.5 (CH_{aliph}), 52.2 (CH_{aliph}), 40.7 (CH_{aliph}).

IR (v/ cm^{-1}, ATR): 3038 (w, C–H), 2953 (w, C–H), 2895 (w, C–H), 2842 (w,C–H), 1734 (m, C=O stretch), 1601 (m), 1393.9 (m), 1217 (m), 1164 (br. m), 1028 (m), 812 (s).

HRMS ESI-MS [M+H] m/z = 350.1387: $C_{21}H_{20}NO_4$ requires 350.1391.

R_f (TLC, PET/EtOAc, 85:15 {v/v}) = 0.15.

4. X-Ray Crystallography

Diffraction data were collected at 110 K on an Oxford Diffraction SuperNova diffractometer with Cu-K_{α} radiation (λ = 1.54184 Å using an EOS CCD camera. The crystal was cooled with an Oxford Instruments Cryojet. Diffractometer control, data collection, initial unit cell determination, frame integration and unit-cell refinement was carried out with "Crysalis".[18] Face-indexed absorption corrections were applied using spherical harmonics, implemented in SCALE3 ABSPACK scaling algorithm.[19] OLEX2[20] was used for overall structure solution and refinement . Within OLEX2, the algorithm used for structure solution was "ShelXT dual-space".[21] Refinement was carried out by full-matrix least-squares used the SHELXL-97[22] algorithm within OLEX2.[20] All non-hydrogen atoms were refined anisotropically. Crystalmaker® software was used to visualise the structures as well as generating the figures presented herein.

ijsf1805: Bromo(4-bromo-C2-pyridinyl)bis(triphenylphosphine) ($OA_{C2\text{-}Br}$)

Refinement Special Details

See Figure S4 for single crystal structure image. The asymmetric unit contained half of the complex plus 1.25 dichloromethanes (in 3 discrete positions). All were reflected about a central mirror plane parallel to the ac plane. In addition, the quarter dichloromethane was also at an inversion centre. The carbon of the quarter dichloromethane was disordered over two sites due to the centre of inversion. The complex was disordered with the bromopyridyl ring being modelled in two positions with refined occupancies of 0.858:0.142(2). The ring of the minor form was constrained to be a regular hexagon with bond-lengths of 1.39 Å. Several of the atoms of the bromopyridyl were restrained to be in the same plane. Closely proximal atoms in the bromopyridyl ring were constrained to have the same ADP, namely C1 & C1A, N1 & C2A, C5 & C3A, C3, C4A & C5A and C2 & N1A.

Table S11 X-Ray Diffraction Data for $OA_{C2\text{-}Br}$

Identification code	ijsf1805 (CCDC 2060854)
Empirical formula	$C_{43.5}H_{38}Br_2Cl_5NP_2Pd$
Formula weight	1080.16
Temperature/K	110.00(10)
Crystal system	Monoclinic
Space group	I2/m
a/Å	17.3326(4)
b/Å	14.8308(3)
c/Å	18.9185(4)
α/°	90
β/°	116.862(3)
γ/°	90
Volume/Å^3	4338.4(2)
Z	4
ρ_{calc}g/cm^3	1.654
μ/mm^{-1}	9.392
F(000)	2148.0
Crystal size/mm^3	0.205 × 0.125 × 0.025
Radiation	CuKα (λ = 1.54184)
2Θ range for data collection/°	7.936 to 134.16
Index ranges	-19 ≤ h ≤ 20, -17 ≤ k ≤ 13, -22 ≤ l ≤ 20
Reflections collected	16229
Independent reflections	4047 [R_{int} = 0.0235, R_{sigma} = 0.0185]
Data/restraints/parameters	4047/6/285
Goodness-of-fit on F^2	1.164
Final R indexes [I>=2σ (I)]	R_1 = 0.0373, wR_2 = 0.0886
Final R indexes [all data]	R_1 = 0.0386, wR_2 = 0.0893
Largest diff. peak/hole / e Å^{-3}	0.85/-1.24

2-Bromo-4-(4-trifluoromethylphenyl)pyridine(3f$_{C4\text{-}Ar}$)

Refinement Special Details

See Figure S13 for single crystal structure image. The fluorine atoms within the CF_3 group were disordered and modelled in two positions with refined occupancies of 0.820:0.180(18), the ADPs of the fluorine atoms were restrained to be approximately isotropic.

Table S12 X-Ray Diffraction Data for 2-bromo-4-(4-trifluoromethylphenyl)pyridine (3f$_{C4\text{-}Ar}$)

Identification code	ijsf1803 (CCDC 2060853)
Empirical formula	$C_{12}H_7BrF_3N$
Formula weight	302.10
Temperature/K	110.05(10)
Crystal system	orthorhombic
Space group	Pbca
a/Å	11.6735(2)
b/Å	6.98840(10)
c/Å	27.1656(4)
α/°	90
β/°	90
γ/°	90
Volume/Å^3	2216.14(6)
Z	8
ρ_{calc}g/cm^3	1.811
μ/mm^{-1}	5.253
F(000)	1184.0
Crystal size/mm^3	0.307 × 0.176 × 0.043
Radiation	CuKα (λ = 1.54184)
2Θ range for data collection/°	9.992 to 142.21
Index ranges	-14 ≤ h ≤ 13, -8 ≤ k ≤ 8, -33 ≤ l ≤ 26
Reflections collected	10677
Independent reflections	2118 [R_{int} = 0.0343, R_{sigma} = 0.0200]
Data/restraints/parameters	2118/36/182
Goodness-of-fit on F^2	1.056
Final R indexes [I>=2σ (I)]	R_1 = 0.0283, wR_2 = 0.0756
Final R indexes [all data]	R_1 = 0.0311, wR_2 = 0.0782
Largest diff. peak/hole / e Å^{-3}	0.35/-0.39

ijsf1908: bis[μ-(acetato)]bis(acetato)bis(triphenylphosphine)dipalladium ([$Pd^{II}(\mu_2$-OAc)(κ-OAc)(PPh_3)]$_2$) (4)

See Section 2.9 for details on how the compound was synthesised and how the crystal was isolated.

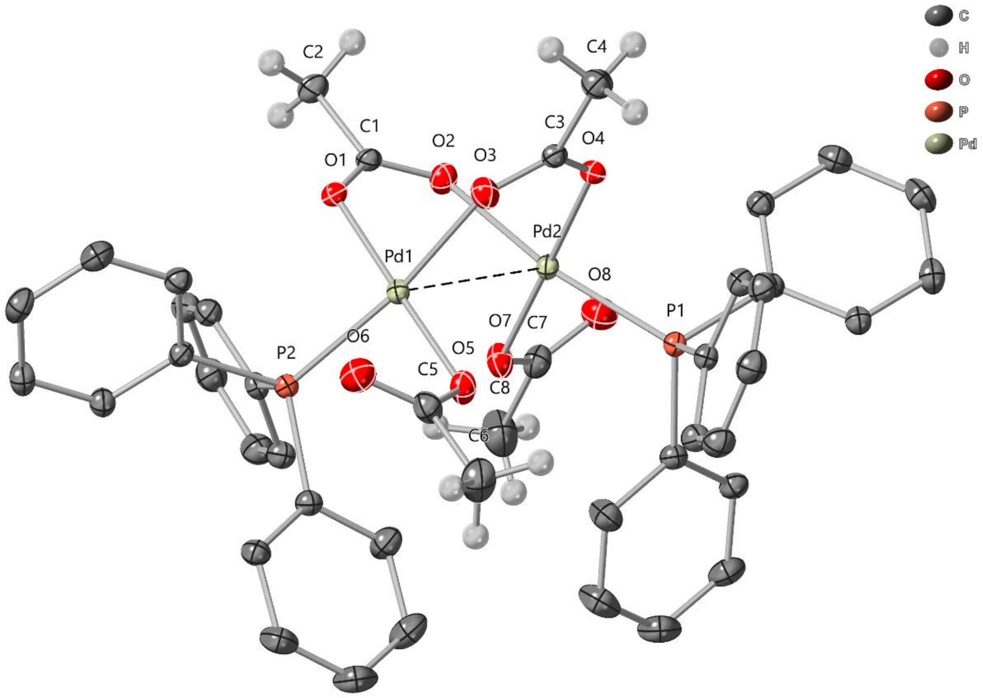

Figure S15 Structure obtained by X-ray diffraction analysis of a single crystal of [$Pd^{II}(\mu_2$-OAc)(κ-OAc)(PPh_3)]$_2$ (4). Thermal ellipsoids were set at 50% probability. Selected interatomic distances /Å: Pd1–P2 = 2.2329(7), Pd1–O1 = 2.0209(18), Pd1–O3 = 2.1062(19), Pd1–O5 = 1.999(2), Pd1–Pd2 = 3.0115(4). Selected bond angles /°: P2–Pd1–O1 = 88.62(5), O1–Pd1–O3 = 89.61(8) P2–Pd1–O5 = 92.11(6), O3–Pd1–O5 = 89.83(8). Torsion angles /°: O5–Pd1–Pd2–O7 = 89.766, O5–Pd1–Pd2–P1 = -4.867, P2–Pd1–Pd1–P2 = -99.500 {ijsf1908; CCDC 2060855}.

Refinement Special Details

The asymmetric unit contained half a molecule, the other half being generated by a mirror plane.

Table S13 X-Ray Diffraction Data for ([Pd^{II}(μ_2-OAc)(κ-OAc)(PPh_3)]$_2$) (4)

Identification code	ijsf1908
Empirical formula	$C_{44}H_{42}O_8P_2Pd_2$
Formula weight	973.51
Temperature/K	110.05(10)
Crystal system	monoclinic
Space group	C2/c
a/Å	20.9152(4)
b/Å	9.6486(2)
c/Å	22.0620(4)
α/°	90
β/°	113.600(2)
γ/°	90
Volume/Å^3	4079.80(15)
Z	4
ρ_{calc}g/cm^3	1.585
μ/mm^{-1}	8.292
F(000)	1968.0
Crystal size/mm^3	0.201 × 0.151 × 0.088
Radiation	CuKα (λ = 1.54184)
2Θ range for data collection/°	8.748 to 134.142
Index ranges	-24 ≤ h ≤ 23, -11 ≤ k ≤ 11, -26 ≤ l ≤ 23
Reflections collected	7133
Independent reflections	3635 [R_{int} = 0.0200, R_{sigma} = 0.0234]
Data/restraints/parameters	3635/0/255
Goodness-of-fit on F^2	1.058
Final R indexes [I>=2σ (I)]	R_1 = 0.0255, wR_2 = 0.0659
Final R indexes [all data]	R_1 = 0.0279, wR_2 = 0.0680
Largest diff. peak/hole / e Å^{-3}	0.67/-0.59

ijsf2006 2-Methyl-3-(3-methylbutyl)-1-([2,4,6-triethyl-3,5-bis(([2-methyl-3-(3-methylbutyl)-1H-imidazol-3-ium-1-yl]methyl))phenyl]methyl)-1H-imidazol-3-ium (6, ijsf2006)

Refinement Special Details

The compound was prepared and crystallised as detailed in Section 3.2. See Figure S14 for single crystal structure image. The structure was disordered about a mirror plane parallel to the ac plane at b=0.25. In the central ring, C3 and C4 occupied a common site for both conformations.

Table S14: X-Ray Diffraction Data for 3,5-tris(methyl(2-methylimidazole))-2,4,6-triethylbenzene.

**Identification code**	**ijsf2006 (CCDC 2060856)**
**Empirical formula**	$C_{27}H_{42}N_6O_3$
**Formula weight**	498.66
**Temperature/K**	109.8(6)
**Crystal system**	monoclinic
**Space group**	$P2_1/m$
**a/Å**	7.7470(4)
**b/Å**	16.6589(11)
**c/Å**	10.9761(6)
**α/°**	90
**β/°**	100.387(5)
**γ/°**	90
**Volume/Å^3**	1393.33(14)
**Z**	2
**ρ_{calc}g/cm^3**	1.189
**μ/mm^{-1}**	0.632
**F(000)**	540.0
**Crystal size/mm^3**	0.216 × 0.195 × 0.092
**Radiation**	Cu Kα (λ = 1.54184)
**2Θ range for data collection/°**	8.19 to 134.16
**Index ranges**	-6 ≤ h ≤ 9, -19 ≤ k ≤ 17, -13 ≤ l ≤ 12
**Reflections collected**	4940
**Independent reflections**	2573 [R_{int} = 0.0239, R_{sigma} = 0.0348]

**Data/restraints/parameters**	2573/0/253
**Goodness-of-fit on F^2**	1.039
**Final R indexes [I>=2σ (I)]**	R_1 = 0.0423, wR_2 = 0.0990
**Final R indexes [all data]**	R_1 = 0.0559, wR_2 = 0.1082
**Largest diff. peak/hole / e $Å^{-3}$**	0.18/-0.16

5. NMR Spectral Data for Isolated Organic Compounds

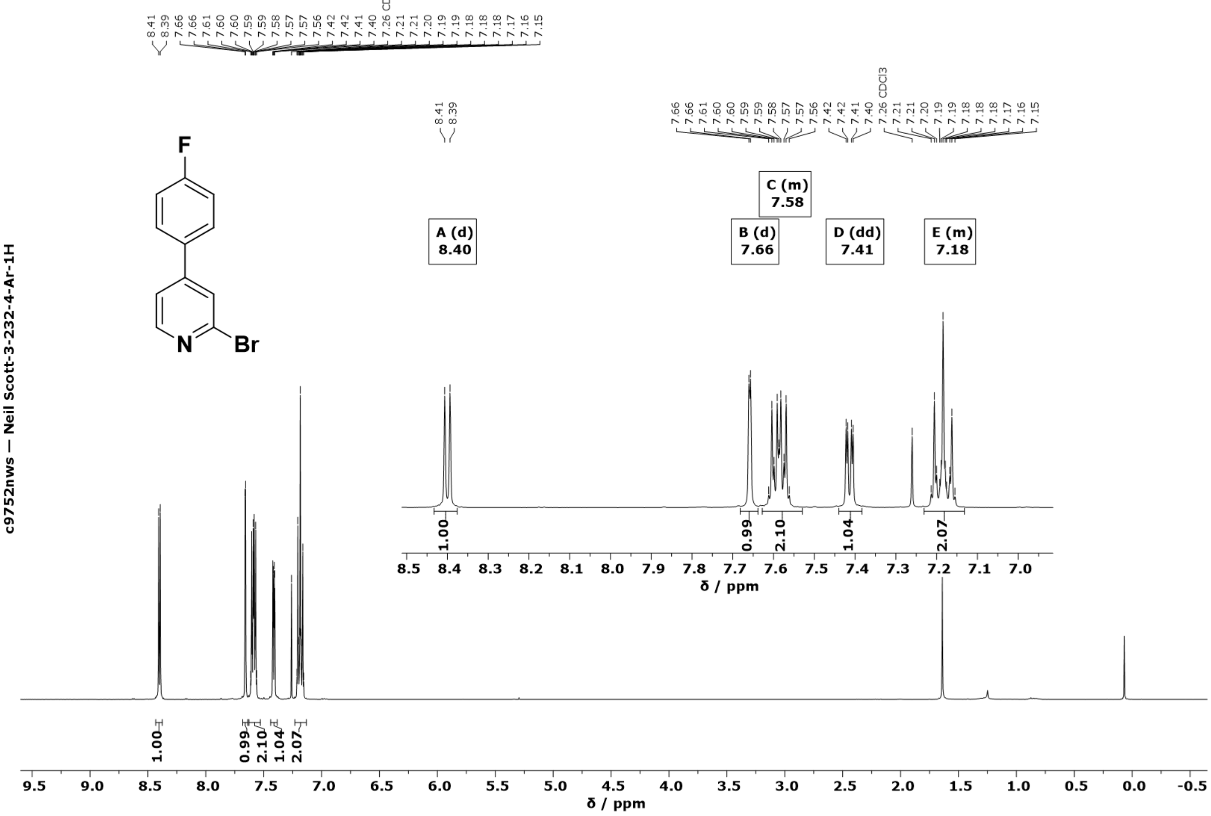

Figure S16 ^{1}H NMR ($CDCl_3$, 400 MHz, 32 scans) spectrum of $3a_{C4\text{-}Ar}$

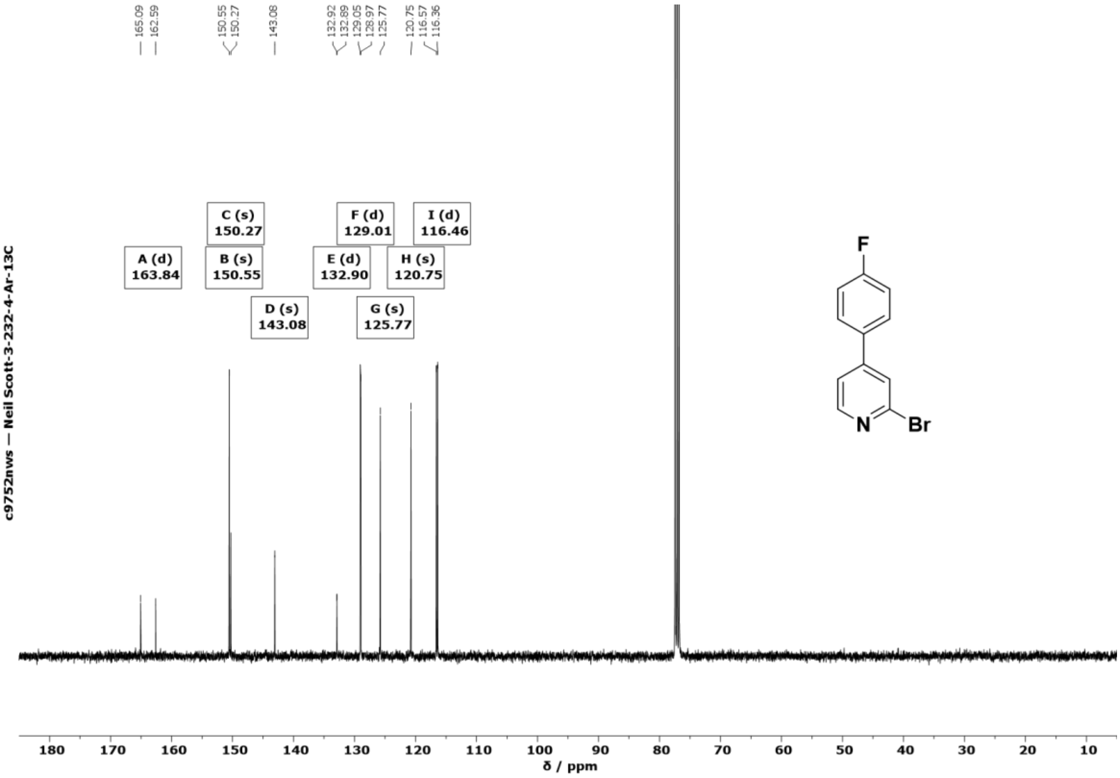

Figure S17 ^{13}C NMR ($CDCl_3$, 101 MHz, 2048 scans) spectrum of $3a_{C4\text{-}Ar}$

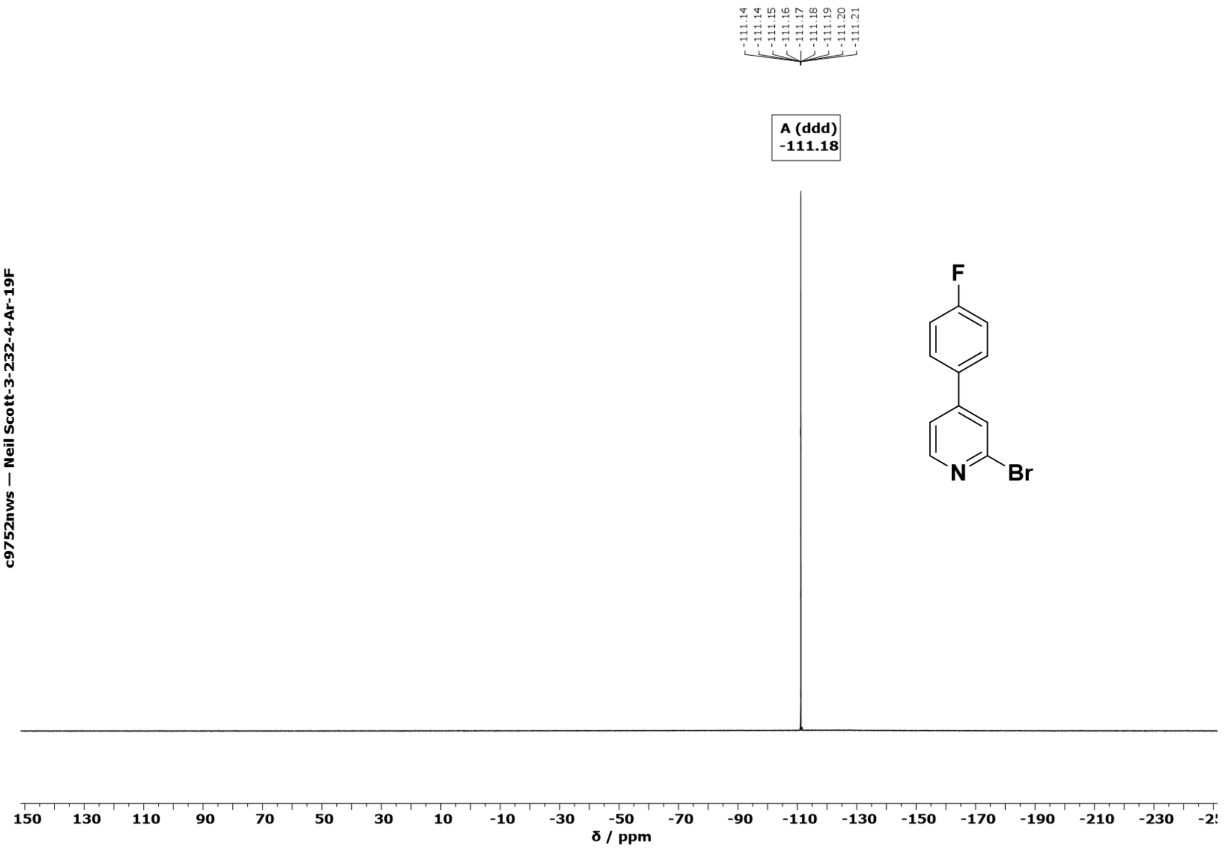

Figure S18 ^{19}F NMR ($CDCl_3$, 376 MHz, 128 scans) spectrum of 3a$_{C4\text{-}Ar}$

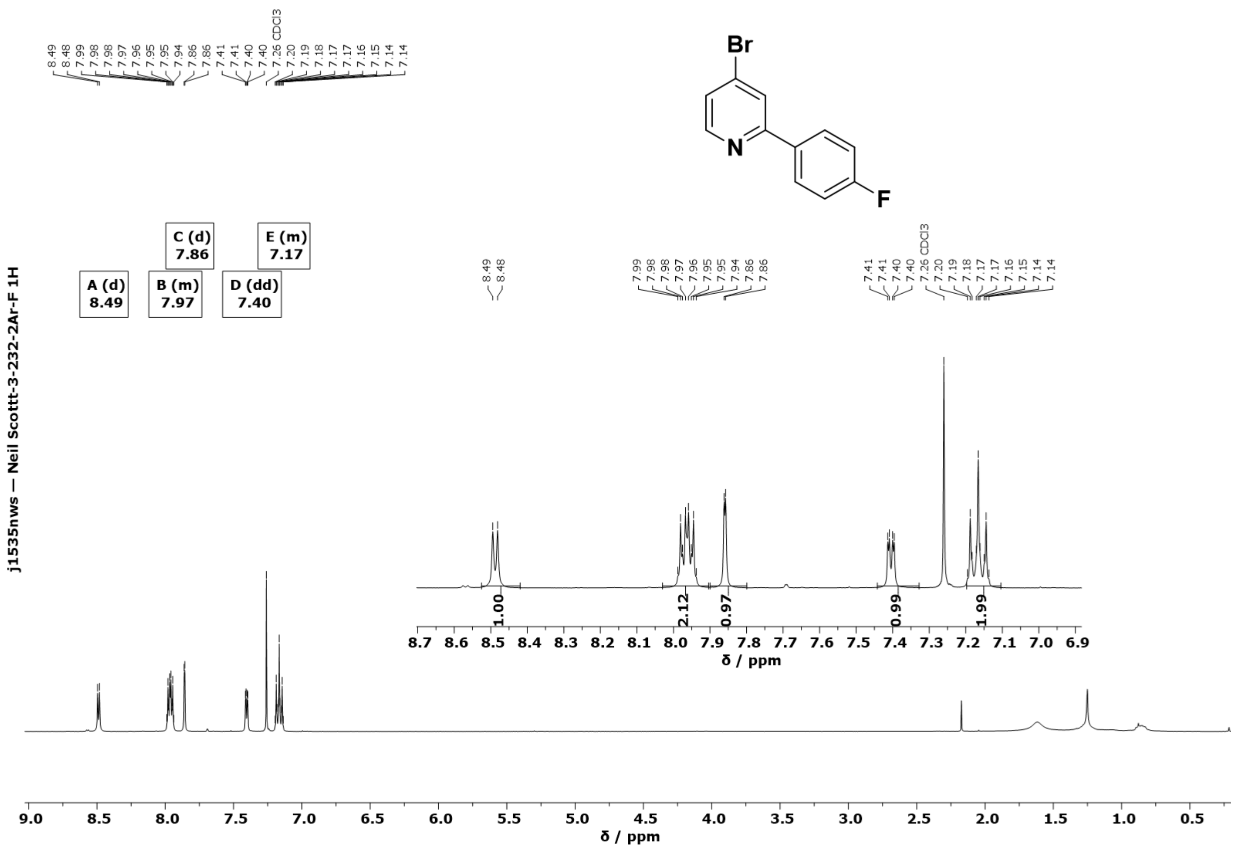

Figure S19 ^{1}H NMR ($CDCl_3$, 400 MHz, 32 scans) spectrum of 3a$_{C2\text{-}Ar}$

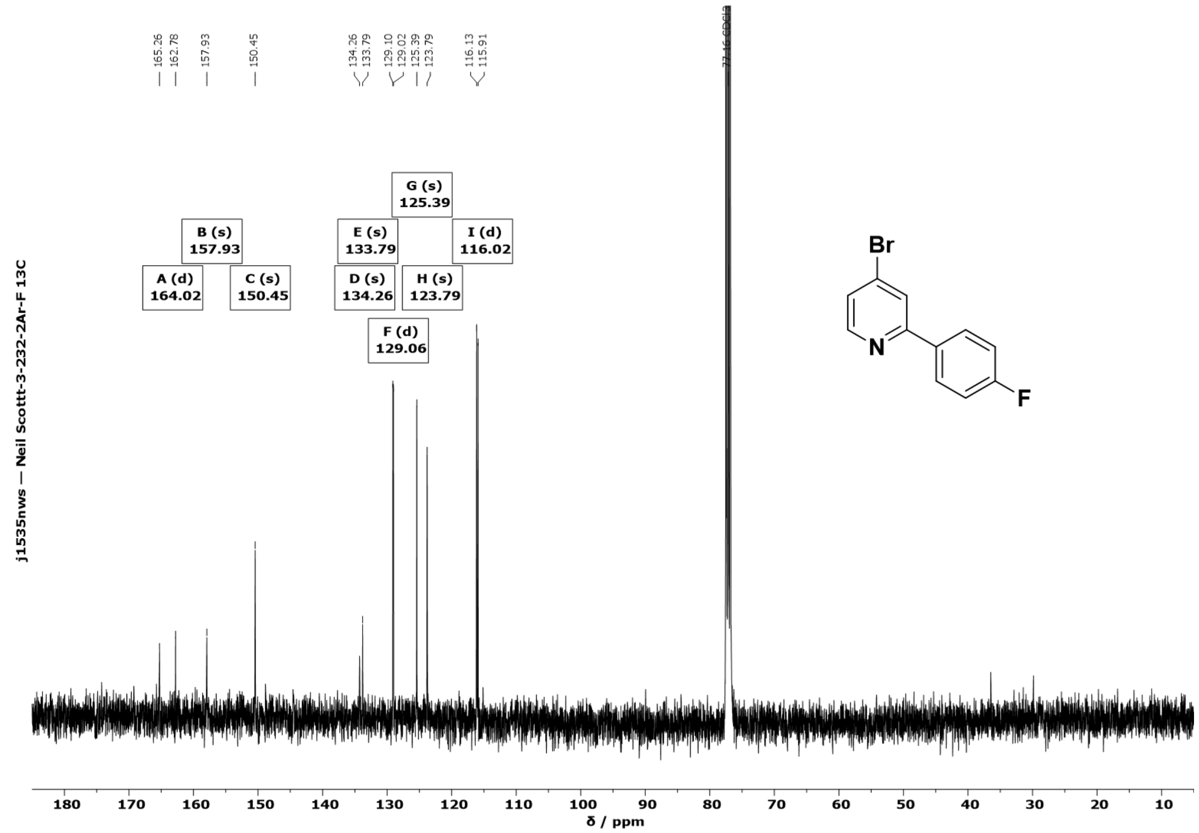

Figure S20 ^{13}C NMR ($CDCl_3$, 101 MHz, 2048 scans) spectrum of 3a*$_{C2-Ar}$*

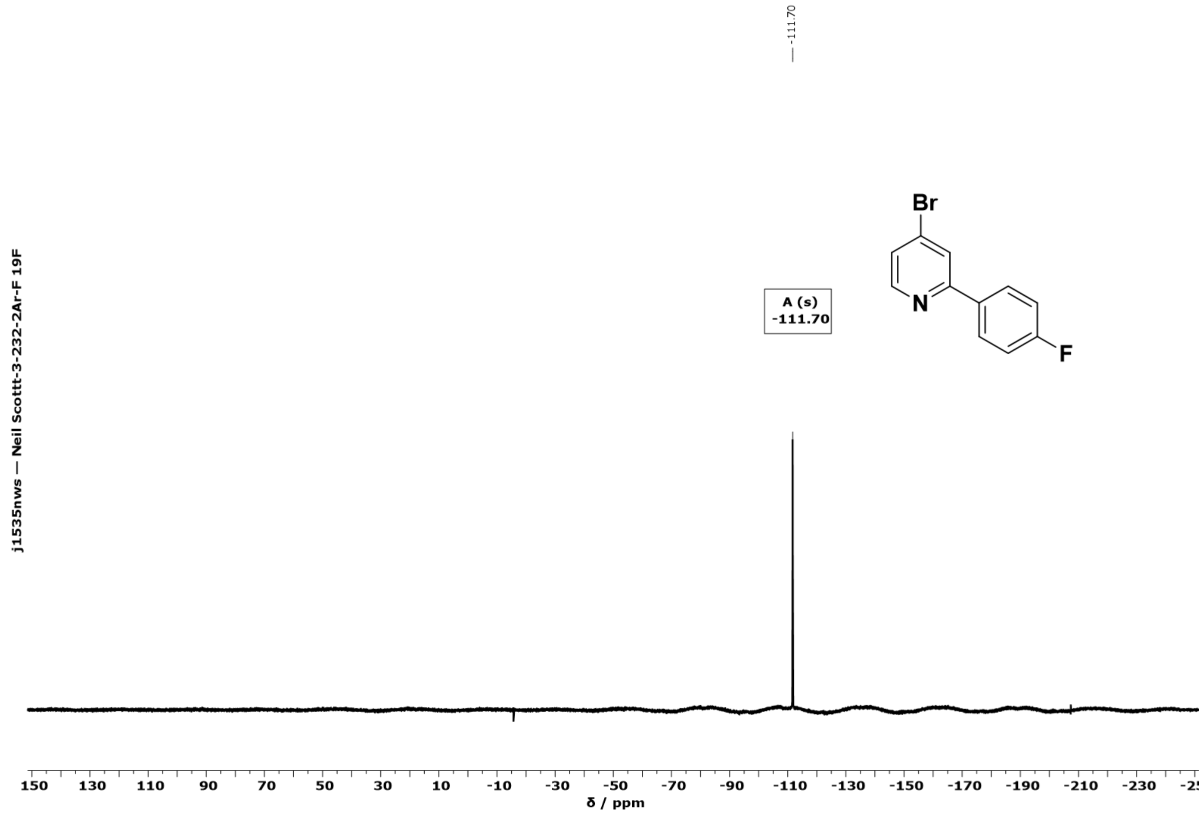

Figure S21 ^{19}F NMR ($CDCl_3$, 376 MHz, 128 scans) spectrum of 3a*$_{C2-Ar}$*

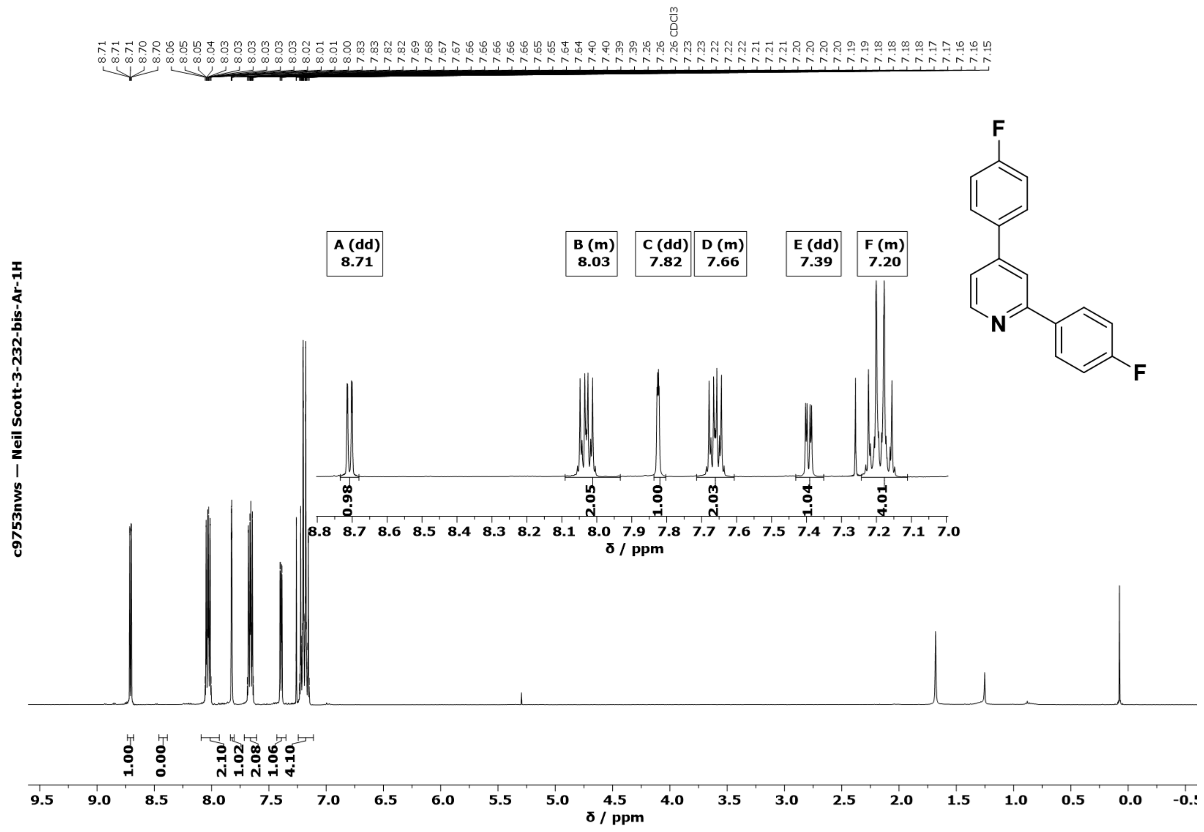

Figure 22 ^{1}H NMR ($CDCl_3$, 400 MHz, 32 scans) spectrum of 3a*diaryl*

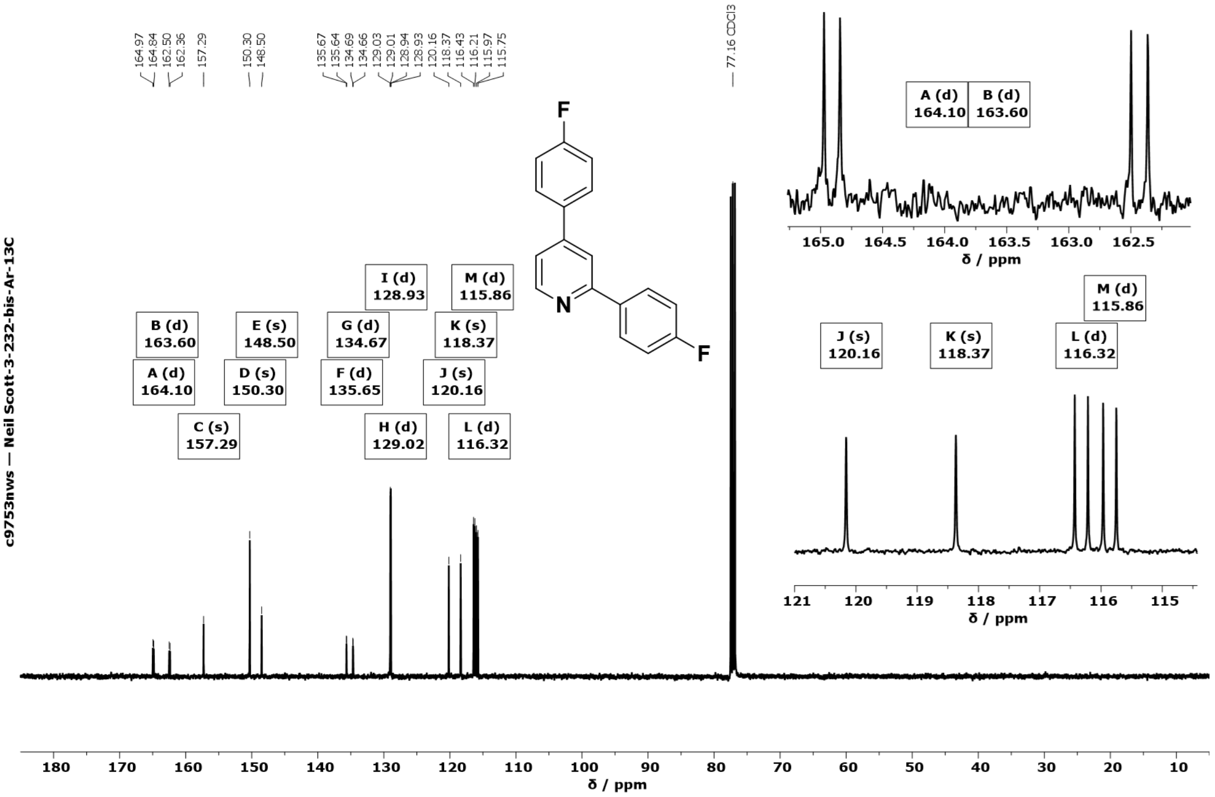

Figure S23 ^{13}C NMR ($CDCl_3$, 101 MHz, 2048 scans) spectrum of 3a*diaryl*.

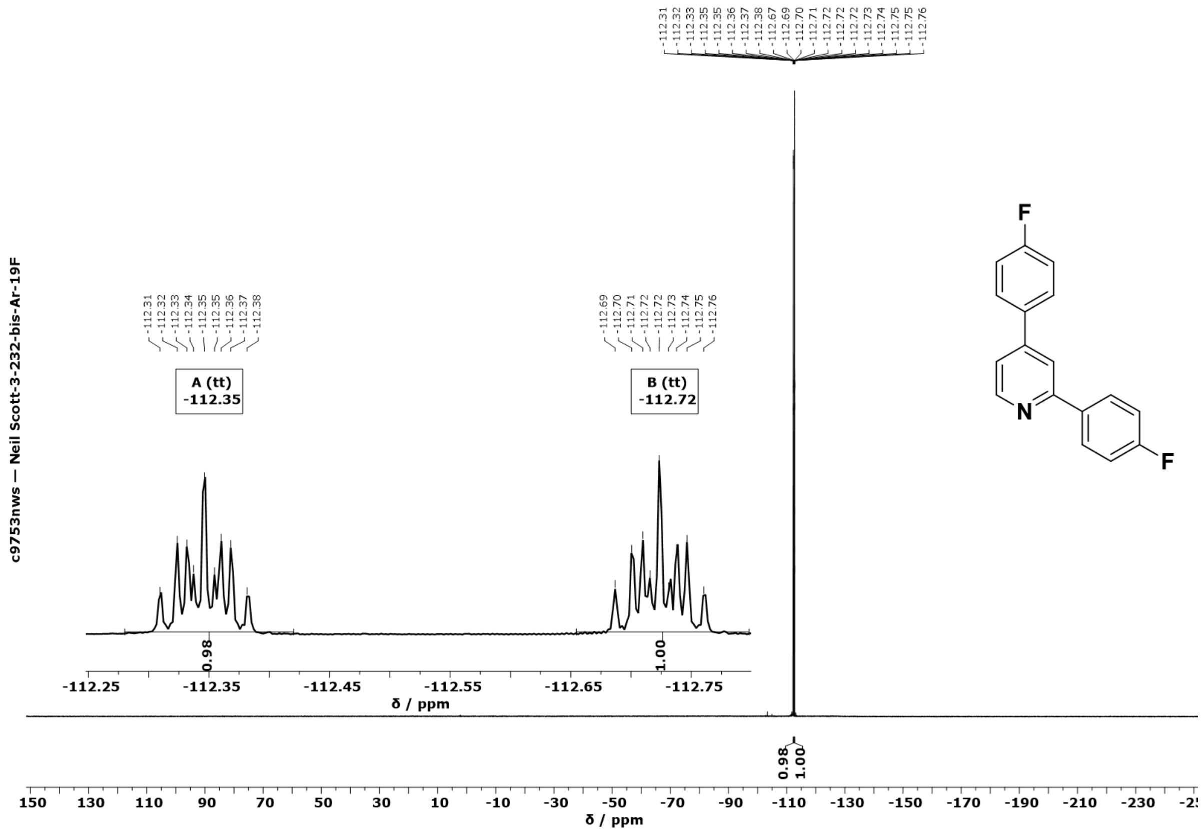

Figure S24 ^{19}F NMR ($CDCl_3$, 376 MHz, 128 scans) spectrum of $3a_{diaryl}$

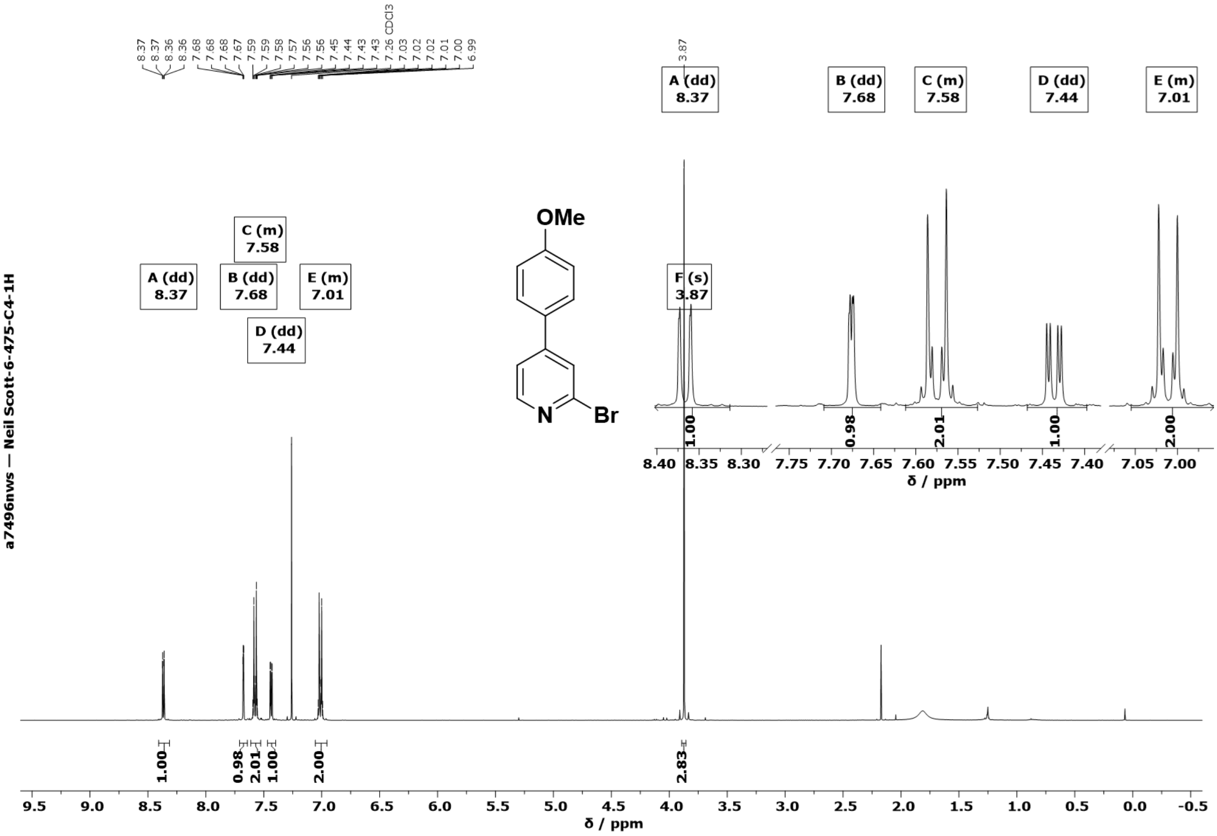

Figure S25 ^{1}H NMR ($CDCl_3$, 400 MHz, 32 scans) spectrum of $3b_{C4-Ar}$.

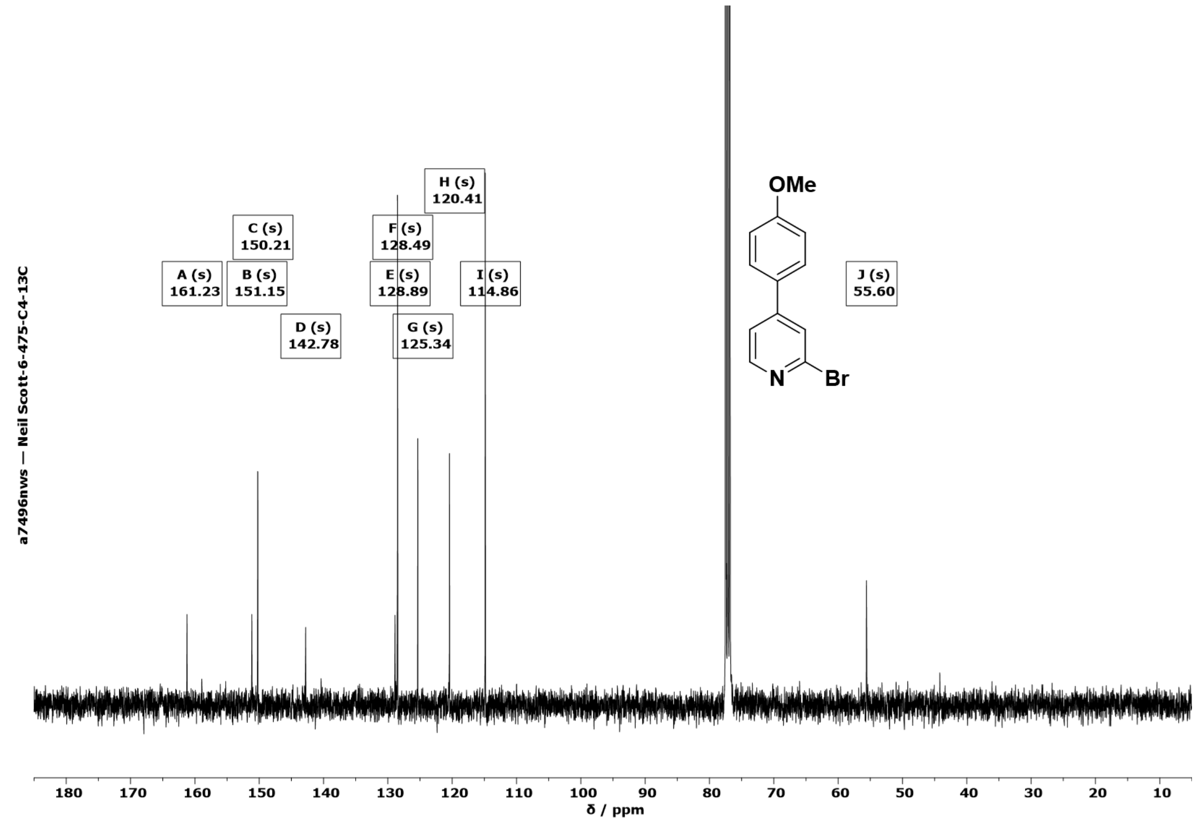

Figure S26 ^{13}C NMR ($CDCl_3$, 101 MHz, 1024 scans) spectrum of 3bC$_{4\text{-}Ar}$

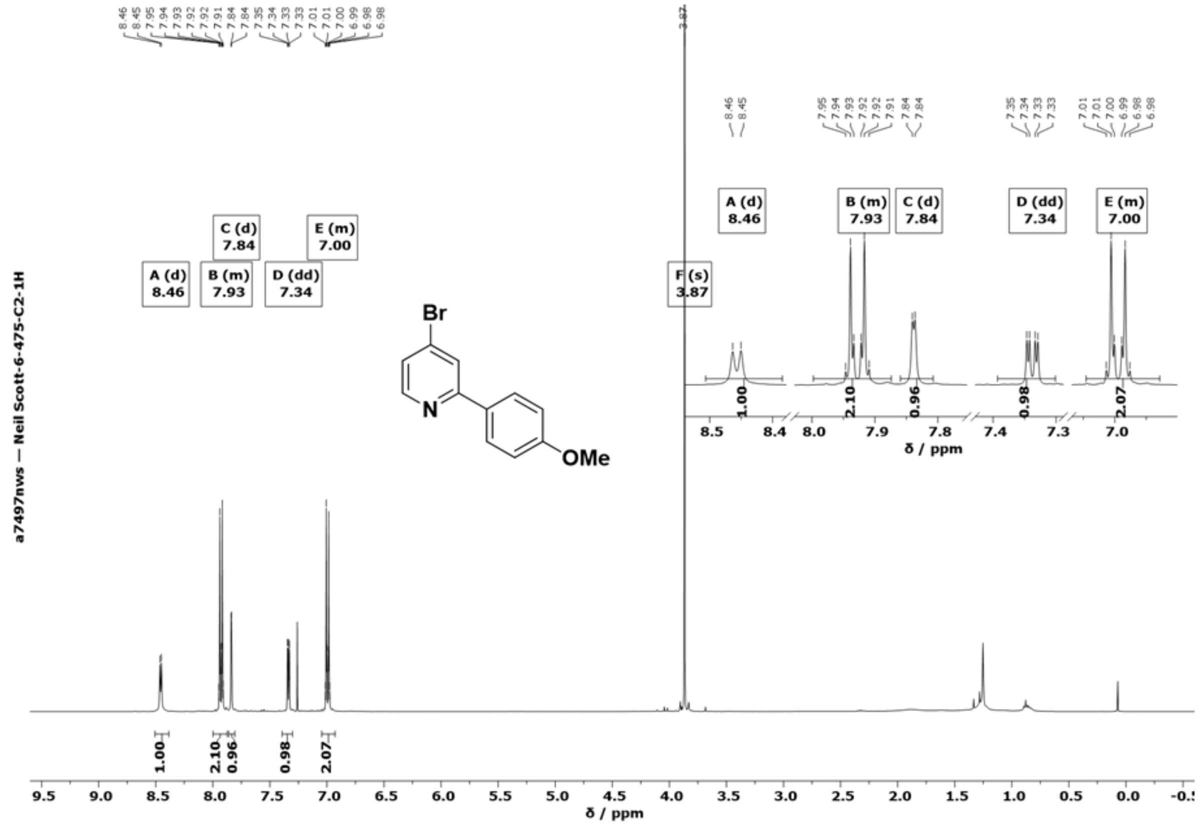

Figure S27 ^{1}H NMR ($CDCl_3$, 400 MHz, 32 scans) spectrum of 3b$_{C2\text{-}Ar}$.

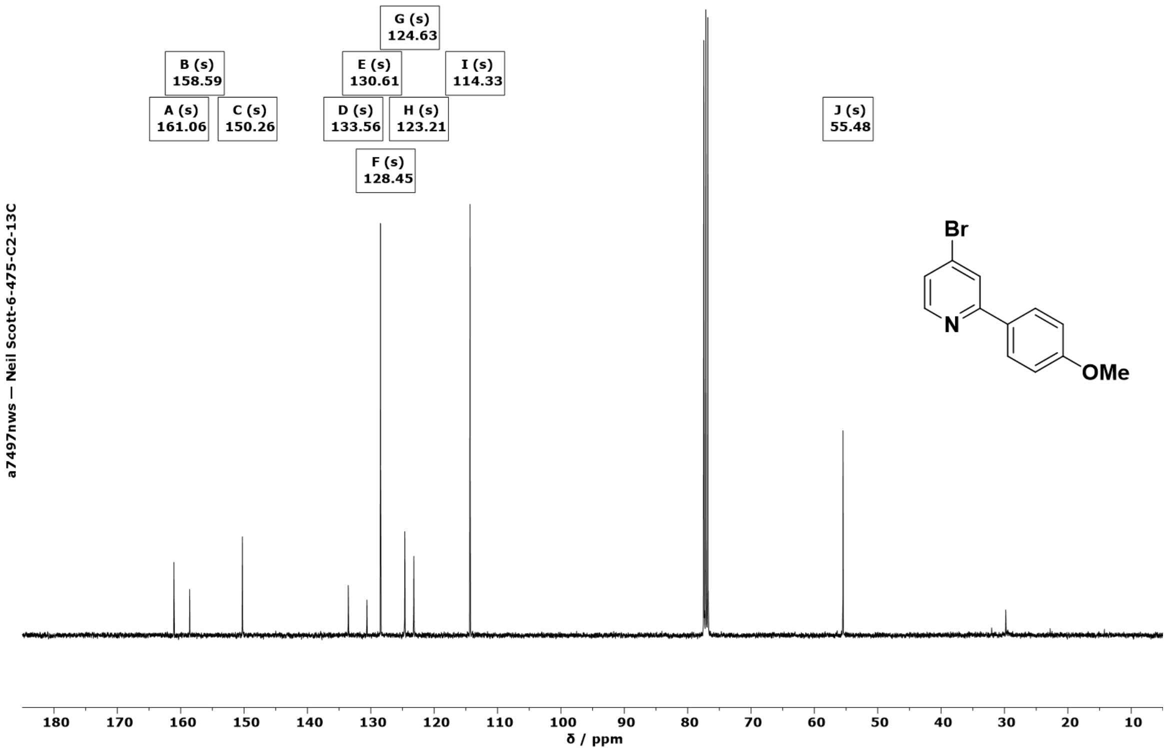

Figure S28 ^{13}C NMR ($CDCl_3$, 101 MHz, 1024 scans) spectrum of 3b$_{C2\text{-}Ar}$.

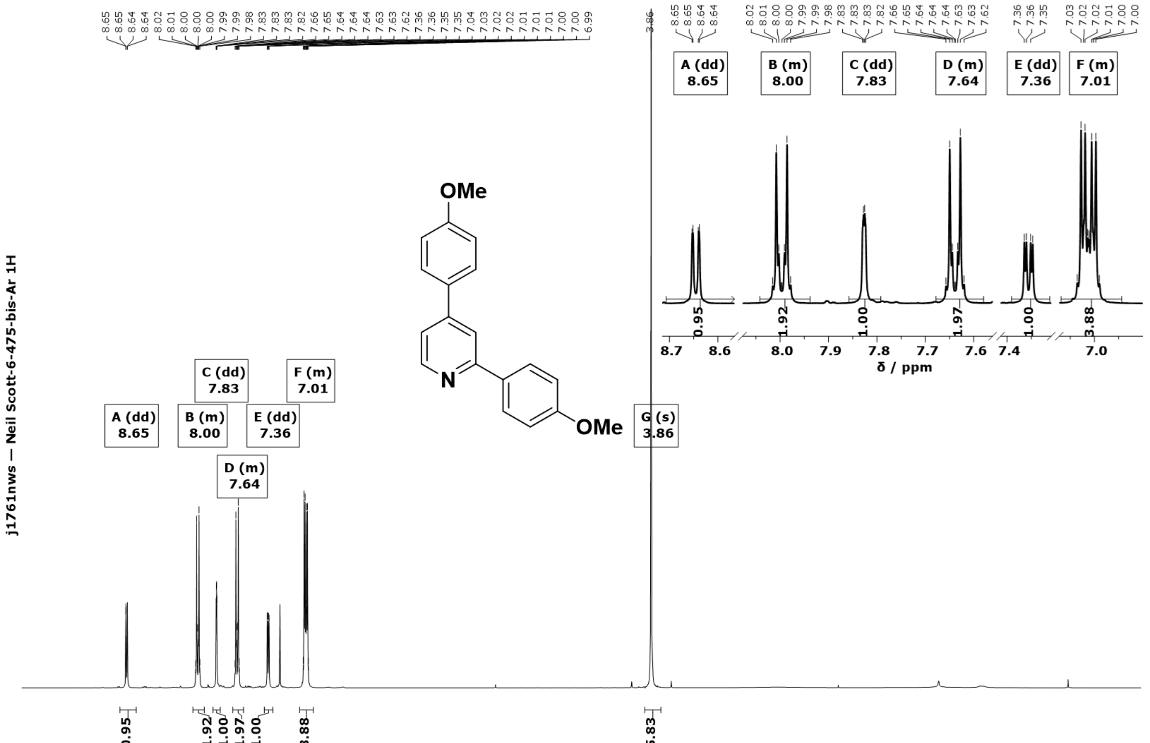

Figure S29 ^{1}H NMR ($CDCl_3$, 400 MHz, 32 scans) spectrum of 3b$_{diaryl}$

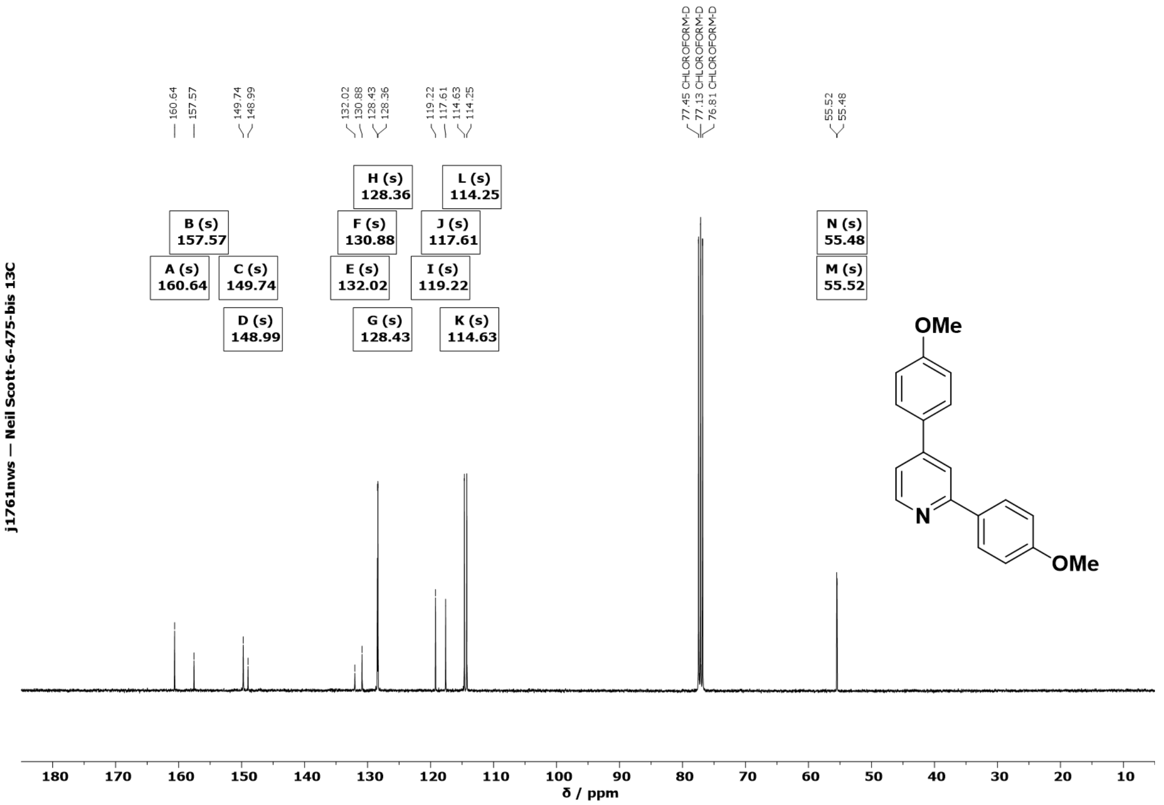

Figure S30 ^{13}C NMR ($CDCl_3$, 101 MHz, 1024 scans) spectrum of 3b$_{diaryl}$.

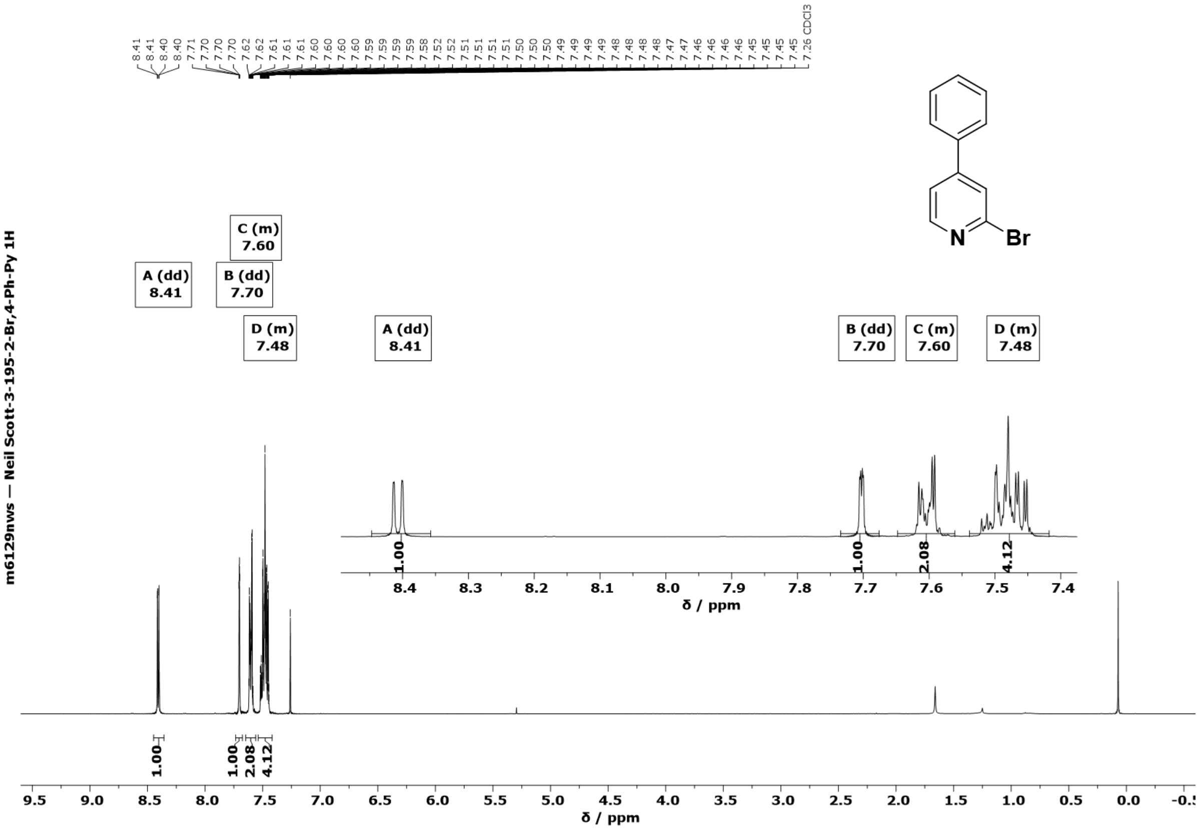

Figure S31 ^{1}H NMR ($CDCl_3$, 400 MHz, 32 scans) spectrum of 3c$_{C4\text{-}Ar}$.

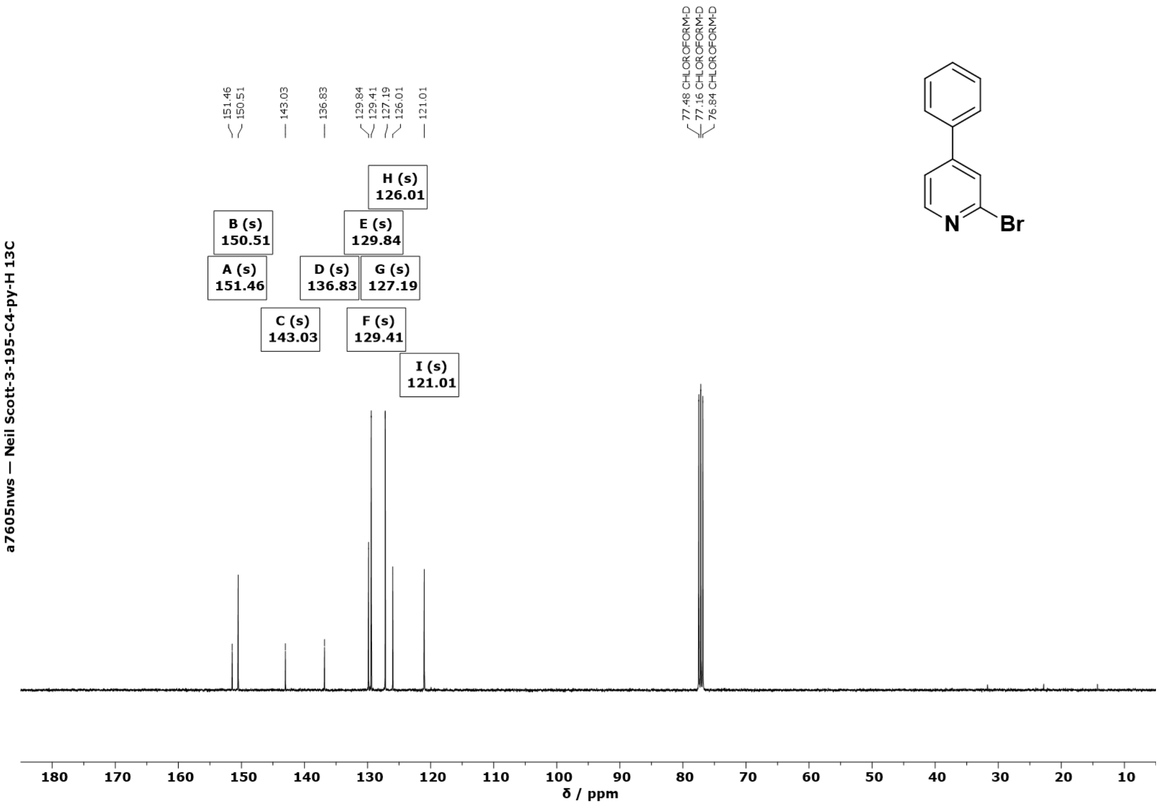

Figure S32 ^{13}C NMR ($CDCl_3$, 101 MHz, 1024 scans) spectrum of $3c_{C4-Ar}$.

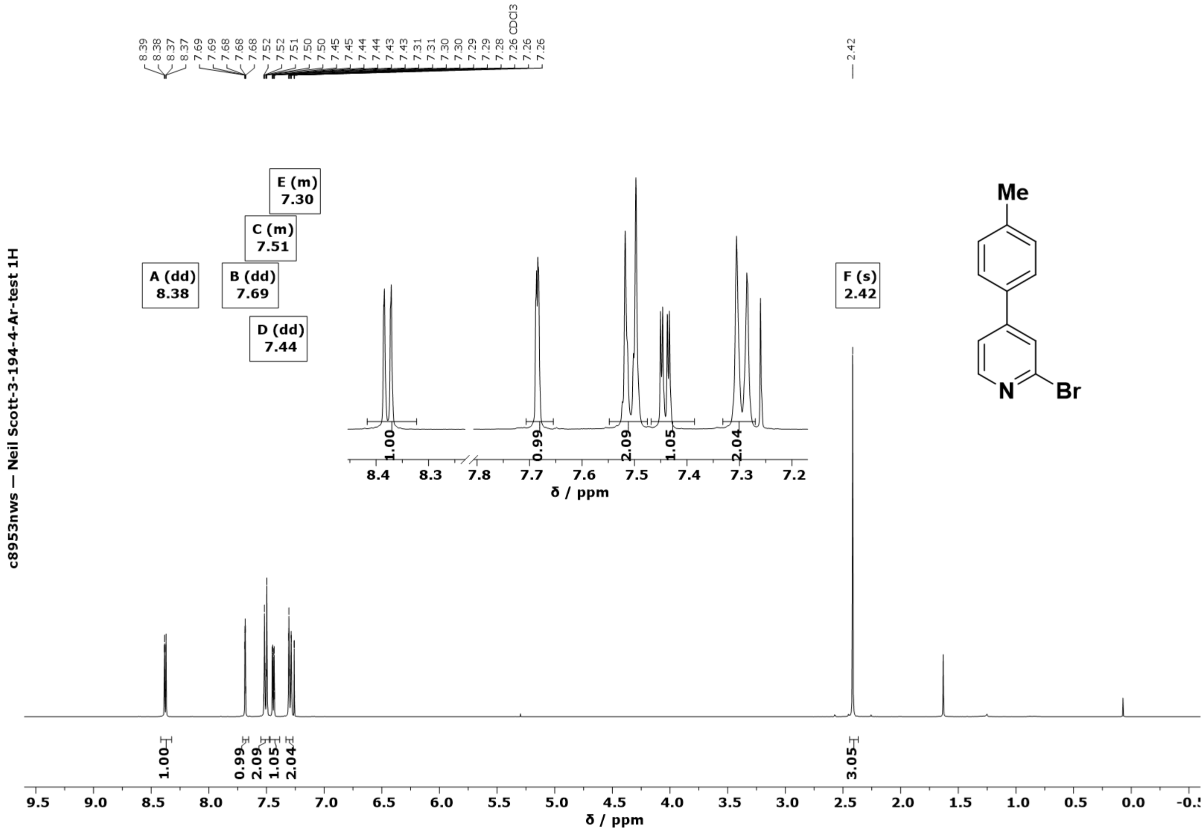

Figure S33 ^{1}H NMR ($CDCl_3$, 400 MHz, 32 scans) spectrum of $3d_{C4-Ar}$.

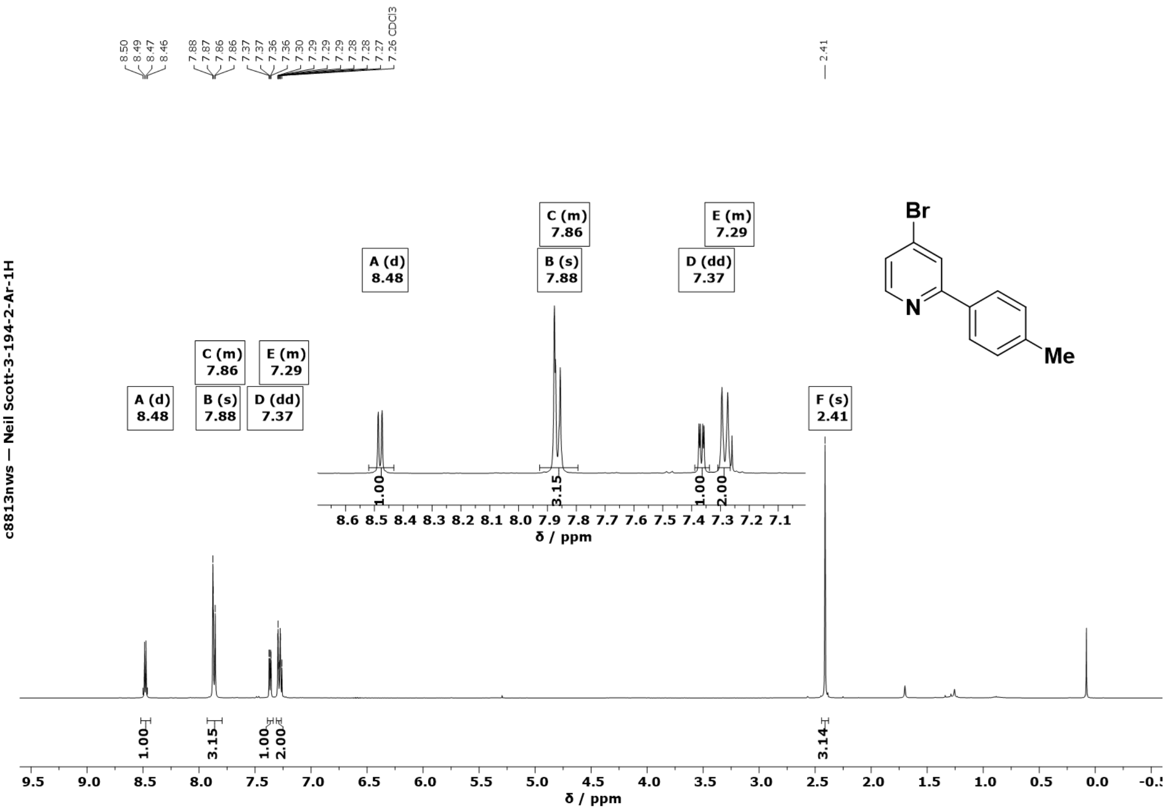

Figure S34 ^{1}H NMR ($CDCl_3$, 400 MHz, 32 scans) spectrum of 3d$_{C2\text{-}Ar}$.

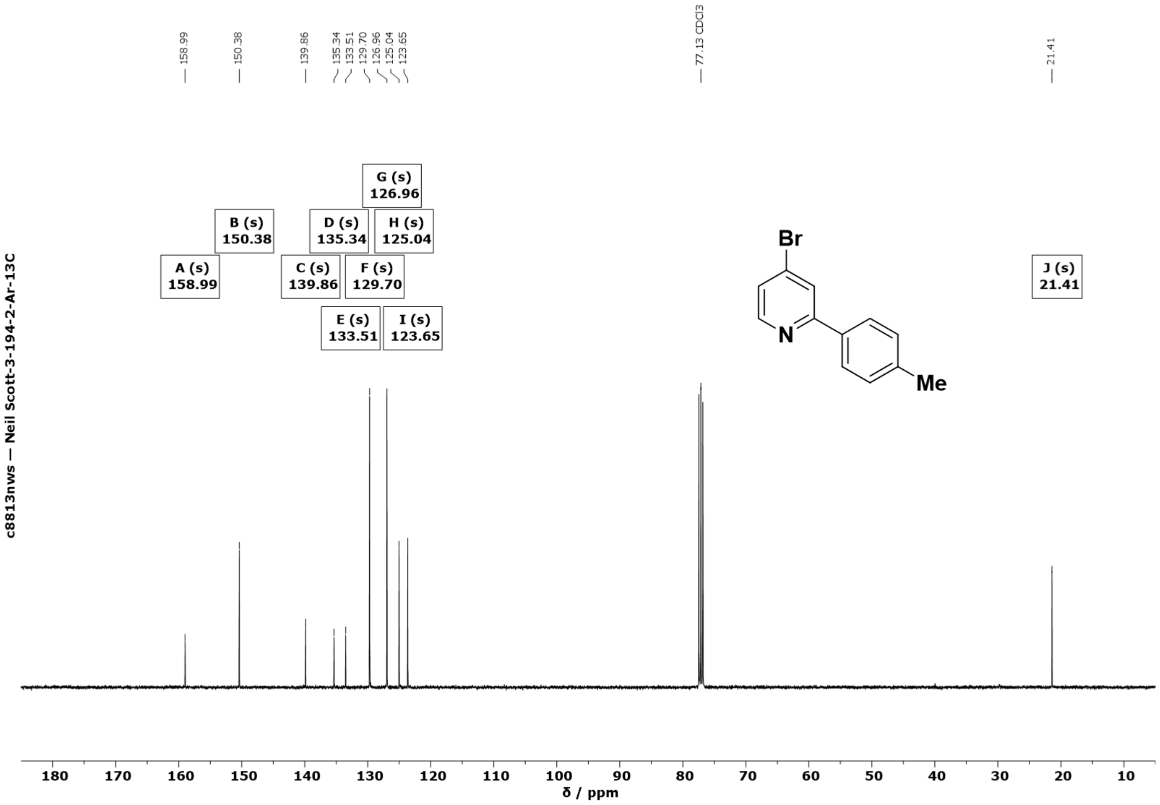

Figure S35 ^{13}C NMR ($CDCl_3$, 101 MHz, 1024 scans) spectrum of 3d$_{C2\text{-}Ar}$.

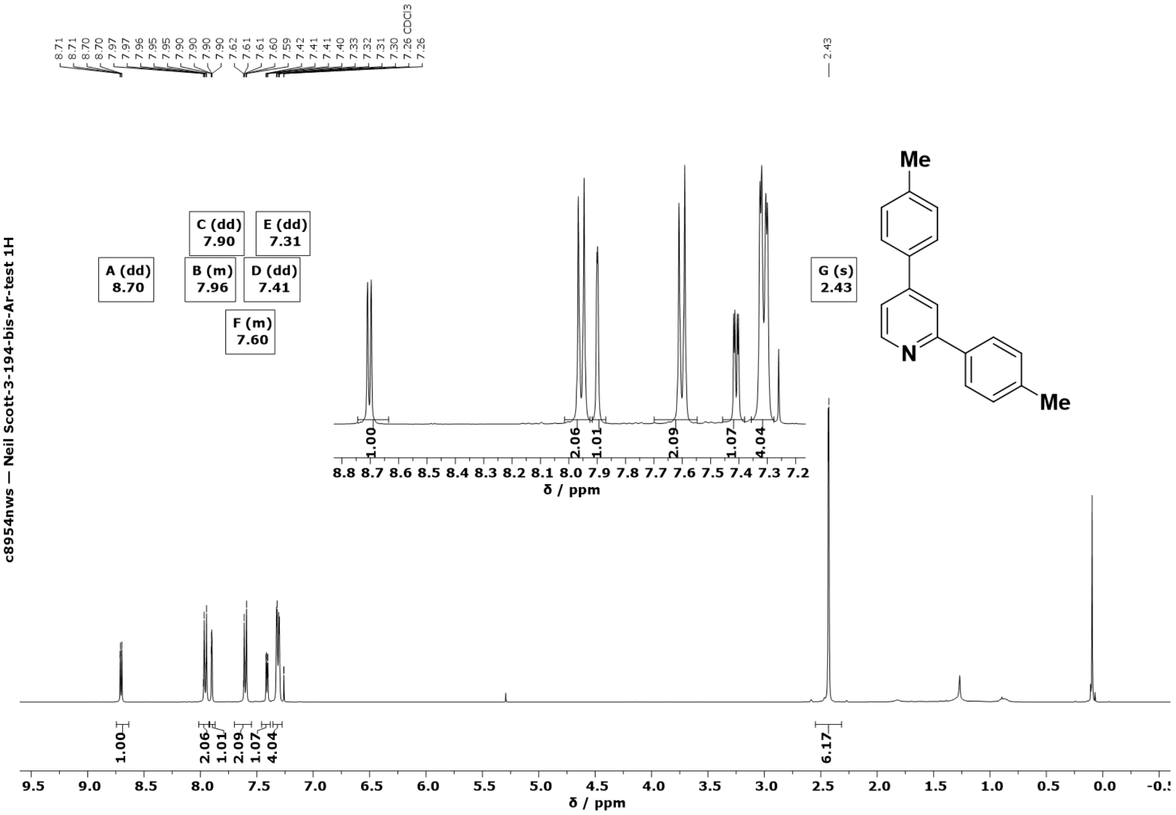

Figure S36 ^{1}H NMR ($CDCl_3$, 400 MHz, 32 scans) spectrum of 3d$_{diaryl}$.

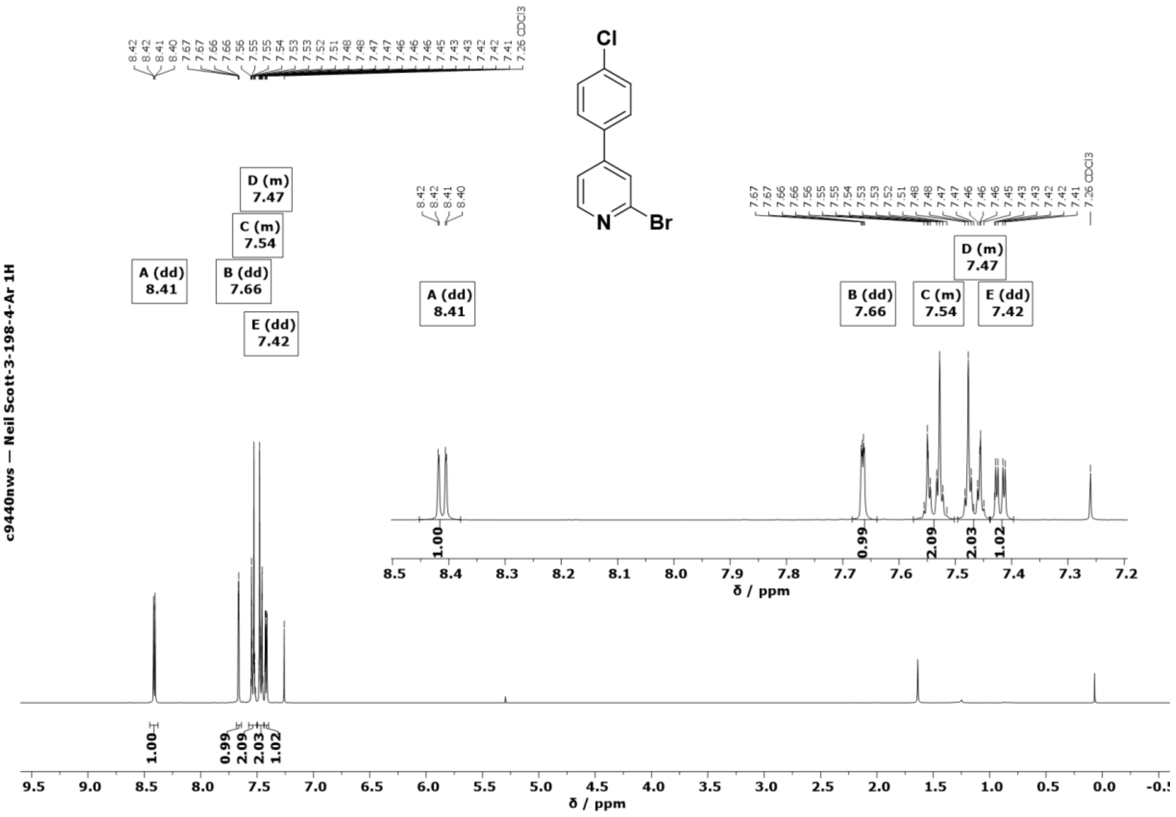

Figure S37 ^{1}H NMR ($CDCl_3$, 400 MHz, 32 scans) spectrum of 3e$_{C4\text{-}Ar}$.

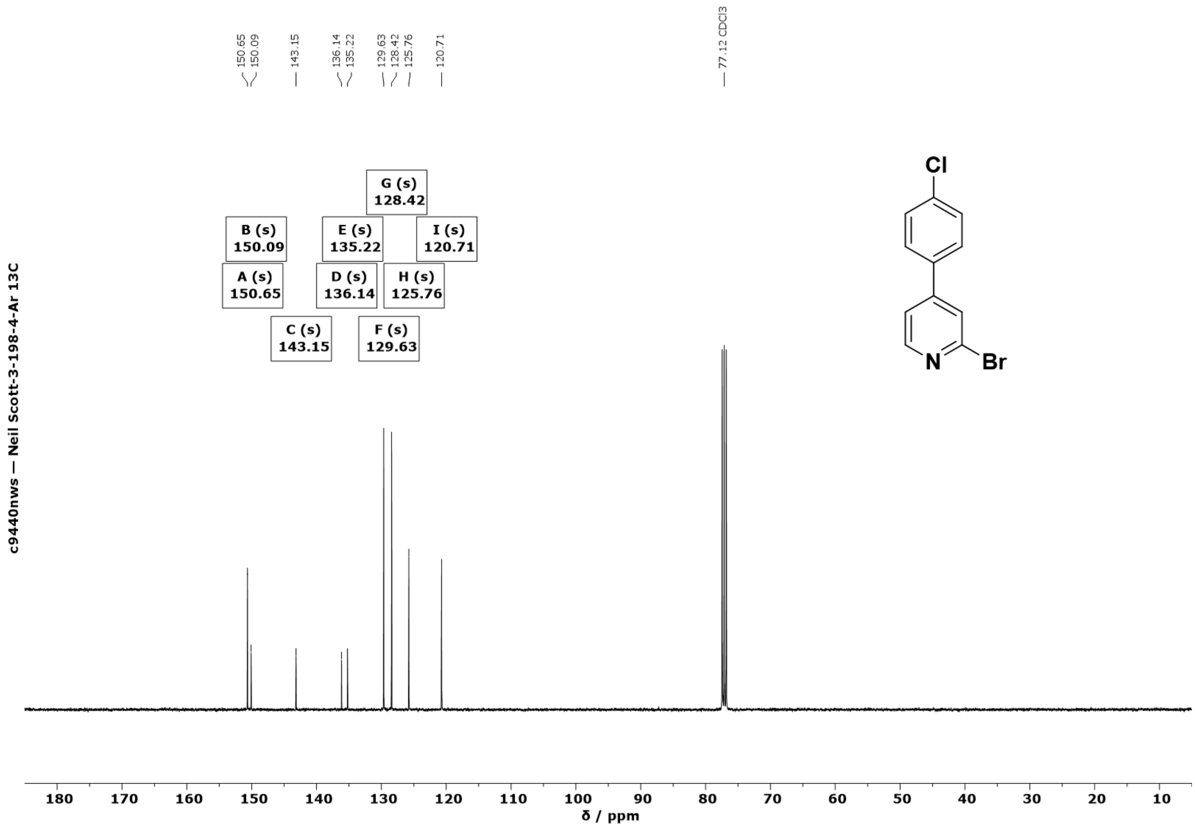

Figure S38 ^{13}C NMR ($CDCl_3$, 101 MHz, 1024 scans) spectrum of 3e$_{C4\text{-}Ar}$.

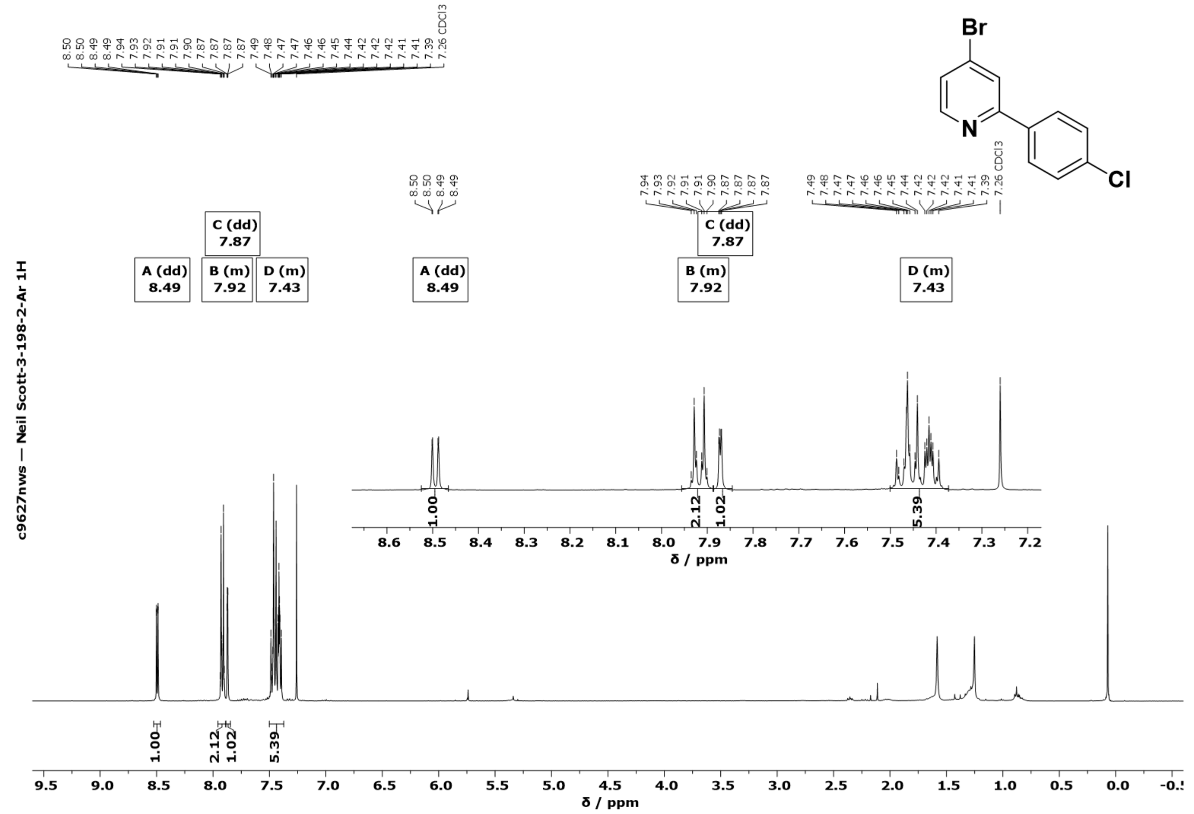

Figure S39 ^{1}H NMR ($CDCl_3$, 400 MHz, 32 scans) spectrum of 3e$_{C2\text{-}Ar}$.

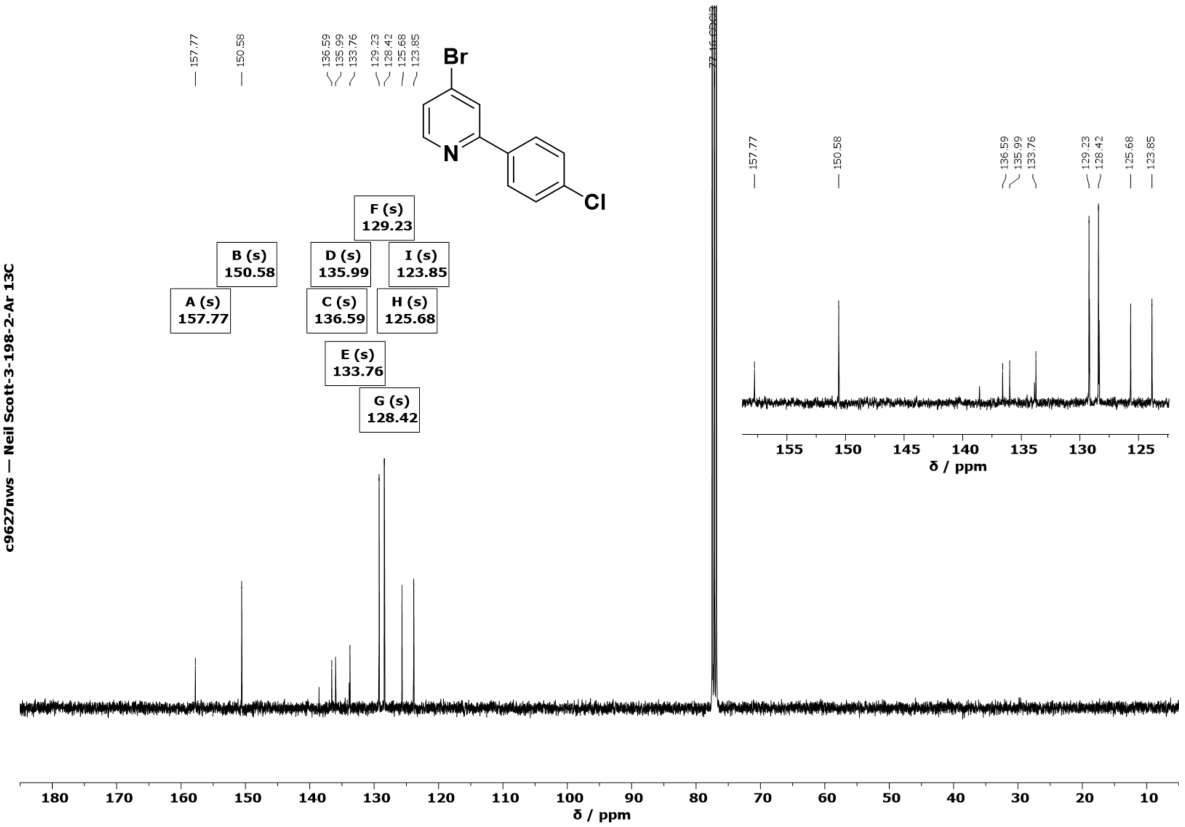

Figure S40 ^{13}C NMR ($CDCl_3$, 101 MHz, 1024 scans) spectrum of 3e$_{C2\text{-}Ar}$.

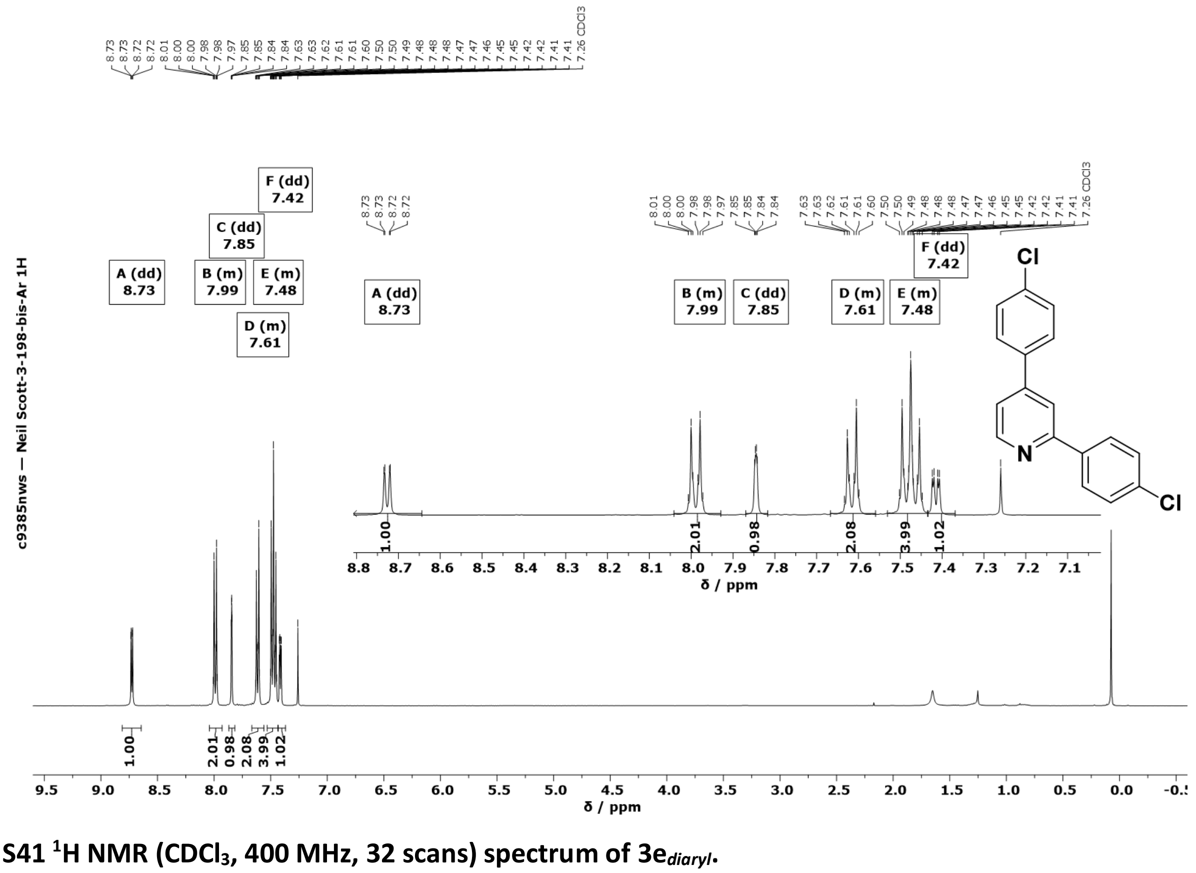

Figure S41 ^{1}H NMR ($CDCl_3$, 400 MHz, 32 scans) spectrum of 3e$_{diaryl}$.

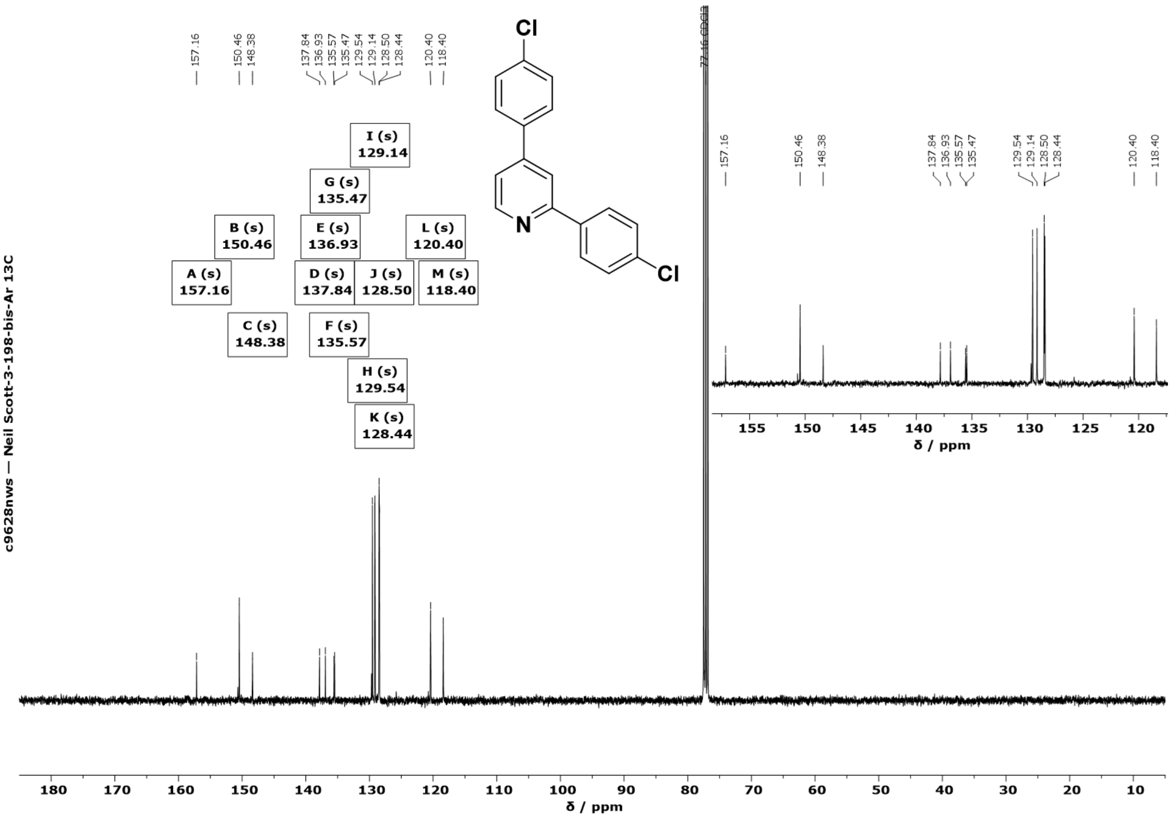

Figure S42 ^{13}C NMR ($CDCl_3$, 101 MHz, 1024 scans) spectrum of 3e$_{diaryl}$.

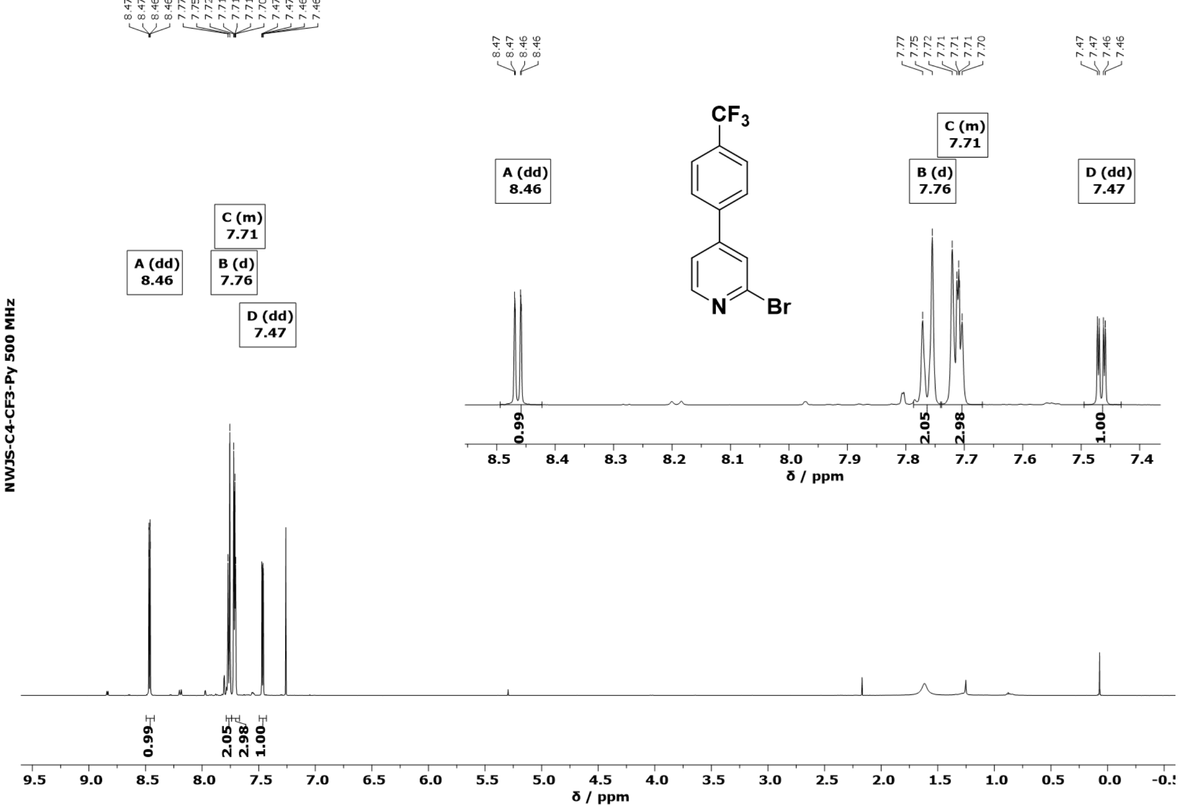

Figure S43 ^{1}H NMR ($CDCl_3$, 500 MHz, 64 scans) spectrum of 3f$_{C4\text{-}Ar}$.

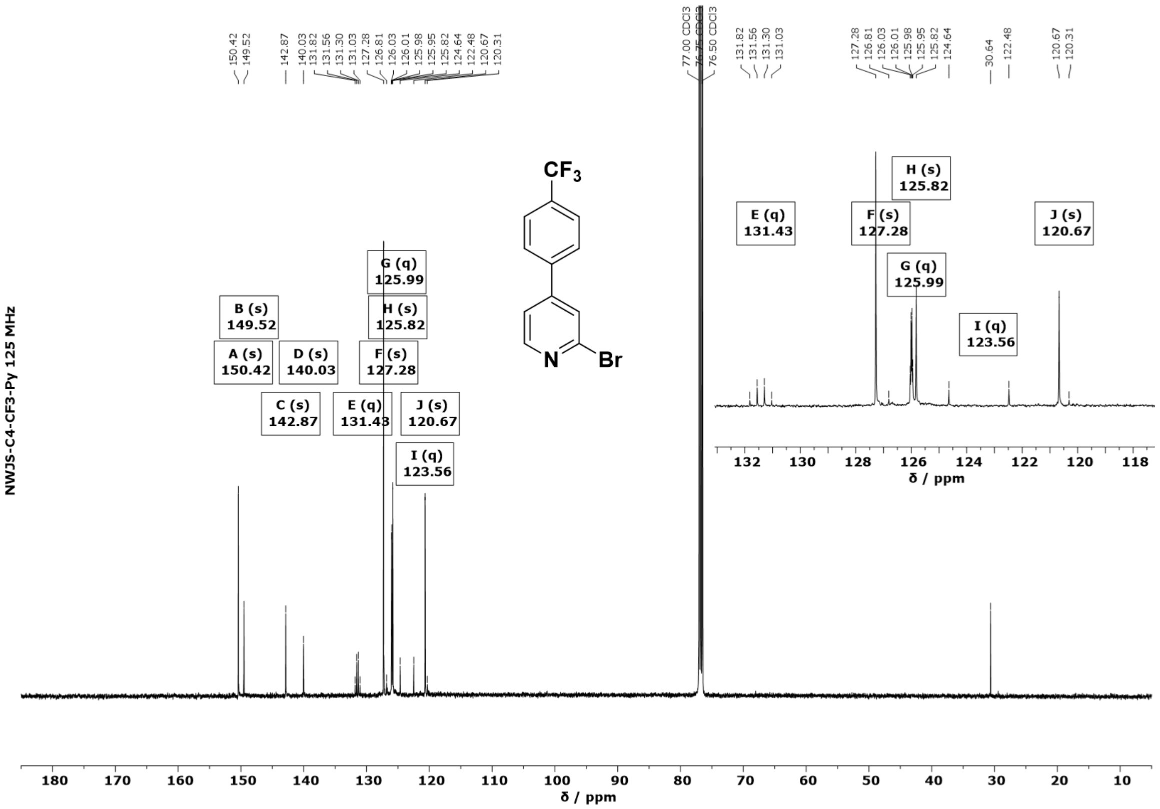

Figure S44 ^{13}C NMR ($CDCl_3$, 125 MHz, 1024 scans) spectrum of $3f_{C4\text{-}Ar}$.

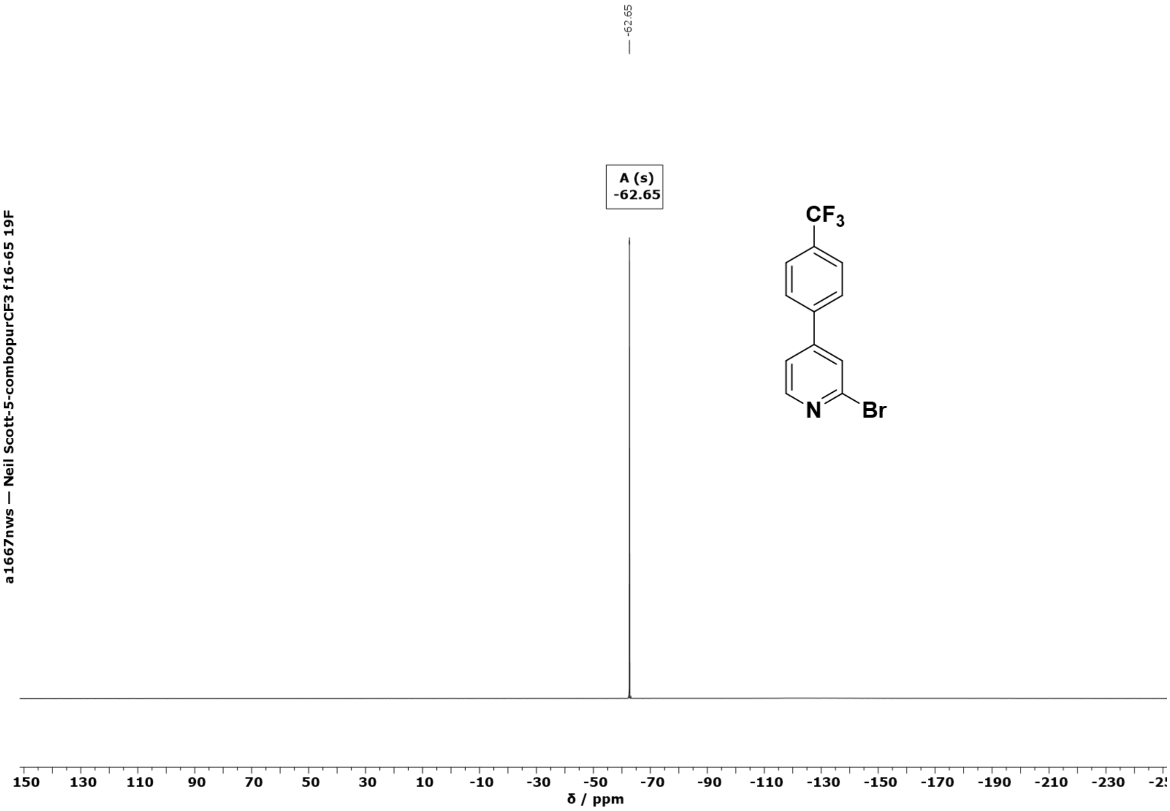

Figure S45 ^{19}F NMR ($CDCl_3$, 376 MHz, 128 scans) spectrum of $3f_{C4\text{-}Ar}$.

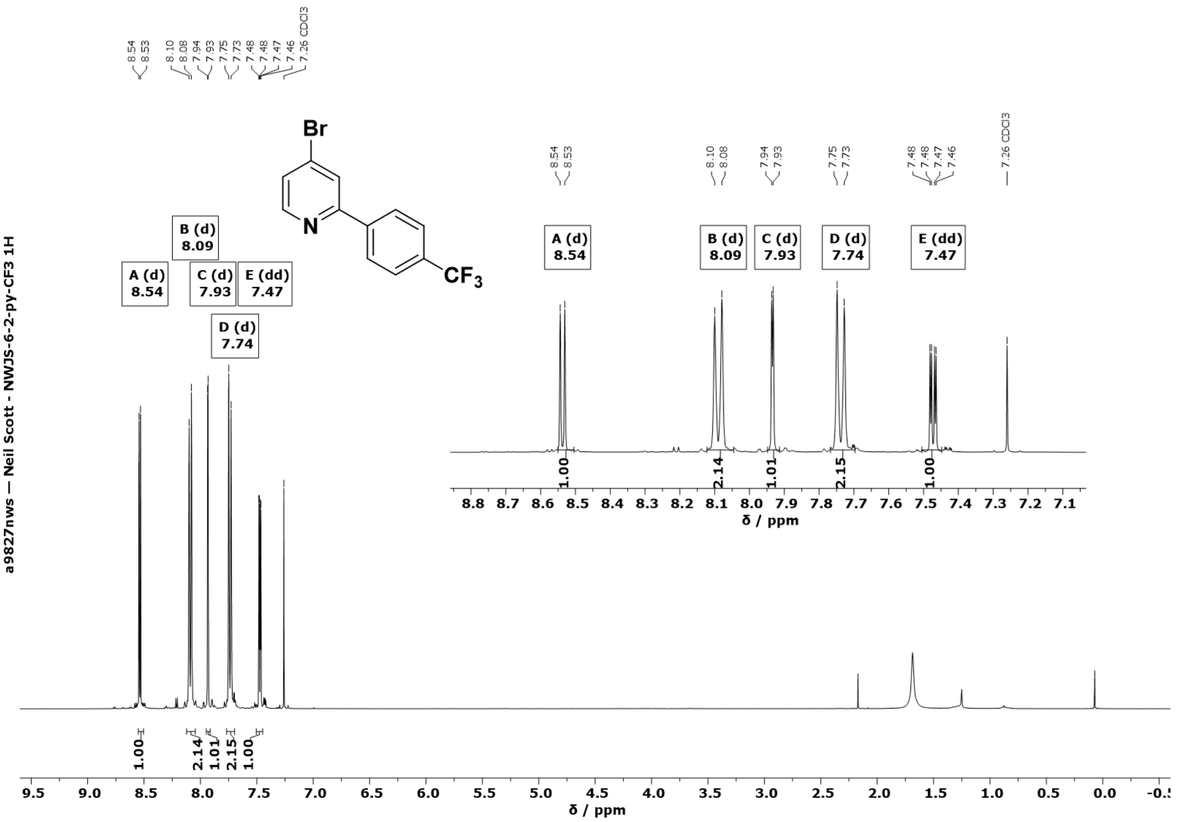

Figure S46 ^{1}H NMR ($CDCl_3$, 400 MHz, 32 scans) spectrum of 3f$_{C2\text{-}Ar}$.

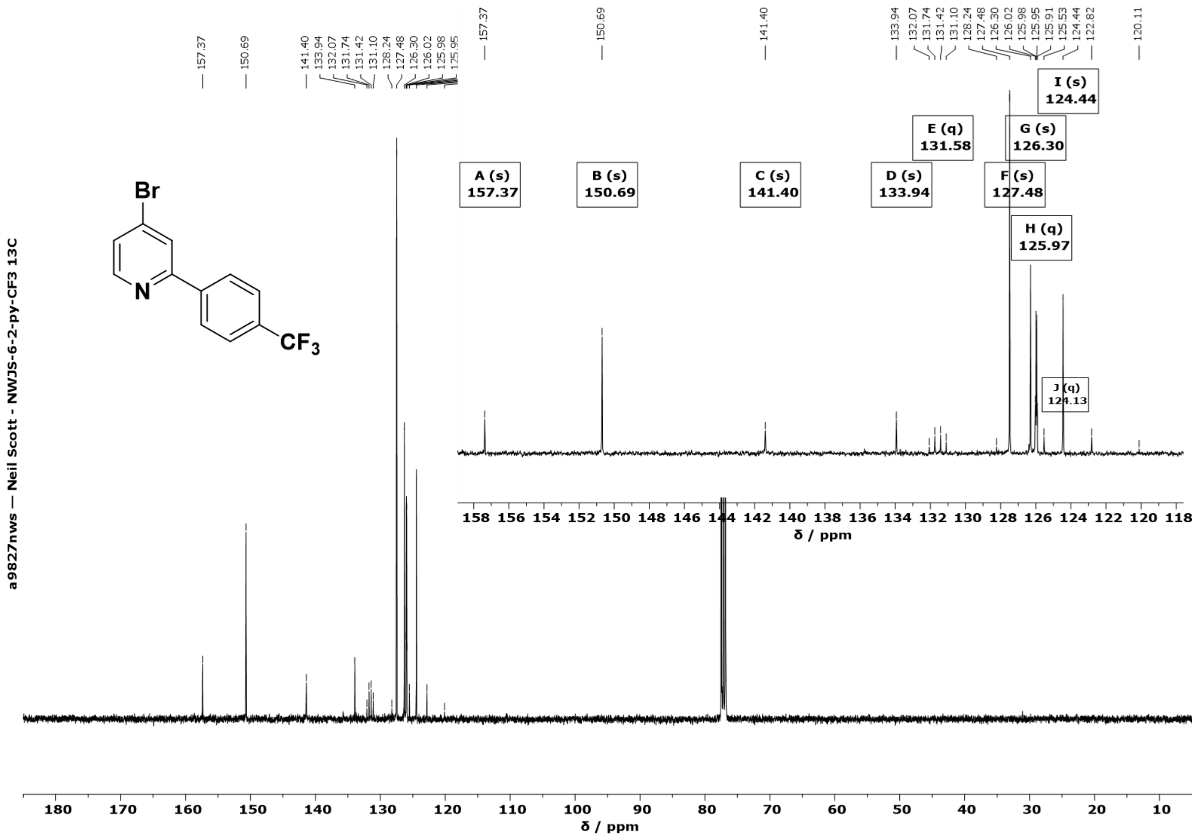

Figure S47 ^{13}C NMR ($CDCl_3$, 101 MHz, 2048 scans) spectrum of 3f$_{C2\text{-}Ar}$

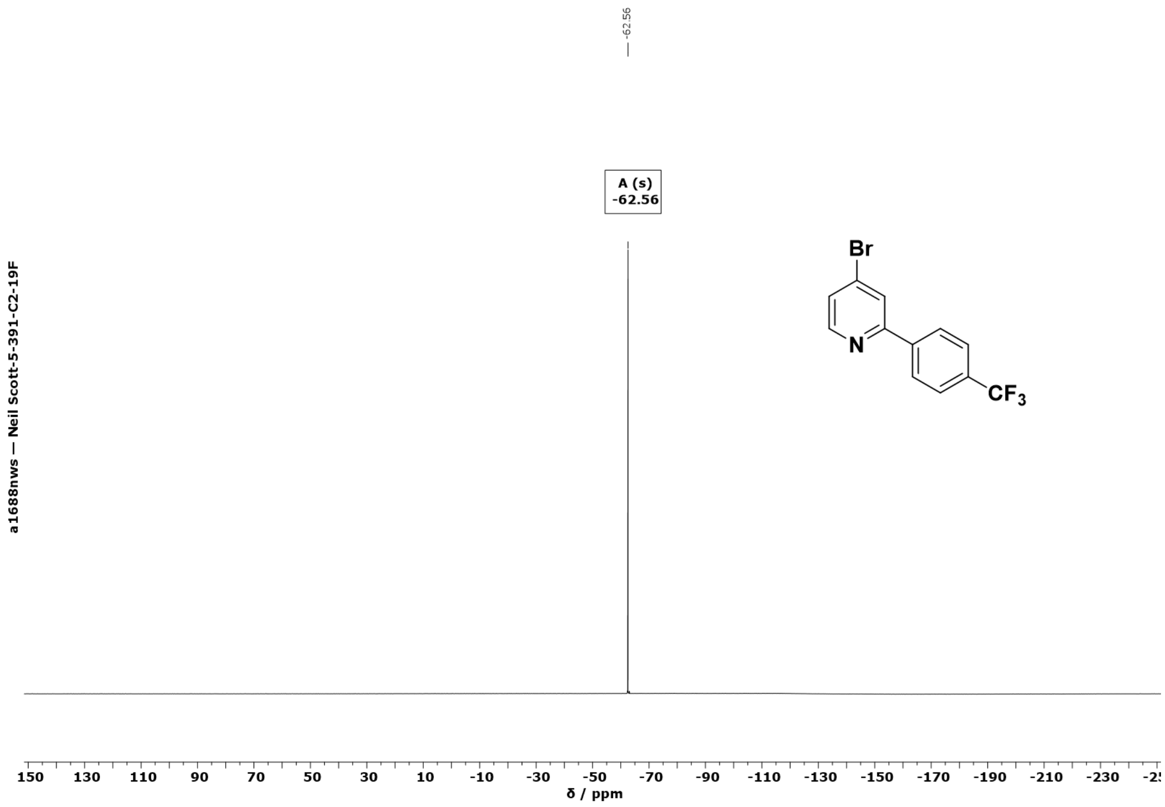

Figure S48 ^{19}F NMR ($CDCl_3$, 376 MHz, 128 scans) spectrum of 3f$_{C2\text{-}Ar}$.

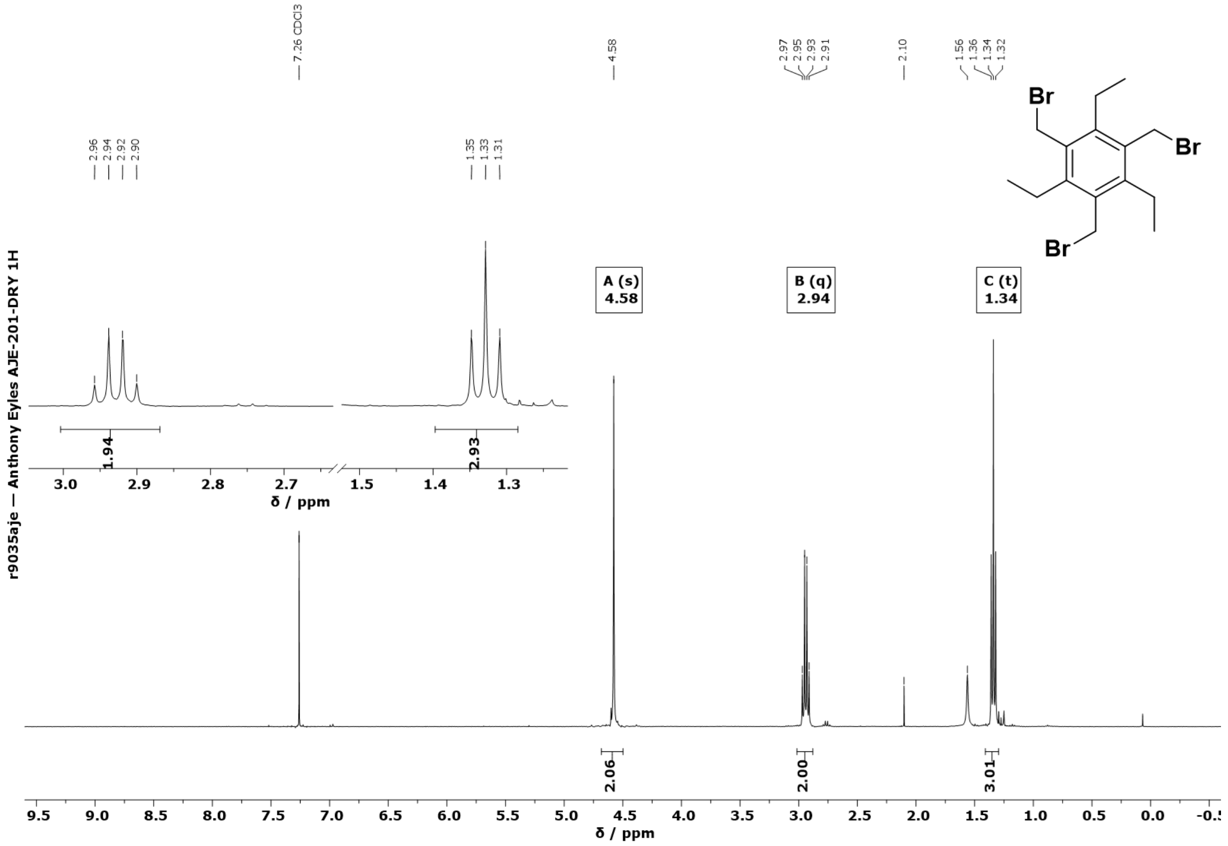

Figure S49 1H NMR ($CDCl_3$, 400 MHz, 32 scans) spectrum for 1,3,5-tris(bromomethyl)-2,4,6-triethyl-benzene.

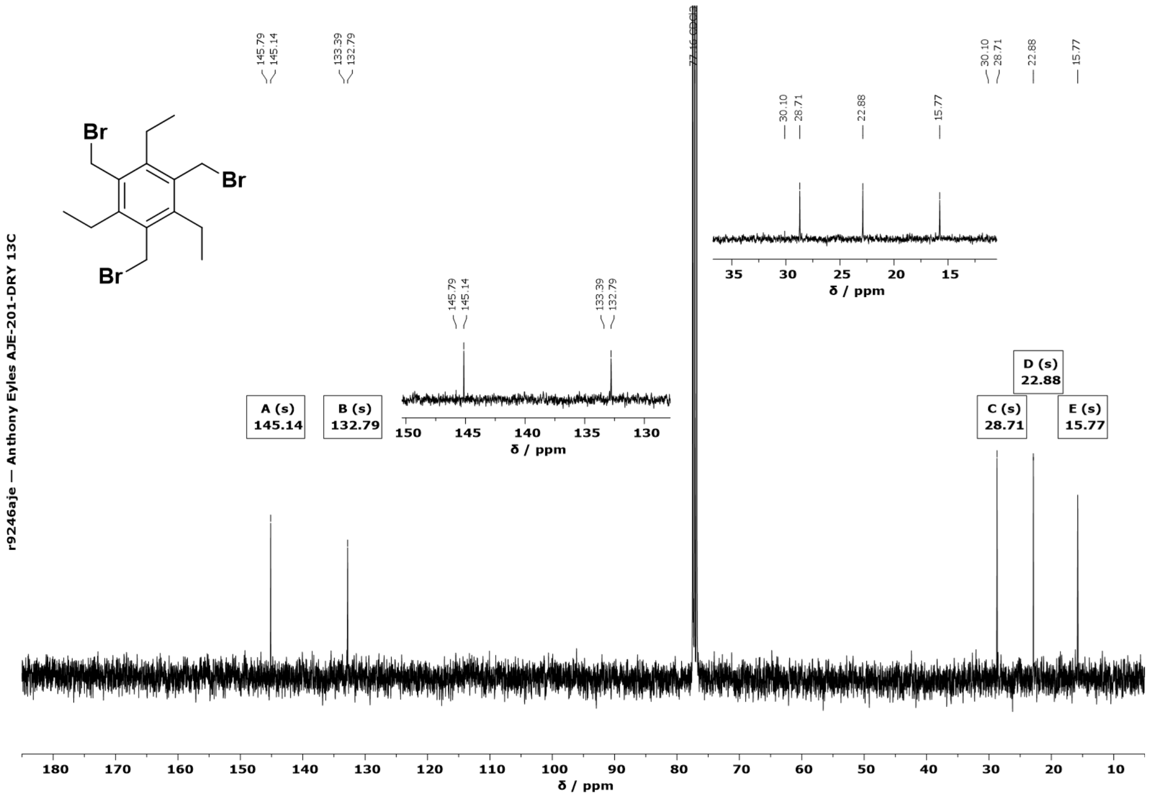

Figure S50 ^{13}C NMR ($CDCl_3$, 101 MHz, 1024 scans) spectrum for 1,3,5-tris(bromomethyl)-2,4,6-triethyl-benzene.

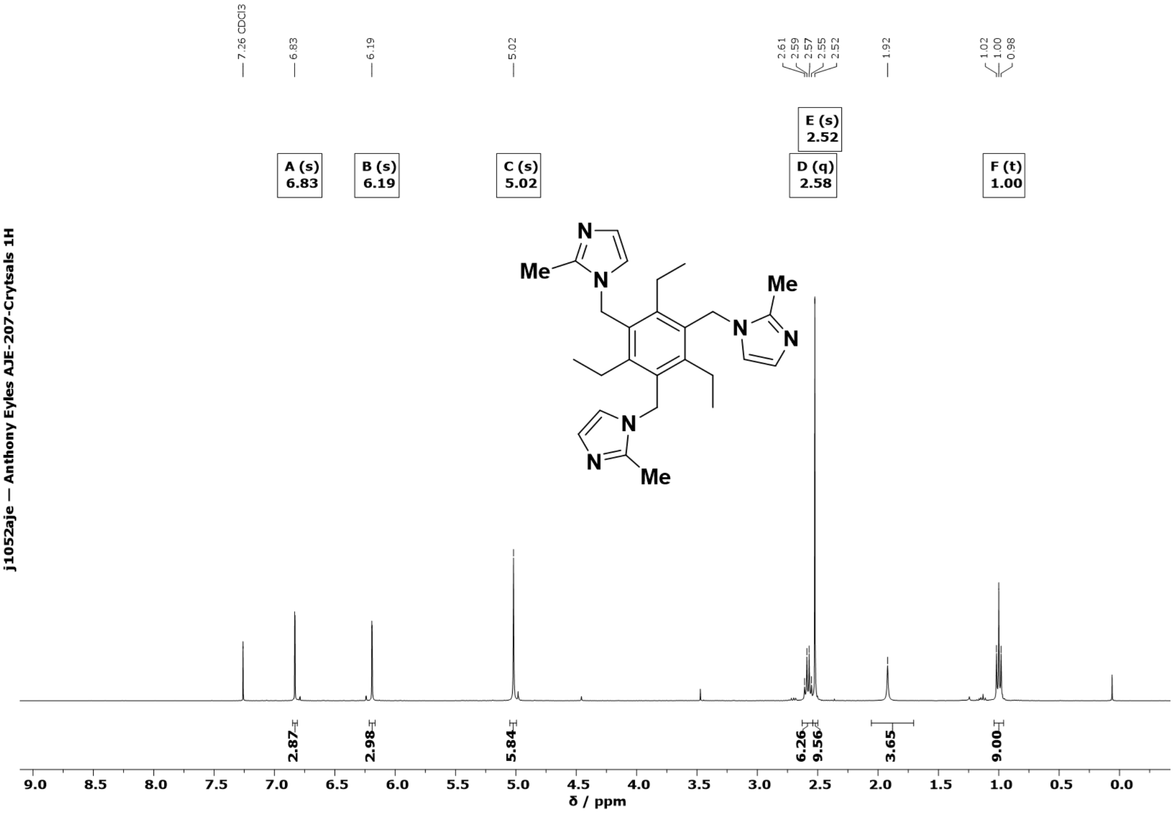

Figure S51 ^{1}H NMR($CDCl_3$,400 MHz, 8 scans) of 1,3,5-tris(methyl(2-methylimidazole))-2,4,6-triethylbenzene.

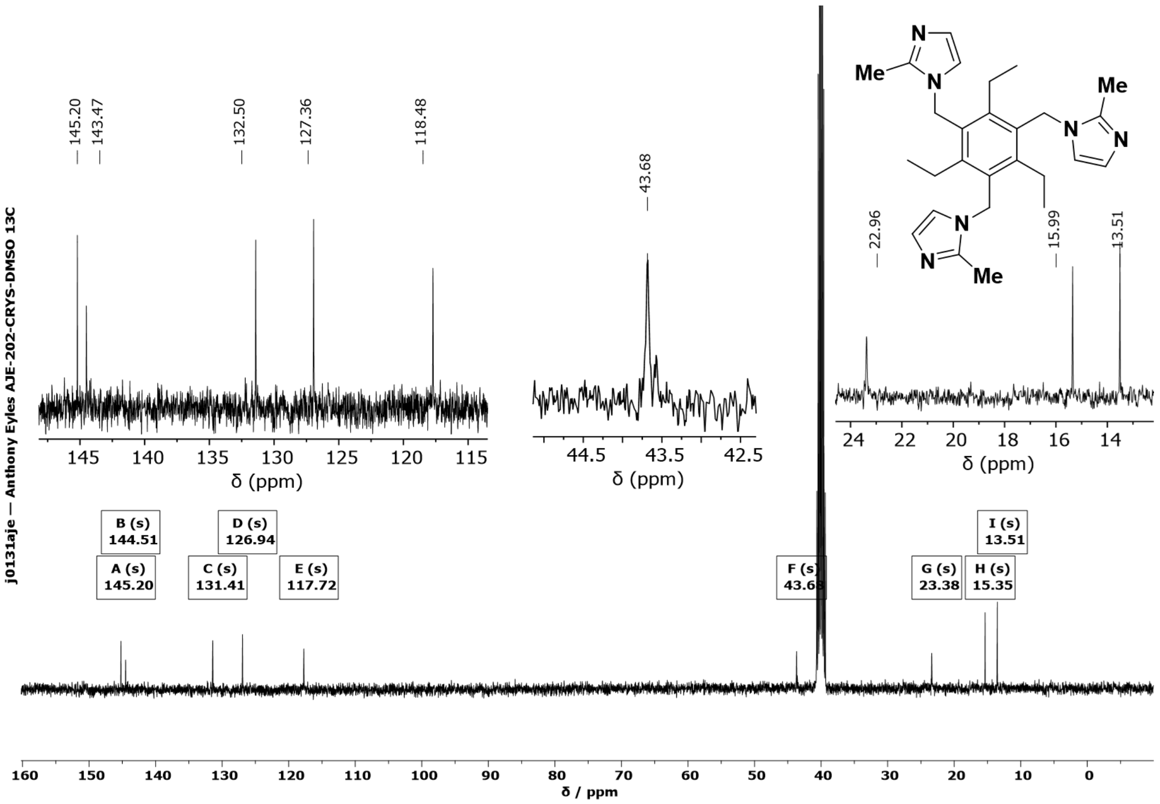

Figure S52 ^{13}C NMR (DMSO-*d_6*, 101 MHz, 1024 scans) of 1,3,5-tris(methyl(2-methylimidazole))-2,4,6-triethylbenzene.

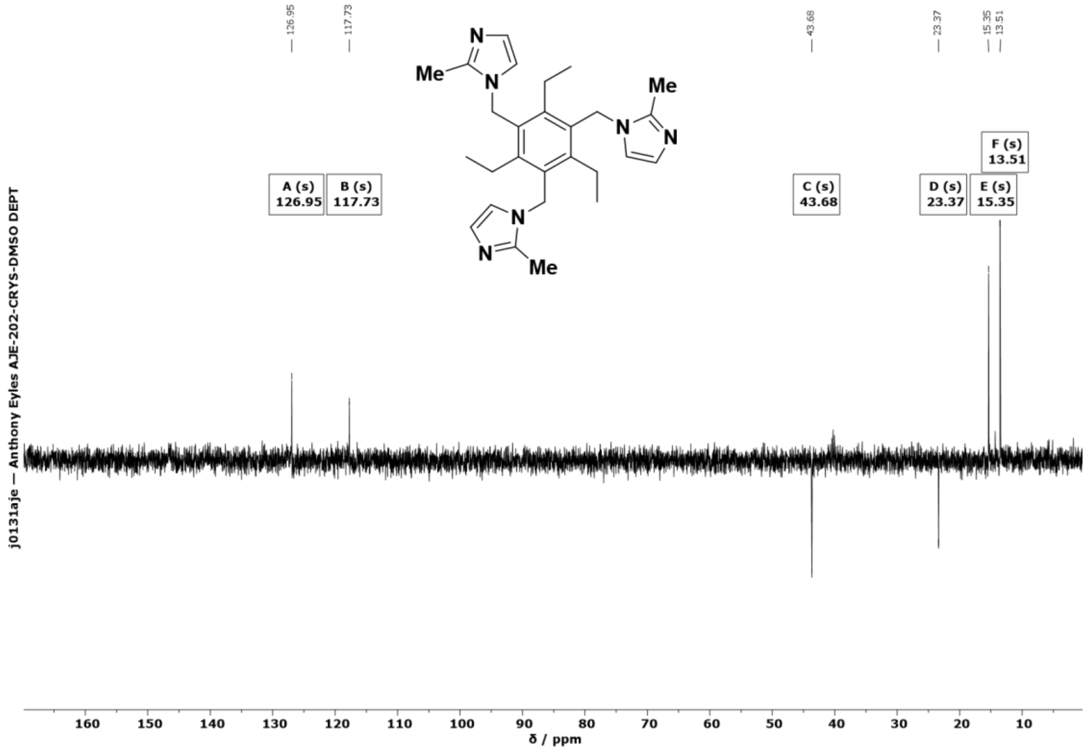

Figure S53 ^{13}C DEPT 135 Spectrum (DMSO-*d_6*, 101 MHz) of 1,3,5-tris(methyl(2-methylimidazole))-2,4,6-triethylbenzene.

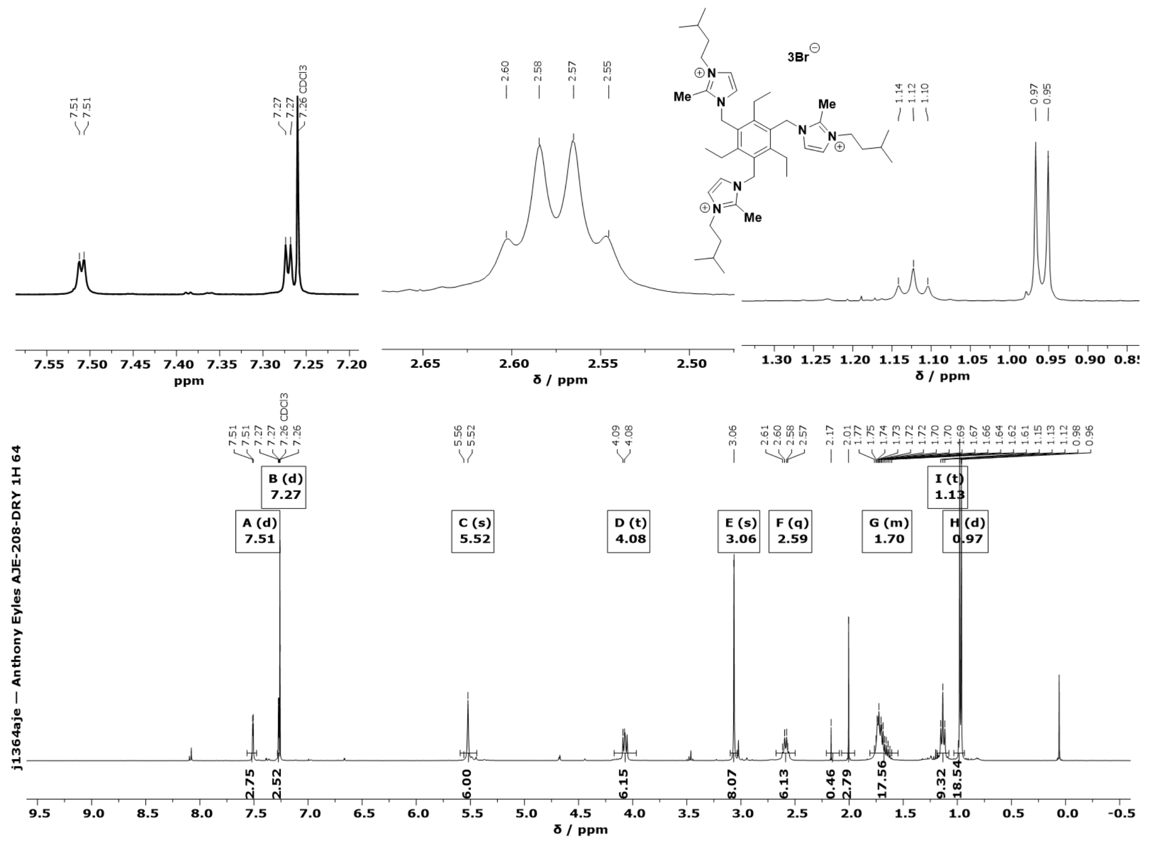

Figure S54 ^{1}H NMR ($CDCl_3$, 400 MHz, 64 scans) of TrisImid.3Br (6).

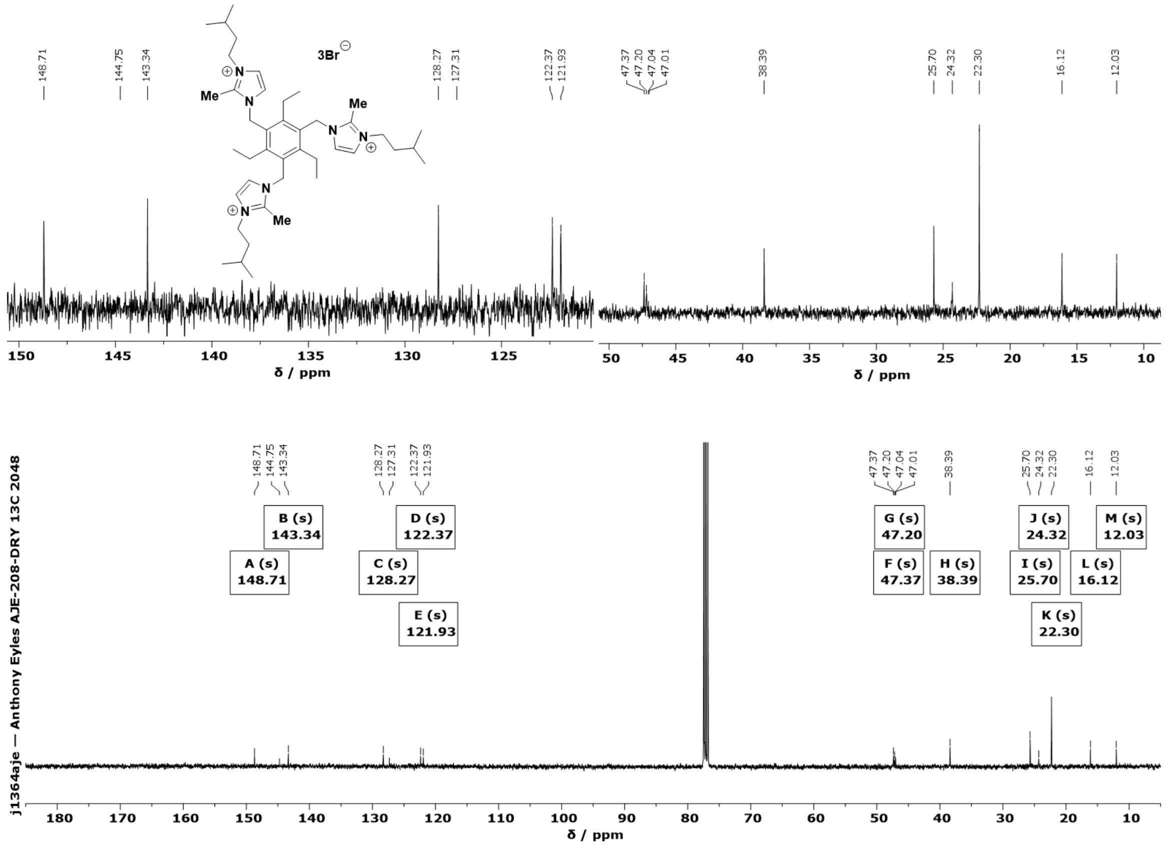

Figure S55 ^{13}C NMR ($CDCl_3$, 101 MHz, 2048 scans) of TrisImid.3Br (6).

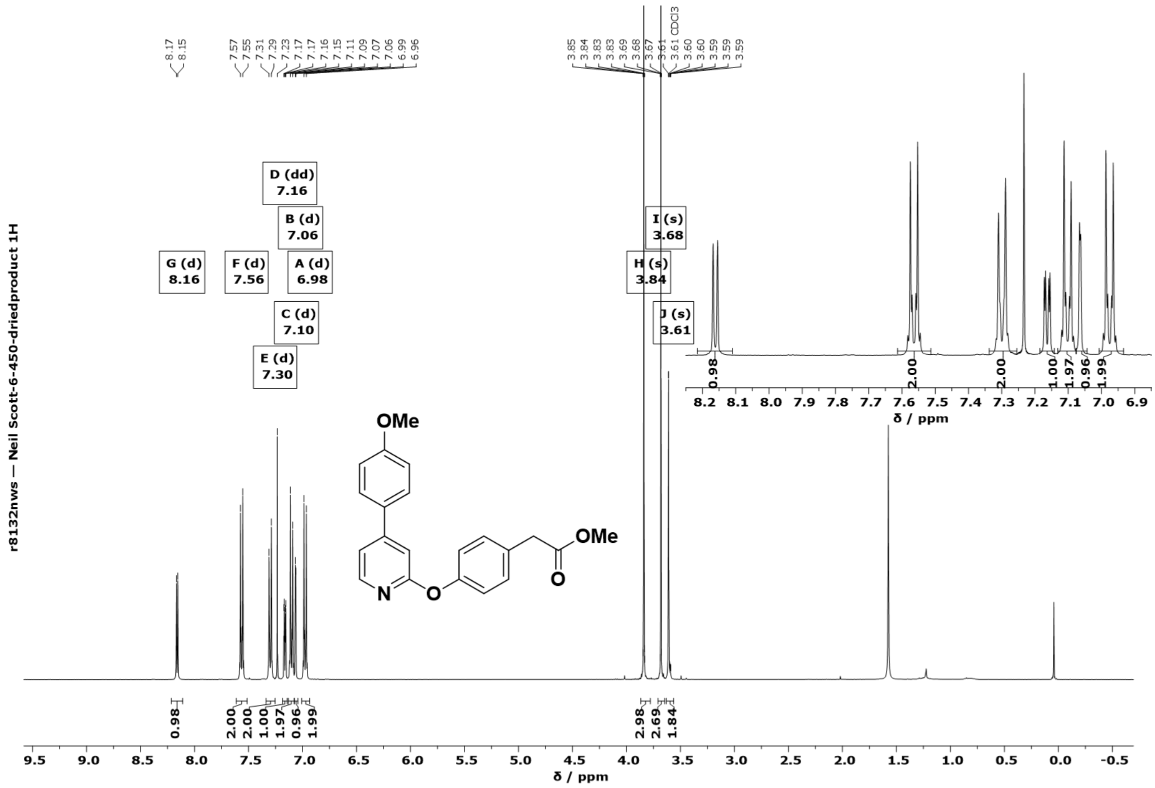

Figure S56 ^{1}H NMR ($CDCl_3$,400 MHz, 128 scans) of 4[4-anisyl, 2-pyridinyl}oxy]-benzene acetic acid methyl ester.

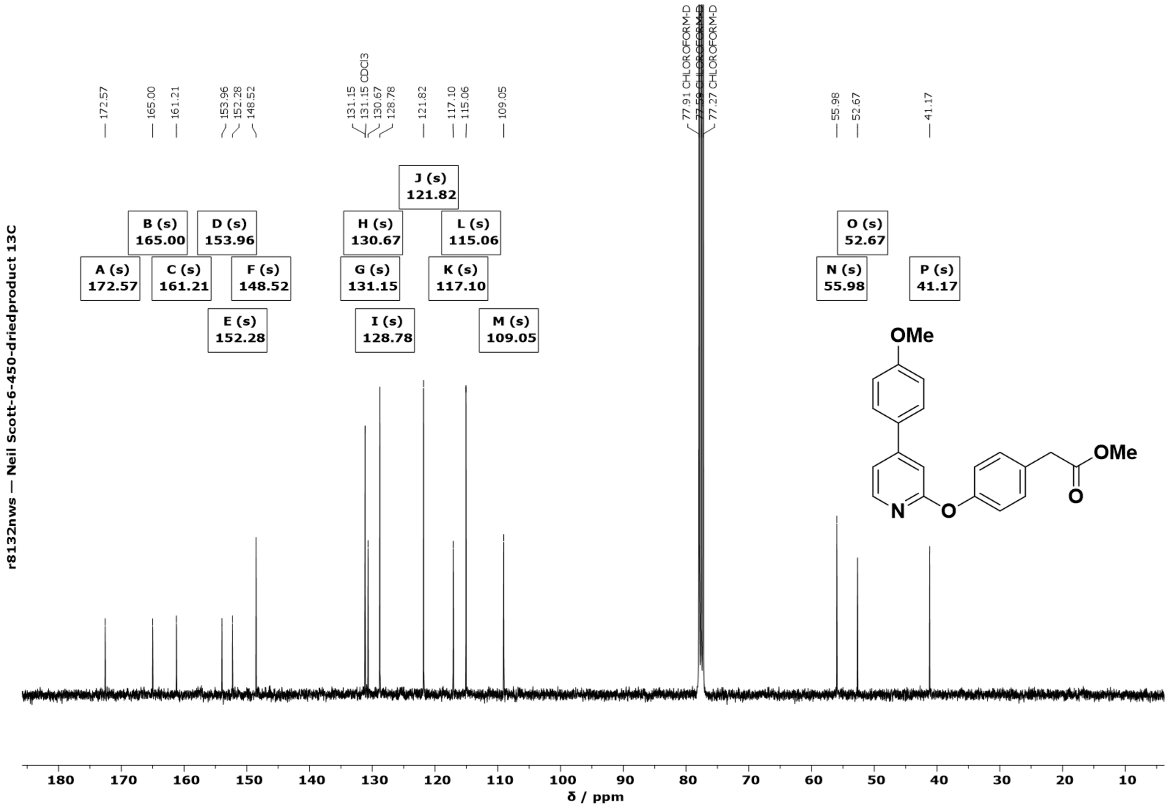

Figure S57 ^{13}C NMR ($CDCl_3$, 101 MHz, 2048 scans) of 4[4-anisyl, 2-pyridinyl}oxy]-benzene acetic acid methyl ester.

6. References

1. Scott, N. W.; Ford, M. J.; Schotes, C.; Parker, R. R.; Whitwood, A. C.; Fairlamb, I. J. S. The ubiquitous cross-coupling catalyst system ‘Pd $(OAc)_2$’/2PPh_3 forms a unique dinuclear PdI complex: an important entry point into catalytically competent cyclic Pd3 clusters. *Chem. Sci.* **2019**, *10*, 7898-7906.
2. Coulson, D. R.; Grim, S. O. Tetrakis(triphenylphosphine)palladium(0). In *Inorganic Syntheses*, Cotton, F. A., Ed. McGraw-Hill, Inc: 1972; pp 121-124.
3. Sicre, C.; Alonso-Gómez, J. L.; Cid, M. M. Regioselectivity in alkenyl(aryl)-heteroaryl Suzuki cross-coupling reactions of 2,4-dibromopyridine. A synthetic and mechanistic study. *Tetrahedron* **2006**, *62*, 11063-11072.
4. Fulmer, G. R.; Miller, A. J. M.; Sherden, N. H.; Gottlieb, H. E.; Nudelman, A.; Stoltz, B. M.; Bercaw, J. E.; Goldberg, K. I. NMR Chemical Shifts of Trace Impurities: Common Laboratory Solvents, Organics, and Gases in Deuterated Solvents Relevant to the Organometallic Chemist. *Organometallics* **2010**, *29*, 2176-2179.
5. Stephenson, T. A.; Wilkinson, G. Acetato complexes of palladium(II). *J. Inorg. Nucl. Chem.* **1967**, *29*, 2122-2123.
6. Wu, X.; Zhang, J.; Liu, S.; Gao, Q.; Wu, A. An Efficient Synthesis of Polysubstituted Pyridines via C–H Oxidation and C–S Cleavage of Dimethyl Sulfoxide. *Adv. Synth. Catal.* **2016**, *358*, 218-225.
7. Gujjarappa, R.; Vodnala, N.; Kumar, M.; Malakar, C. C. Pd-Catalyzed Decarboxylation and Dual C(sp3)–H Functionalization Protocols for the Synthesis of 2,4-Diarylpyridines. *J. Org. Chem.* **2019**, *84*, 5005-5020.
8. Chen, H.; Deng, S.; Wang, Y.; Albadari, N.; Kumar, G.; Ma, D.; Li, W.; White, S. W.; Miller, D. D.; Li, W. Structure–Activity Relationship Study of Novel 6-Aryl-2-benzoyl-pyridines as Tubulin Polymerization Inhibitors with Potent Antiproliferative Properties. *J. Med. Chem.* **2020**, *63*, 827-846.
9. Chambron, J.-C.; Sauvage, J.-P. Synthèse de composés macrocycliques comportant deux fragments coordinants séparés et différents,de type bipyrldyl-2,2' et diphényl-2,9 phénanthroline-1,10. *Tetrahedron* **1987**, *43*, 895-904.
10. Sim, J.; Viji, M.; Rhee, J.; Jo, H.; Cho, S. J.; Park, Y.; Seo, S.-Y.; Jung, K.-Y.; Lee, H.; Jung, J.-K. γ-Functionalization of α,β-Unsaturated Nitriles under Mild Conditions: Versatile Synthesis of 4-Aryl-2-Bromopyridines. *Adv. Synth. Catal.* **2019**, *361*, 5458-5465.
11. Hagui, W.; Soulé, J.-F. Synthesis of 2-Arylpyridines and 2-Arylbipyridines via Photoredox-Induced Meerwein Arylation with in Situ Diazotization of Anilines. *J. Org. Chem.* **2020**, *85*, 3655-3663.
12. Bellan, A. B.; Knochel, P. Highly Regioselective Remote Lithiation of Functionalized 1,3-bis-Silylated Arenes. *Angew. Chem. Int. Ed.* **2019**, *58*, 1838-1841.
13. Wallace, K. J.; Hanes, R.; Anslyn, E.; Morey, J.; Kilway, K. V.; Siegel, J. Preparation of 1,3,5-Tris(aminomethyl)-2,4,6-triethylbenzene from Two Versatile 1,3,5-Tri(halosubstituted) 2,4,6-Triethylbenzene Derivatives. *Synthesis* **2005**, *2005*, 2080-2083.
14. Sather, A. C.; Berryman, O. B.; Rebek, J. Selective Recognition and Extraction of the Uranyl Ion. *J. Am. Chem. Soc.* **2010**, *132*, 13572-13574.
15. Willans, C. E.; Anderson, K. M.; Junk, P. C.; Barbour, L. J.; Steed, J. W. A small tris(imidazolium) cage forms an N-heterocyclic carbene complex with silver(I). *Chem. Commun.* **2007**, 3634-3636.
16. Planellas, M.; Pleixats, R.; Shafir, A. Palladium Nanoparticles in Suzuki Cross-Couplings: Tapping into the Potential of Tris-Imidazolium Salts for Nanoparticle Stabilization. *Adv. Synth. Catal.* **2012**, *354*, 651-662.
17. Zhao, X.; Chen, H.; Xing, S.; Yuan, W.; Wu, L.; Chen, X.; Zhan, C.-G. Regioselective synthesis of 2- and 4-diarylpyridine ethers and their inhibitory activities against phosphodiesterase 4B. *J. Mol. Struct.* **2019**, *1196*, 455-461.
18. CrysAlisPro, Oxford Diffraction Ltd. Version 1.171.34.41
19. Empirical absorption correction using spherical harmonics, implemented in SCALE3 ABSPACK scaling algorithm within CrysAlisPro software, Oxford Diffraction Ltd. Version 1.171.34.40
20. Dolomanov, O. V.; Bourhis, L. J.; Gildea, R. J.; Howard, J. A. K.; Puschmann, H. OLEX2: a complete structure solution, refinement and analysis program. *J. Appl. Cryst.* **2009**, *42*, 339-341.
21. Sheldrick, G., SHELXT - Integrated space-group and crystal-structure determination. *Acta Crystallogr. A* **2015**, *71*, 3-8.
22. Sheldrick, G. Crystal structure refinement with SHELXL. *Acta Crystallogr. C* **2015**, *71*, 3-8.
